# Diffuse interface model for two-phase flows on evolving surfaces with different densities: global well-posedness

**DOI:** 10.1007/s00526-025-03001-w

**Published:** 2025-05-04

**Authors:** Helmut Abels, Harald Garcke, Andrea Poiatti

**Affiliations:** 1 Fakultät für Mathematik, Universität Regensburg, 93040 Regensburg, Germany; 2 Faculty of Mathematics, University of Vienna, Oskar-Morgenstern-Platz 1, 1090 Vienna, Austria

**Keywords:** 35Q30, 76D05, 35D35, 35Q35, 76T06

## Abstract

We show global in time existence and uniqueness on any finite time interval of strong solutions to a Navier–Stokes/Cahn-Hilliard type system on a given two-dimensional evolving surface in the case of different densities and a singular (logarithmic) potential. The system describes a diffuse interface model for a two-phase flow of viscous incompressible fluids on an evolving surface. We also establish the validity of the instantaneous strict separation property from the pure phases. To show these results we use our previous achievements on local well-posedness together with suitable novel regularity results for the convective Cahn-Hilliard equation. The latter allows to obtain higher-order energy estimates to extend the local solution globally in time. To this aim the time evolution of energy type quantities has to be calculated and estimated carefully.

## Introduction

Two-phase flows and phase separation phenomena play an important role in the sciences and engineering applications. In the present contribution we continue our study in [4] of a diffuse interface model for the two-phase of incompressible fluids on an evolving interface $\mathcal {S}:=\mathop \bigcup \limits _{t\in [0,T]}\Gamma (t)\times \{t\}$, $T>0$, for different densities in the case that the evolving interface is given. The model leads to the following Navier–Stokes/Cahn-Hilliard system for $({\textbf {v}},\pi ,\varphi ,\mu )$ on *S*:

1.1 $$\begin{aligned} {\left\{ \begin{array}{ll} \rho {\textbf {P}}\partial ^\circ _t{\textbf {v}}+((\rho {\textbf {v}}+{\textbf {J}}_\rho )\cdot \nabla _\Gamma ){\textbf {v}}+\rho v_{\textbf {n}}{\textbf {H}}{\textbf {v}}+v_{\textbf {n}}{\textbf {H}}{\textbf {J}}_\rho -2{\textbf {P}}\textrm{div}_\Gamma (\nu (\varphi ){\mathcal {E}}_S({\textbf {v}}))+\nabla _\Gamma \pi \\ \quad =-{\textbf {P}}\textrm{div}_\Gamma (\nabla _\Gamma \varphi \otimes \nabla _\Gamma \varphi )+2{\textbf {P}}\textrm{div}_\Gamma (\nu (\varphi )v_{\textbf {n}}{\textbf {H}})+\frac{\rho }{2}\nabla _\Gamma (v_{\textbf {n}})^2,\\ \textrm{div}_\Gamma {\textbf {v}}=-Hv_{\textbf {n}}, \\ \partial ^\circ _t\varphi -\textrm{div}_\Gamma (m(\varphi )\nabla _\Gamma \mu )+\nabla _\Gamma \varphi \cdot {\textbf {v}}=0,\\ \mu =-\Delta _\Gamma \varphi +\Psi '(\varphi ), \end{array}\right. } \end{aligned}$$

with ${\textbf {J}}_\rho =-\frac{\widetilde{\rho }_1-\widetilde{\rho }_2}{2}\nabla _\Gamma \mu$. Here, $v_{\textbf {n}}$ is the normal velocity of $\Gamma$, which is assumed to coincide with the normal component of ${\textbf {v}}$ satisfying the compatibility condition

$$\begin{aligned} \int _{\Gamma (t)}Hv_{\textbf {n}}d\sigma =0 \qquad \text {for all }t\in [0,T]. \end{aligned}$$

Moreover, *H* and ${\textbf {H}}$ at time *t* are the mean curvature and the Weingarten map of $\Gamma (t)\subseteq \mathbb {R}^3$ and ${\textbf {P}}(x,t)$ is the orthogonal projection onto $T_x\Gamma (t)$ for $(x,t)\in \mathcal {S}$, whereas $\partial ^\circ _t$ denotes the material time derivative (see the section below for a precise definition). These quantities are determined by the given evolving surface $\mathcal {S}$. Moreover, ${\textbf {v}}:\mathcal {S}\rightarrow \mathbb {R}^3$ with ${\textbf {v}}(x,t)\in T_{x}\Gamma (t)$ for all $(x,t)\in \mathcal {S}$ is the tangential (mean) velocity of the fluid mixture, $\pi :\mathcal {S} \rightarrow \mathbb {R}$ is its pressure, and ${\mathcal {E}}_S({\textbf {v}})$ is the surface rate-of-strain tensor, defined as the symmetrized tangential velocity gradient of ${\textbf {v}}$. Furthermore, $\varphi :\mathcal {S} \rightarrow [-1,1]$ is the volume fraction difference of the fluids, $\mu :\mathcal {S} \rightarrow \mathbb {R}$ is the chemical potential and $\Psi :[-1,1]\rightarrow \mathbb {R}$ a homogeneous free energy density of the mixture. Also, $\rho =\rho (\varphi ):=\tfrac{{\tilde{\rho }}_1+{\tilde{\rho }}_2}{2}+ \tfrac{{\tilde{\rho }}_1-{\tilde{\rho }}_2}{2}\varphi$ is the mass density of the mixture, where ${\tilde{\rho }}_j>0$ is the specific density of fluid $j=1,2$. For simplicity we will set the mobility $m(\varphi )\equiv 1$. More details on the notation are given in Sect. 2 below. We refer to [4] for the derivation of this model and references on related models and their analysis. We note that, as obtained in the derivation of the model in [4], the density $\rho$ satisfies

1.2 $$\begin{aligned} \partial ^\circ _t\rho +\textrm{div}_\Gamma ({\textbf {J}}_\rho )+\textrm{div}_\Gamma (\rho {\textbf {u}})=0. \end{aligned}$$

In [4] existence and uniqueness of strong solutions locally in time for sufficiently regular initial data was shown. It is the purpose of this contribution to extend this result and show existence of strong solution globally in time in the case of singular potential $\Psi$, as well as to establish the validity of the instantaneous strict separation property of $\varphi$ from the pure phases. If the Navier–Stokes/Cahn-Hilliard system (1.1) is considered in two-dimensional Euclidean space instead of a two-dimensional evolving surface $\Gamma (t)\subseteq \mathbb {R}^3$, the corresponding result was shown by Giorgini [17] in the case of periodic boundary conditions and by Abels, Garcke, and Giorgini in [2] in the case of a bounded sufficiently smooth domain $\Omega \subseteq \mathbb {R}^2$. In the latter reference also results on the case of three space dimensions and further references for Navier–Stokes/Cahn-Hilliard systems in Euclidean space can be found. In bounded domains we also refer to Abels, Garcke, and Poiatti [3] for the extension of the analysis to multi-phase flows and to [15] for its nonlocal counterpart. We note that in the case of same densities, i.e., $\rho \equiv const.$, global well-posedness of weak solutions was shown in Elliott and Sales [14] by combining some techniques developed by Caetano and Elliott [10] and Olshanskii, Reusken, and Zhiliakov [20]. In the latter contribution global existence and uniqueness of weak solutions for a (tangential) Navier–Stokes system on an evolving given surface is shown (we refer to [9] for a review on the modeling of tangential Navier–Stokes equations on evolving surfaces).

In order to prove our main result on the existence of global strong solutions we will use similar arguments as in [2], but in the present situation all the results and arguments have to be generalized to the more delicate case of an evolving surface $\Gamma (t)$. To this end we also use some techniques and identities as by Caetano, Elliott, Grasselli, and Poiatti [11] for the Cahn-Hilliard equation with a singular potential on an evolving surface. We conclude the introduction by observing that, in accordance to the results obtained in [2] for three-dimensional bounded domains, in the (unphysical) case of a three-dimensional evolving surface $\Gamma (t)\subset \mathbb {R}^4$, one could extend the techniques developed in this contribution to obtain the existence and uniqueness of local-in-time strong solutions to system (1.1). We do not expect to be able to extend the strong solutions globally in time in general, since this is an open problem in the case of a flat domain in $\mathbb {R}^3$.

The structure of the present contribution is as follows. In Sect. 2 we summarize some basic notation, assumptions and preliminary results. The main result is stated in Sect. 3. The first important step for the proof of the main result is to generalize the regularity results for the convective Cahn-Hilliard equation shown in [2] to the case of an evolving surface, which is done in Sect. 4. Finally, in Sect. 5 the main result is proven.

## Assumptions and functional setting

### Flow map and surface piola transform

We use the same notation and assumptions as in [4]. In particular we assume that $\mathcal {S}= \bigcup _{0\le t\le T}\{t\}\times \Gamma (t)$ is a $C^6$-manifold. Moreover, we have a normal flow map $\Phi ^n_{(\cdot )}\in C^4(\mathcal {S}_0,\mathcal {S})$, where $\mathcal {S}_0= [0,T_0]\times \Gamma _0$, which solves

$$\begin{aligned}&\frac{d}{dt}\Phi _t^n({{\textbf {z}}})={{\textbf {V}}}_{\textbf {n}}(t,\Phi _t^n({{\textbf {z}}})),\\  &\Phi _0^n({{\textbf {z}}})={{\textbf {z}}}, \end{aligned}$$

for all ${{\textbf {z}}}\in \Gamma _0$, $t\in [0,T]$, where ${{\textbf {n}}}\in C^5([0,T]\times \mathbb {R}^3;\mathbb {R}^3)$ and ${\textbf {n}}(t,\cdot )$ is a normal field to $\Gamma (t)$ for every $t\in [0,T]$. By $\Phi _{-t}:\Gamma (t)\rightarrow \Gamma _0$ we denote the inverse of $\Phi _t:\Gamma _0\rightarrow \Gamma (t)$ for $t\in [0,T]$. Moreover, we define ${\textbf {D}}={\textbf {D}}(t,{{\textbf {z}}}):=D\Phi _t^n({{\textbf {z}}}){\textbf {P}}_0:\mathbb {R}^3\rightarrow \mathbb {R}^3$ for every $t\in [0,T], {{\textbf {z}}}\in \Gamma _0$, where ${\textbf {P}}_0={{\textbf {I}}}-({\textbf {n}}_0\otimes {\textbf {n}}_0)$ is the orthogonal projection onto $T_{{{\textbf {z}}}}\Gamma _0$, and ${\textbf {D}}^{-}={\textbf {D}}^{-}(t,{{\textbf {x}}}):= D\Phi _{-t}^n({{\textbf {x}}}){\textbf {P}}:\mathbb {R}^3\rightarrow \mathbb {R}^3$, where ${\textbf {P}}={{\textbf {I}}}-({\textbf {n}}\otimes {\textbf {n}})$ is the orthogonal projection onto $T_{{{\textbf {x}}}}\Gamma (t)$. Furthermore, we define ${\textbf {A}}={\textbf {A}}(t,{{\textbf {z}}}):\mathbb {R}^3\rightarrow \mathbb {R}^3$ by

2.1 $$\begin{aligned} {\textbf {A}}(t,{{\textbf {z}}}):=J^{-1}{{\textbf {D}}}(t,{{\textbf {z}}})+{\textbf {n}}\otimes {\textbf {n}}_0\qquad \text {for all }t\in [0,T], {{\textbf {z}}}\in \Gamma _0, \end{aligned}$$

which is invertible with inverse ${\textbf {A}}^{-1}(t,{{\textbf {z}}}):=J{\textbf {D}}^{-}+{\textbf {n}}_0\otimes {\textbf {n}}$. Then we have

2.2 $$\begin{aligned} \left\| J\right\| _{C^4(\mathcal {S}_0)}+\left\| J^{-1}\right\| _{C^4(\mathcal {S})}+\left\| {{\textbf {D}}}\right\| _{C^4(\mathcal {S}_0)}+\left\| {\textbf {D}}^{-}\right\| _{C^4(\mathcal {S})}+\left\| {\textbf {A}}\right\| _{C^4(\mathcal {S}_0)}+\left\| {\textbf {A}}^{-1}\right\| _{C^4(\mathcal {S})}\le C(T). \end{aligned}$$

We now denote by $\nabla _\Gamma$ the tangential gradient, which is for a vector field defined as the covariant derivative, i.e., $\nabla _\Gamma :={\textbf {P}}\nabla {\textbf {P}}$, where $\nabla$ the gradient of the function defined on $\Gamma$ and extended in an open neighborhood $U\subseteq \mathbb {R}^3$ of $\Gamma$. Given a differentiable vector field ${\textbf {v}}:S\rightarrow \mathbb {R}^3$, we also define $(\widehat{\nabla }_\Gamma {\textbf {v}})_{ij}:=(\nabla _\Gamma v_i)_j$, $i,j=1,2,3$. Then $\nabla _\Gamma {\textbf {v}}={\textbf {P}}\widehat{\nabla }_\Gamma {\textbf {v}}$. For of a differentiable vector field ${\textbf {v}}:S\rightarrow \mathbb {R}^3$, the tangential divergence is then defined as $\textrm{div}_\Gamma {\textbf {v}}:=\textrm{tr}(\nabla _\Gamma {\textbf {v}})$, whereas for a matrix-valued differentiable ${\textbf {A}}:S\rightarrow \mathbb {R}^{3\times 3}$ we define $\textrm{div}_\Gamma {\textbf {A}}:=\{\textrm{div}_\Gamma ({{\textbf {e}}}_i^T{\textbf {A}})\}_{i=1,2,3}$, where $\{{{\textbf {e}}}_i\}_i$ is the canonical basis of $\mathbb {R}^3$.

We note that ${\textbf {H}}:=\nabla _\Gamma {\textbf {n}}$ and $H=\text {tr}{\textbf {H}}$. Thus

2.3 $$\begin{aligned} \left\| H\right\| _{C^4(\mathcal {S})}+\left\| v_{\textbf {n}}\right\| _{C^4(\mathcal {S})}\le C(T). \end{aligned}$$

Let us recall the definitions of pullback and pushforward maps. For ${{\textbf {f}}}:\mathcal {S}_0\rightarrow \mathbb {R}^q$, for some $q\in \mathbb {N}$,

$$ (\widetilde{\phi }_{t}{{\textbf {f}}})(t,{{\textbf {x}}})={{\textbf {f}}}(t,\Phi _{-t}^n({{\textbf {x}}})), $$

and for any ${{\textbf {g}}}:\mathcal {S}\rightarrow \mathbb {R}^q$,

$$ (\widetilde{\phi }_{-t}{{\textbf {g}}})(t,{{\textbf {z}}}):={{\textbf {g}}}(t,\Phi _t^n({{\textbf {z}}})). $$

Furthermore surface Piola transform of ${{\textbf {f}}}:\mathcal {S}_0\rightarrow \mathbb {R}^3$ is defined by

$$ (\phi _{t}{{\textbf {f}}})(t,{{\textbf {x}}})={\textbf {A}}(t,\Phi _{-t}^n({{\textbf {x}}})){{\textbf {f}}}(t,\Phi _{-t}^n({{\textbf {x}}}))={\textbf {A}}(t,\Phi _{-t}^n({{\textbf {x}}}))(\widetilde{\phi }_t{{\textbf {f}}})(t,{{\textbf {x}}}), $$

as well as its inverse is defined for ${{\textbf {g}}}:\mathcal {S}\rightarrow \mathbb {R}^3$ by

$$ (\phi _{-t}{{\textbf {g}}})(t,{{\textbf {z}}}):={\textbf {A}}^{-1}(t,\Phi _t^n({{\textbf {z}}})){{\textbf {g}}}(t,\Phi _t^n({{\textbf {z}}}))={\textbf {A}}^{-1}(t,\Phi _t^n({{\textbf {z}}}))(\widetilde{\phi }_{-t}{\varvec{{g}}})(t,{{\textbf {z}}}). $$

We note that the surface Piola transform maps tangential vector fields to tangential vector field. Moreover, it has the property that $\text {div}_{\Gamma (t)}{{\textbf {f}}}=0$ for almost any ${{\textbf {x}}}\in \Gamma (t)$ if and only if $\text {div}_{\Gamma _0}\phi _{-t}{{\textbf {f}}}=0$ for almost any ${{\textbf {z}}}\in \Gamma _0$.

We refer to [4, Section 3] for further details and references.

### Evolving Banach and Hilbert spaces

We use the same notation for function spaces on evolving surfaces as in [4], which is based on [5, 6]. For the convenience of the reader we briefly recall important definitions. For any $t\in [0,T]$ we denote by $W^{k,p}(\Gamma (t))$, where $k\in \mathbb {N}$ and $1\le p\le \infty$, the standard Sobolev space and its norm by $\Vert \cdot \Vert _{W^{k,p}(\Gamma (t))}$ and as usual $H^{k}(\Gamma (t))=W^{k,2}(\Gamma (t))$. Furthermore, we set $H^{-1}(\Gamma (t))=(H^1(\Gamma (t)))'$.

For a vector space *X* of functions defined on $\Gamma (t)$, the space ${\textbf {X}}$ is the generic space of tangential vector field or matrix-valued funtions such that each component belongs to *X*. Moreover, we denote by $(\cdot ,\cdot )$ the inner product in ${\textbf {L}}^{2}(\Gamma (t))$ and by $\Vert \cdot \Vert$ the induced norm. The inner product of a Hilbert space *H* is denoted by $(\cdot ,\cdot )_{H}$ and $\Vert \cdot \Vert _{H}$ is its induced norm. Similarly as in [20], we introduce spaces of solenoidal vector fields

$$\begin{aligned}&{\textbf {L}}^2_\sigma (\Gamma (t)):=\{{\textbf {v}}\in {\textbf {L}}^2(\Gamma (t)): \textrm{div}_\Gamma {\textbf {v}}=0\text { a.e. on }\Gamma (t)\}, \\&{\textbf {H}}^1_\sigma (\Gamma (t)):=\{{\textbf {v}}\in {\textbf {H}}^1(\Gamma (t)): \textrm{div}_\Gamma {\textbf {v}}=0\text { a.e. on }\Gamma (t)\}. \end{aligned}$$

Notice that it holds the Hilbert triplet ${\textbf {H}}^1_\sigma (\Gamma (t))\hookrightarrow {\textbf {L}}^2_\sigma (\Gamma (t))\hookrightarrow {\textbf {H}}^1_\sigma (\Gamma (t))'$.

For a Banach space $X_0$ (independent of *t*) $L^q (0,T; X_0 )$ is the Bochner space of $X_0$-valued *q*-integrable (or essentially bounded) strongly measurable functions. The first order $X_0$-valued one-dimensional Sobolev space is denoted by $W^{1,p} (0, T; X_0)$, where $1 \le p < \infty$ and $H^1 (0, T; X_0) =W^{1,2}(0,T;X_0)$. Furthermore $C^{0,\alpha } (0,T; X)$ for $\alpha \in [0,1]$ is the Banach space of all Hölder continuous functions $f :[0,T]\rightarrow X_0$ with exponent $\alpha$.

Now, if *X*(*t*) is a generic scalar Banach space of functions over $\Gamma (t)$ (and ${{\textbf {X}}}(t)$ a space of tangential vector fields over $\Gamma (t)$), we have for mappings

$$\begin{aligned} u:[0, T]&\rightarrow \bigcup _{t\in [0,T]}\{t\}\times X(t), \quad t\mapsto (t,\bar{u}(t)) \end{aligned}$$

and $1\le p \le \infty$ that $u\in L^p_{X}$ if and only if $t\mapsto \widetilde{\phi }_{-t}\bar{u}(t)\in L^p(0, T; X_0)$ with the norm

$$\begin{aligned} \Vert u\Vert _{L^p_X} := {\left\{ \begin{array}{ll} \left( \int _0^T \Vert u(t)\Vert _{X(t)}^p dt\right) ^{1/p} & \text { if } p<\infty , \\ \text {ess sup}_{t\in [0,T]} \Vert u(t)\Vert _{X(t)} & \text { if } p=\infty . \end{array}\right. } \end{aligned}$$

Usually we identify *u*(*t*) and $\bar{u}(t)$. If $X(t)=H(t)$ are Hilbert spaces, we equip $L^2_H$ with the inner product

$$\begin{aligned} (u,v)_{L^2_{H}}&:= \int _0^T (u(t), v(t))_{H(t)}dt. \end{aligned}$$

For the spaces of tangential vector fields ${{\textbf {X}}}(t)={\textbf {L}}^2_\sigma (\Gamma (t)), {\textbf {H}}^1_\sigma (\Gamma (t)),{\textbf {H}}^2(\Gamma (t))$, we adopt as pullback the map $\phi _t$ and define, for $p\in [1,\infty ]$, ${\textbf {v}}\in L^p_{{{\textbf {X}}}}$ if $t\mapsto \phi _{-(\cdot )}\bar{{\textbf {v}}}(\cdot )\in L^p(0, T; {{\textbf {X}}}_0)$ with the norm

$$\begin{aligned} \Vert {\textbf {v}}\Vert _{L^p_{{\textbf {X}}}} := {\left\{ \begin{array}{ll} \left( \int _0^T \Vert {\textbf {v}}(t)\Vert _{{{\textbf {X}}}(t)}^p dt\right) ^{1/p} & \text { if } p<\infty , \\ \text {ess sup}_{t\in [0,T]} \Vert {\textbf {v}}(t)\Vert _{{{\textbf {X}}}(t)} & \text { if } p=\infty . \end{array}\right. } \end{aligned}$$

Furthermore, we have $u\in C^1_{X}$ if and only if $t\mapsto \widetilde{\phi }_{-t}u(t)\in C^1([0,T]; X_0)$ and we define its time derivative as

2.4 $$\begin{aligned} \partial ^{\bullet }_t u(t)=\partial ^\circ _tu(t)= \widetilde{\phi }_t \dfrac{d}{dt} \widetilde{\phi }_{-t} u(t). \end{aligned}$$

These spaces are equipped with the canonical norm

$$\begin{aligned} \Vert u\Vert _{C^1_{X}} := \sup _{t\in [0,T]} \Vert u(t)\Vert _{X(t)} + \sup _{t\in [0,T]} \Vert \partial ^\circ _tu(t)\Vert _{X(t)}. \end{aligned}$$

As in [20] we define for spaces of divergence-free vector fields ${{\textbf {X}}}(t)$ as ${\textbf {L}}^2_\sigma (\Gamma (t))$ and ${\textbf {H}}^1_\sigma (\Gamma (t))$ two time derivatives. The standard time derivative is defined as in (2.4), i.e., ${\textbf {v}}\in C^1_{{{\textbf {X}}}}$ if and only if $t\mapsto \widetilde{\phi }_{-t}u(t)\in C^1([0,T]; {{\textbf {X}}}_0)$ and

2.5 $$\begin{aligned} \partial ^{\bullet }_t {\textbf {v}}(t):=\partial ^\circ _t{\textbf {v}}(t):= \widetilde{\phi }_t \dfrac{d}{dt} \widetilde{\phi }_{-t} {\textbf {v}}(t). \end{aligned}$$

Note that this time derivative is not divergence-free in general. Therefore we also define the time derivative

$$\begin{aligned} \partial _t^*{\textbf {v}}:=\phi _t\frac{d}{dt}\phi _{-t}{\textbf {v}}(t), \end{aligned}$$

which is also consistent with the abstract framework developed in [5] when applied to the spaces ${\textbf {L}}^2_\sigma (\Gamma (t))$ and ${\textbf {H}}^1_\sigma (\Gamma (t))$ since it preserves divergence-free tangential vector fields. Because of [20, Lemma 3.6], we have the relation

2.6 $$\begin{aligned} \partial ^\circ _t{\textbf {v}}=\partial _t^*{\textbf {v}}-{\textbf {A}}(\partial ^\circ _t{\textbf {A}}^{-1}){\textbf {v}},\quad {\textbf {P}}\partial ^\circ _t{\textbf {v}}=\partial _t^*{\textbf {v}}-{\textbf {P}}{\textbf {A}}(\partial ^\circ _t{\textbf {A}}^{-1}){\textbf {v}}. \end{aligned}$$

These definitions give rise to two notions of weak time derivatives: For $u\in L^2_{H^1}$ a function $v\in L^2_{H^{-1}}$ is the weak time derivative of *u*, (denoted by $v=\partial ^\circ _tu$) if

$$\begin{aligned}&\int _0^T \langle v(t), \eta (t)\rangle _{H^{-1}(\Gamma (t)), H^1(\Gamma (t))} = -\int _0^T (u(t),\partial ^\circ _t\eta (t)) - \int _0^T \int _{\Gamma (t)} u(t)\eta (t) v_{\textbf {n}}Hd\sigma \end{aligned}$$

for every $\eta \in \mathcal {D}_{H^1}$ (where $\eta \in \mathcal {D}_{X}$ if and only if $t\mapsto \phi _{-t}u(t)\in C_0^\infty ((0,T); X_0)$). As usual the weak derivative $\partial ^\circ _tu$ coicides with classical derivative introduced before if *u* is continously differentiable. The same definition holds for the time derivative $\partial ^\circ _t{\textbf {v}}$ of a divergence-free tangential vector field ${\textbf {v}}$: we have ${\textbf {w}}=\partial ^\circ _t{\textbf {u}}$ in the weak sense if and only if

$$\begin{aligned}&\int _0^T \langle {\textbf {w}}(t), \varvec{\eta }(t)\rangle _{{\textbf {H}}^{1}_\sigma (\Gamma (t))',{\textbf {H}}^1_\sigma (\Gamma (t))} = -\int _0^T ({\textbf {v}}(t),\partial ^\circ _t\varvec{\eta }(t)) - \int _0^T \int _{\Gamma (t)} {\textbf {v}}(t)\cdot \varvec{\eta }(t) v_{\textbf {n}}Hd\sigma \end{aligned}$$

for any $\varvec{\eta }\in \mathcal {D}_{{{\textbf {H}}^1_\sigma }}$. Finally, because of (2.6) for smooth solutions, we define $\tilde{{\textbf {w}}}=\partial _t^*{\textbf {u}}$ if and only if

$$\begin{aligned}&\int _0^T \langle \tilde{{\textbf {w}}}(t), \varvec{\eta }(t)\rangle _{{\textbf {H}}^{1}_\sigma (\Gamma (t))',{\textbf {H}}^1_\sigma (\Gamma (t))}\\  &\quad = -\int _0^T ({\textbf {v}}(t),\partial _t^*\varvec{\eta }(t)) - \int _0^T \int _{\Gamma (t)} {\textbf {v}}(t)\varvec{\eta }(t) v_{\textbf {n}}Hd\sigma +\int _0^T\int _{\Gamma (t)}({\textbf {A}}\partial ^\circ _t({\textbf {A}}^{-1})\\&\qquad +({\textbf {A}}\partial ^\circ _t({\textbf {A}}^{-1}))^T){\textbf {v}}\cdot \varvec{\eta }d\sigma \end{aligned}$$

for every $\varvec{\eta }\in \mathcal {D}_{{{\textbf {H}}^1_\sigma }}$. Then (2.6) also holds for the weak notions of time derivatives.

We then define the Banach spaces

$$\begin{aligned} H^1_{{{\textbf {H}}^{1}_\sigma }'} := \left\{ {\textbf {v}}\in L^2_{{\textbf {H}}^{1}_\sigma } :\partial _t^*{\textbf {v}}\in L^2_{{{\textbf {H}}^{1}_\sigma }'}\right\} \, \text { with } \,\, \Vert {\textbf {v}}\Vert _{H^1_{{{\textbf {H}}^{1}_\sigma }'}} := \Vert {\textbf {v}}\Vert _{L^2_{{{\textbf {H}}^{1}_\sigma }}} + \Vert \partial _t^*{\textbf {v}}\Vert _{L^2_{{{\textbf {H}}^{1}_\sigma }'}}+\Vert {\textbf {v}}(0)\Vert , \end{aligned}$$

and

$$\begin{aligned} H^1_{H^{-1}} := \left\{ u\in L^2_{H^1} :\partial ^\circ _tu\in L^2_{H^{-1}}\right\} \, \text { with } \,\, \Vert u\Vert _{H^1_{H^{-1}}} := \Vert u\Vert _{L^2_{H^1}} + \Vert \partial ^\circ _tu \Vert _{L^2_{H^{-1}}}+\Vert u(0)\Vert . \end{aligned}$$

In the space $H^1_{{{\textbf {H}}^{1}_\sigma }'}$ one could also adopt the weak notion of the derivative $\partial ^\circ _t$, but in this case, since $\partial ^\circ _t{\textbf {v}}$ is not divergence-free, one should ask for $\partial ^\circ _t{\textbf {v}}_{|{\textbf {H}}^1_{\sigma }}\in L^2_{{{\textbf {H}}^1_\sigma }'}$. Notice that knowing only the action of $\partial ^\circ _t{\textbf {v}}$ on the functions in ${\textbf {H}}^1_\sigma (\Gamma (t))$ does not allow in principle to reconstruct the entire time derivative $\partial ^\circ _t{\textbf {v}}$, which has to be obtained from (2.6). On the other hand, if we know that $\partial _t^*{\textbf {v}}\in L^2_{{{\textbf {H}}^1_\sigma }'}$, then from (2.6) is immediate to infer $\partial ^\circ _t{\textbf {v}}\in L^2_{{{\textbf {H}}^1}'}$. We also define $H^1_{H^1}$, which denotes the space of those $u\in L^2_{H^1}$ which have a more regular weak time derivative $\partial ^\circ _tu\in L^2_{H^1}$. In general we denote by $W_{X,Y}$ the spaces of functions *u* such that $u\in L^2_X$ and $\partial ^\circ _tu\in L^2_Y$ when *X* and *Y* do not coincide, whereas we write $H^1_{X}:=W_{X,X}$. Furthermore, $W({\textbf {H}}^1_\sigma \cap {\textbf {H}}^2,{{\textbf {L}}^2_\sigma })$ denotes the space of those ${\textbf {v}}\in L^2_{{\textbf {H}}^1_\sigma \cap {\textbf {H}}^2}$ which have a more regular weak time derivative $\partial _t^*{\textbf {v}}\in L^2_{{\textbf {L}}^2_\sigma }$. Note that we prefer to define the space in this way, since if we consider in the definition of the last space the derivative $\partial ^\circ _t{\textbf {v}}$, this quantity would not be divergence-free nor tangential, whereas, as it is standard in the fixed domain case, we expect a divergence-free velocity time derivative to belong to ${\textbf {L}}^2_\sigma (\Gamma (t))$. Observe that it can be proven, exploiting the regularity of (2.2), that there is an evolving space equivalence in the sense of [5, Definition 2.31] between the spaces $H^1_{H^{-1}}$ and $H^1(0,T;H^{-1}(\Gamma _0))\cap L^2(0,T;H^1(\Gamma _0))$, as well as between $W(H^3,H^{-1})$ and $H^1(0,T;H^{-1}(\Gamma _0)\cap L^2(0,T;H^3(\Gamma _0))$, between $W(H^3,H^{1})$ and $H^1(0,T;H^{1}(\Gamma _0)\cap L^2(0,T;H^3(\Gamma _0))$, and between $W({H^{1},H^1})$ and $H^1(0,T;H^{1}(\Gamma _0))$. Analogously, by arguing for instance, in a similar way as in [20, Lemma 3.7], there is an evolving space equivalence between the spaces $W({\textbf {H}}^2\cap {\textbf {H}}^1_\sigma , {\textbf {L}}^2_\sigma )$ and $H^1(0,T;{\textbf {L}}^2_\sigma (\Gamma _0))\cap L^2(0,T;{\textbf {H}}^2(\Gamma _0))$. Clearly, the same evolving space equivalence holds between $W({\textbf {H}}^2, {\textbf {L}}^2)$ and $H^1(0,T;{\textbf {L}}^2(\Gamma _0))\cap L^2(0,T;{\textbf {H}}^2(\Gamma _0))$. Therefore, by standard embeddings for the Bochner spaces over $\Gamma _0$ and recalling these evolving space equivalences, we deduce the following

#### Lemma 2.1

The following embeddings hold

$$\begin{aligned}&H^1_{H^{-1}}\hookrightarrow C^0_{L^2},\quad H^1_{H^1}\hookrightarrow C^0_{H^1},\quad W_{H^3,H^{-1}}\hookrightarrow C^0_{H^1},\\  &W_{H^3,H^1}\hookrightarrow C^0_{H^2},\quad W({\textbf {H}}^2\cap {\textbf {H}}^1_\sigma , {\textbf {L}}^2_\sigma )\hookrightarrow C^0_{{\textbf {H}}^1_\sigma },\quad W({\textbf {H}}^2, {\textbf {L}}^2)\hookrightarrow C^0_{{\textbf {H}}^1}. \end{aligned}$$

Finally, if functions are not defined on the entire interval of time [0, *T*], but only on $[0,T_1]$, with $0<T_1\le T$, the corresponding spaces are denoted by an extra $(T_1)$ as for instance, $L^p_{X(T_1)}$. We note that Lemma 2.1 also holds for the spaces on $[0,T_1]$ and the embedding constants only depend on *T* if the norms of the initial value is included in the norm appropriately.

### Some uniform estimates on [0, *T*]

In this section we present some estimates which also hold uniformly in time on [0, *T*]. In particular, we have the following Poincaré inequality (see also [7]): there exists a constant $C_P(T)>0$ such that, for any $u\in H^1(\Gamma (t))$,

$$ \left\| u(t)-\overline{u}(t)\right\| \le C_P\left\| \nabla _{\Gamma }u\right\| ,\quad \forall t\in [0,T], $$

where $\overline{u}(t):=\dfrac{\int _{\Gamma (t)}u(t)d\sigma }{\left| \Gamma (t)\right| }$. Furthermore, we have the following adaptation of Korn’s inequality (see [20, Lemma 3.3]): there exists $C=C(T)>0$ such that, for any ${\textbf {v}}\in {\textbf {H}}^1(\Gamma (t))$, it holds

2.7 $$\begin{aligned} \left\| {\textbf {v}}\right\| _{{\textbf {H}}^1(\Gamma (t))}\le C(T)(\left\| {\textbf {v}}\right\| +\left\| {\mathcal {E}}_S({\textbf {v}})\right\| ),\quad \text { for a.a. }t\in [0,T]. \end{aligned}$$

Then, have the following Sobolev-Gagliardo-Nirenberg’s inequality:

2.8 $$\begin{aligned} \left\| f\right\| _{L^p(\Gamma (t))}\le C(T)\sqrt{p}\left\| f\right\| ^\frac{2}{p}\left\| f\right\| _{H^1(\Gamma (t))}^{1-\frac{2}{p}},\quad \forall p\in [2,+\infty ),\quad \forall f\in H^1(\Gamma (t)),\quad \forall t\in [0,T]. \end{aligned}$$

#### Proof

It is well known that, for any sufficiently regular open set $\Omega \subset \mathbb {R}^2$ it holds

$$ \left\| f\right\| _{L^p(\Omega )}\le C(\Omega )\sqrt{p}\left\| f\right\| ^\frac{2}{p}_{L^2(\Omega )}\left\| f\right\| _{H^1(\Omega )}^{1-\frac{2}{p}},\quad \forall p\in [2,+\infty ),\quad \forall f\in H^1(\Omega ). $$

It then holds, by a standard partition of unity argument applied to $\Gamma _0$, that there exists $C(\Gamma _0)>0$ such that

$$\begin{aligned} \left\| f\right\| _{L^p(\Gamma _0)}\le C(\Gamma _0)\sqrt{p}\left\| f\right\| ^\frac{2}{p}_{L^2(\Gamma _0)}\left\| f\right\| _{H^1(\Gamma _0)}^{1-\frac{2}{p}},\quad \forall p\in [2,+\infty ),\quad \forall f\in H^1(\Gamma _0). \end{aligned}$$

Thanks to the regularity of the flow map (2.2) and the compatibility of the space involved, we know that

$$\begin{aligned} \left\| f\right\| _{L^p(\Gamma (t))}&\le C(T)\left\| \widetilde{\phi }_{-t}f\right\| _{L^p(\Gamma _0)}\le C(T)\sqrt{p}\left\| \widetilde{\phi }_{-t}f\right\| ^\frac{2}{p}_{L^2(\Gamma _0)}\left\| \widetilde{\phi }_{-t}f\right\| _{H^1(\Gamma _0)}^{1-\frac{2}{p}}\\  &\le C(T)\sqrt{p}\left\| f\right\| ^\frac{2}{p}\left\| f\right\| _{H^1(\Gamma (t))}^{1-\frac{2}{p}}, \end{aligned}$$

concluding the proof. $\square$

Clearly (see also [20, Lemma 3.4]), the inequality (2.8) also follows for any (tangential) vector ${{\textbf {f}}}\in {\textbf {H}}^1(\Gamma (t))$ by considering it component-wise. By similar arguments we also deduce the validity of a uniform Agmon’s inequality (clearly also valid for tangential vector fields):

2.9 $$\begin{aligned} \left\| f\right\| _{L^\infty (\Gamma (t))}\le C(T)\left\| f\right\| ^\frac{1}{2}\left\| f\right\| _{H^2(\Gamma (t))}^\frac{1}{2}\quad \forall f\in H^2(\Gamma (t)),\quad \forall t\in [0,T]. \end{aligned}$$

In conclusion, let us assume that $f(t)\in H^2(\Gamma (t))$, for almost any $t\in [0,T]$, satisfies, for some $\tilde{\omega }>0$,

$$ -\Delta _\Gamma f(t)+\tilde{\omega }f(t)=h(t). $$

Then we can show that it holds

2.10 $$\begin{aligned} \left\| f(t)\right\| _{H^{k+2}(\Gamma (t))}\le C(T)\left\| h(t)\right\| _{H^k(\Gamma (t))},\quad k=1,2,\quad \text { for a.a. } t\in [0,T], \end{aligned}$$

where $C(T)>0$ does not depend on $t\in [0,T]$. The proof of this result exploits the elliptic regularity theory applied on each surface $\Gamma (t)$ as well as the regularity (2.2) on the flow map. For the sake of brevity we only refer to the proof of [4, Lemma 7.4] for a very similar argument.

### Results on time differentiation of some bilinear forms

We have the following essential theorem to deal with some variations on the classical Transport Theorem:

#### Theorem 2.2

Let the assumptions on the flow map $\Phi _t^n$ of Sect. 2.1 hold.

- For any $\eta , \phi \in H^1_{L^2}$ it holds
  2.11 $$\begin{aligned}&\frac{d}{dt}\int _{\Gamma (t)}\eta \phi d\sigma =\int _{\Gamma (t)}\partial ^\circ _t\eta \phi d\sigma +\int _{\Gamma (t)}\eta \partial ^\circ _t\phi d\sigma +\int _{\Gamma (t)}\eta \phi \ \textrm{div}_\Gamma {{\textbf {V}}}_{\textbf {n}}d\sigma . \end{aligned}$$
- For any $\eta ,\phi \in H^1_{H^1}$ it holds
  2.12 $$\begin{aligned}&\frac{d}{dt}\int _{\Gamma (t)}\nabla _\Gamma \eta \cdot \nabla _\Gamma \phi d\sigma =\int _{\Gamma (t)}\nabla _\Gamma \partial ^\circ _t\eta \cdot \nabla _\Gamma \phi d\sigma \\&\quad +\int _{\Gamma (t)}\nabla _\Gamma \eta \cdot \nabla _\Gamma \partial ^\circ _t\phi d\sigma +\int _{\Gamma (t)}\nabla _\Gamma \eta \cdot \nabla _\Gamma \phi \ \textrm{div}_\Gamma {{\textbf {V}}}_{\textbf {n}}d\sigma -2\int _{\Gamma (t)}\nabla _\Gamma \phi \cdot {\mathcal {E}}_S({{\textbf {V}}}_{\textbf {n}})\nabla _\Gamma \eta d\sigma .\nonumber \end{aligned}$$
- For any $f\in H^1_{H^1}$ and any ${\varvec{{g}}}\in H^1_{{\textbf {L}}^2}$ (${\varvec{{g}}}$ tangential vector field), it holds
  2.13 $$\begin{aligned}&\frac{d}{dt}\int _{\Gamma (t)}\nabla _\Gamma f\cdot {\varvec{{g}}}d\sigma \nonumber \\&\quad =\int _{\Gamma (t)}\nabla _\Gamma f\cdot \partial ^\circ _t{\varvec{{g}}}d\sigma +\int _{\Gamma (t)}\nabla _\Gamma \partial ^\circ _tf\cdot {\varvec{{g}}}d\sigma -\int _{\Gamma (t)}{\varvec{{g}}}\cdot (\nabla _\Gamma ^T{{\textbf {V}}}_n)\nabla _\Gamma fd\sigma \nonumber \\&\qquad +\int _{\Gamma (t)}\nabla _\Gamma f\cdot {\varvec{{g}}}\ \textrm{div}_\Gamma {{\textbf {V}}}_{\textbf {n}}d\sigma . \end{aligned}$$
- For any ${\textbf {u}},{\textbf {v}}\in H^1_{{\textbf {H}}^1}$ (tangential vector fields) and any function $\eta \in L^\infty _{L^\infty }$ such that $\partial ^\circ _t\eta \in L^\infty _{L^\infty }$, it holds
  2.14 $$\begin{aligned}&\frac{d}{dt}\int _{\Gamma (t)}\eta {\mathcal {E}}_S({\textbf {u}}):{\mathcal {E}}_S({\textbf {v}})d\sigma =\int _{\Gamma }\partial ^\circ _t\eta {\mathcal {E}}_S({\textbf {u}}):{\mathcal {E}}_S({\textbf {v}})d\sigma \nonumber \\&\qquad +\int _\Gamma \eta {\mathcal {E}}_S(\partial ^\circ _t{\textbf {u}}):{\mathcal {E}}_S({\textbf {v}})d\sigma +\int _\Gamma \eta {\mathcal {E}}_S( {\textbf {u}}):{\mathcal {E}}_S(\partial ^\circ _t{\textbf {v}})d\sigma \nonumber \\&\qquad +2\int _{\Gamma }\eta \mathcal {S}(({\textbf {n}}\otimes {\textbf {n}})\widehat{\nabla }_\Gamma {{\textbf {V}}}_{\textbf {n}})\widehat{{\mathcal {E}}}_S({\textbf {u}}):{\mathcal {E}}_S({\textbf {v}})d\sigma +2\int _\Gamma \eta \widehat{{\mathcal {E}}}_S({\textbf {u}})\mathcal {S}(({\textbf {n}}\otimes {\textbf {n}})\widehat{\nabla }_\Gamma {{\textbf {V}}}_{\textbf {n}}):{\mathcal {E}}_S({\textbf {v}})d\sigma \nonumber \\&\qquad +2\int _{\Gamma }\eta {{\mathcal {E}}}_S({\textbf {u}}):\mathcal {S}(({\textbf {n}}\otimes {\textbf {n}})\widehat{\nabla }_\Gamma {{\textbf {V}}}_{\textbf {n}})\widehat{{\mathcal {E}}}_S({\textbf {v}})d\sigma +2\int _{\Gamma }\eta {{\mathcal {E}}}_S({\textbf {u}}):\widehat{{\mathcal {E}}}_S({\textbf {v}})\mathcal {S}(({\textbf {n}}\otimes {\textbf {n}})\widehat{\nabla }_\Gamma {{\textbf {V}}}_{\textbf {n}})d\sigma \nonumber \\&\qquad -\int _\Gamma \eta \mathcal {S}((\nabla _\Gamma {\textbf {u}})\nabla _\Gamma {{\textbf {V}}}_{\textbf {n}}):{\mathcal {E}}_S({\textbf {v}})d\sigma -\int _\Gamma \eta {\mathcal {E}}_S({\textbf {u}}):\mathcal {S}((\nabla _\Gamma {\textbf {v}})\nabla _\Gamma {{\textbf {V}}}_{\textbf {n}})d\sigma \nonumber \\&\qquad +\int _{\Gamma (t)}\eta {\mathcal {E}}_S({\textbf {u}}):{\mathcal {E}}_S({\textbf {v}})\ \textrm{div}_\Gamma {{\textbf {V}}}_{\textbf {n}}d\sigma , \end{aligned}$$
  where $\widehat{{\mathcal {E}}}_{S} (\cdot ):=\frac{\widehat{\nabla }_{\Gamma }\cdot +\widehat{\nabla }_\Gamma ^T\cdot }{2}$ and $\mathcal {S}({\textbf {A}})$ is the symmetric part of a matrix ${\textbf {A}}$. Here by the matrix $(\nabla _\Gamma {\textbf {v}})\nabla _\Gamma {{\textbf {V}}}_{\textbf {n}}$ we denote the matrix product between $\nabla _\Gamma {\textbf {v}}$ and $\nabla _\Gamma {{\textbf {V}}}_{\textbf {n}}$.

#### Remark 2.3

We observe that, by a standard density argument, (2.14) also holds if ${\textbf {u}},{\textbf {v}}\in L^2_{{\textbf {H}}^2}\cap H^1_{{\textbf {L}}^2}$ and $\eta \in L^\infty _{W^{1,\infty }}$ with $\partial ^\circ _t\eta \in L^2_{H^1}$, up to substituting the second line of the right-hand side by

$$ -\int _{\Gamma (t)}\textrm{div}_\Gamma (\eta {\mathcal {E}}_S({\textbf {u}}))\cdot \partial ^\circ _t{\textbf {v}}d\sigma -\int _{\Gamma (t)}\textrm{div}_\Gamma (\eta {\mathcal {E}}_S({\textbf {v}}))\cdot \partial ^\circ _t{\textbf {u}}d\sigma , $$

as it can be easily seen integrating by parts the corresponding terms.

#### Proof

The proofs of (2.11)-(2.12) can be found in [12, Section 8.2], whereas the proof of (2.13) is shown, for instance, in [11, Appendix A.1]. We are left to prove (2.14). To this aim, we first observe that, under the same assumptions of (2.13), it holds, applying (2.11) component-wise,

$$\begin{aligned}&\frac{d}{dt}\int _{\Gamma (t)}\nabla _\Gamma f\cdot {\varvec{{g}}}d\sigma \\&\quad =\int _{\Gamma (t)}\partial ^\circ _t\nabla _\Gamma f\cdot {\varvec{{g}}}d\sigma +\int _{\Gamma (t)}\nabla _\Gamma f\cdot \partial ^\circ _t{\varvec{{g}}}d\sigma +\int _{\Gamma (t)}\nabla _\Gamma f\cdot {\varvec{{g}}}\ \textrm{div}_\Gamma {{\textbf {V}}}_{\textbf {n}}d\sigma , \end{aligned}$$

so that, by comparison with (2.13) and by the arbitrariness of the tangential vectors ${\varvec{{g}}}\in H^1_{{\textbf {L}}^2}$, we obtain

$$\begin{aligned} {\textbf {P}}\partial ^\circ _t\nabla _\Gamma f=\nabla _\Gamma \partial ^\circ _tf-\nabla _\Gamma ^T{{\textbf {V}}}_n\nabla _\Gamma f. \end{aligned}$$

Observe that this identity can be deduced also from [13, Lemma 2.6]. If we now consider a tangential vector field ${\textbf {u}}\in H^1_{{\textbf {H}}^1}$, we then have (by choosing for instance $f={\textbf {u}}_j$, $j=1,2,3$, in the identity above and after some standard computation)

$$\begin{aligned} {\textbf {P}}(\partial ^\circ _t\widehat{\nabla }_{\Gamma } {\textbf {u}}){\textbf {P}}=\nabla _\Gamma \partial ^\circ _t{\textbf {u}}-(\nabla _\Gamma {\textbf {u}})\nabla _\Gamma {{\textbf {V}}}_{\textbf {n}}, \end{aligned}$$

so that, by taking the transpose and summing up dividing by two,

2.15 $$\begin{aligned} {\textbf {P}}(\partial ^\circ _t\widehat{{\mathcal {E}}}_{S} ({\textbf {u}})){\textbf {P}}={\mathcal {E}}_S(\partial ^\circ _t{\textbf {u}})-\mathcal {S}((\nabla _\Gamma {\textbf {u}})\nabla _\Gamma {{\textbf {V}}}_{\textbf {n}}). \end{aligned}$$

We now recall that, as in [18, Lemma 2.2], it holds

$$ \partial ^\circ _t{\textbf {P}}=2\mathcal {S}(({\textbf {n}}\otimes {\textbf {n}})\widehat{\nabla }_\Gamma {{\textbf {V}}}_{\textbf {n}}). $$

By applying (2.11) (if we think component-wise), we get, for ${\textbf {u}},{\textbf {v}}\in H^1_{{\textbf {H}}^1}$ and $\eta \in L^\infty _{L^\infty }$ such that $\partial ^\circ _t\eta \in L^\infty _{L^\infty }$,

2.16 $$\begin{aligned}&\frac{d}{dt}\int _{\Gamma (t)}\eta {\mathcal {E}}_S({\textbf {u}}):{\mathcal {E}}_S({\textbf {v}})d\sigma \nonumber \\&\quad =\int _{\Gamma }\partial ^\circ _t\eta {\mathcal {E}}_S({\textbf {u}}):{\mathcal {E}}_S({\textbf {v}})d\sigma + \int _{\Gamma }\eta \partial ^\circ _t{\mathcal {E}}_S({\textbf {u}}):{\mathcal {E}}_S({\textbf {v}})&\nonumber \\&\qquad +\int _{\Gamma }\eta {\mathcal {E}}_S({\textbf {u}}):\partial ^\circ _t{\mathcal {E}}_S({\textbf {v}})d\sigma +\int _{\Gamma (t)}\eta {\mathcal {E}}_S({\textbf {u}}):{\mathcal {E}}_S({\textbf {v}})\ \textrm{div}_\Gamma {{\textbf {V}}}_{\textbf {n}}d\sigma . \end{aligned}$$

Now, by the product rule for differentiation, we get

$$\begin{aligned} \partial ^\circ _t{\mathcal {E}}_S({\textbf {u}})=\partial ^\circ _t({\textbf {P}}\widehat{{\mathcal {E}}}_S({\textbf {u}}){\textbf {P}})&=(\partial ^\circ _t{\textbf {P}})\widehat{{\mathcal {E}}}_S({\textbf {u}}){\textbf {P}}+{\textbf {P}}\partial ^\circ _t\widehat{{\mathcal {E}}}_S({\textbf {u}}){\textbf {P}}+{\textbf {P}}\widehat{{\mathcal {E}}}_S({\textbf {u}})\partial ^\circ _t{\textbf {P}}\\&=2\mathcal {S}(({\textbf {n}}\otimes {\textbf {n}})\widehat{\nabla }_\Gamma {{\textbf {V}}}_{\textbf {n}})\widehat{{\mathcal {E}}}_S({\textbf {u}}){\textbf {P}}+{\textbf {P}}\partial ^\circ _t\widehat{{\mathcal {E}}}_S({\textbf {u}}){\textbf {P}}\\&\quad +2{\textbf {P}}\widehat{{\mathcal {E}}}_S({\textbf {u}})\mathcal {S}(({\textbf {n}}\otimes {\textbf {n}})\widehat{\nabla }_\Gamma {{\textbf {V}}}_{\textbf {n}}). \end{aligned}$$

For the middle term in the identity above, observe that it holds

$$\begin{aligned} \int _{\Gamma }\eta {\textbf {P}}\partial ^\circ _t\widehat{{\mathcal {E}}}_S({\textbf {u}}){\textbf {P}}:{\mathcal {E}}_S({\textbf {v}})d\sigma =\int _{\Gamma }\eta ({\mathcal {E}}_S(\partial ^\circ _t{\textbf {u}})-\mathcal {S}((\nabla _\Gamma {\textbf {u}})\nabla _\Gamma {{\textbf {V}}}_{\textbf {n}})):{\mathcal {E}}_S({\textbf {v}})d\sigma , \end{aligned}$$

so that, since ${\textbf {P}}{\mathcal {E}}_S({\textbf {v}}){\textbf {P}}={\mathcal {E}}_S({\textbf {v}})$,

$$\begin{aligned} \int _{\Gamma }\eta \partial ^\circ _t{\mathcal {E}}_S({\textbf {u}}):{\mathcal {E}}_S({\textbf {v}})d\sigma&=2\int _{\Gamma }\eta \mathcal {S}(({\textbf {n}}\otimes {\textbf {n}})\widehat{\nabla }_\Gamma {{\textbf {V}}}_{\textbf {n}})\widehat{{\mathcal {E}}}_S({\textbf {u}}):{\mathcal {E}}_S({\textbf {v}})d\sigma \\&\quad +2\int _\Gamma \eta \widehat{{\mathcal {E}}}_S({\textbf {u}})\mathcal {S}(({\textbf {n}}\otimes {\textbf {n}})\widehat{\nabla }_\Gamma {{\textbf {V}}}_{\textbf {n}}):{\mathcal {E}}_S({\textbf {v}})d\sigma \\  &\quad +\int _\Gamma \eta {\mathcal {E}}_S(\partial ^\circ _t{\textbf {u}}):{\mathcal {E}}_S({\textbf {v}})d\sigma -\int _\Gamma \eta \mathcal {S}((\nabla _\Gamma {\textbf {u}})\nabla _\Gamma {{\textbf {V}}}_{\textbf {n}}):{\mathcal {E}}_S({\textbf {v}})d\sigma \end{aligned}$$

Therefore, (2.14) follows from (2.16) inserting the identity above and the one for $\int _{\Gamma }\eta {\mathcal {E}}_S({\textbf {u}}):\partial ^\circ _t{\mathcal {E}}_S({\textbf {v}})d\sigma$, which is identical up to exchanging the roles of ${\textbf {u}}$ and ${\textbf {v}}$. $\square$

### Assumptions on the potential, the viscosity and the density

In the main result of this paper we consider the following assumptions:

1. $\Psi \in C^0([-1,1])\cap C^{4}((-1,1))$. Furthermore, the potential $\Psi :[-1,1]\rightarrow \mathbb {R}$ can be written as
  $$\begin{aligned} \Psi (s)=F(s)-\frac{\theta _0}{2}s^2 \quad \text {for all} s\in [-1,1] \end{aligned}$$
  with a given constant $\theta _0>0$. Moreover $F\in C^0([-1,1])\cap C^{4}((-1,1))$ has the properties
  $$\begin{aligned} \lim _{r\rightarrow -1}F^{\prime }(r)=-\infty , \quad \lim _{r\rightarrow 1}F^{\prime }(r)=+\infty , \quad F^{\prime \prime }(s)\ge {\theta }, \end{aligned}$$
  for all $s\in (-1,1)$ and for some $0<\theta <\theta _0$. Without loss of generality, we further assume $F(0)=0$ and $F'(0)=0$. In particular, this implies that $F(r)\ge 0$ for all $r\in [-1,1]$. We also assume that there exists $\beta >\frac{1}{2}$ such that
  2.17 $$\begin{aligned} \frac{1}{{F^{\prime } (1 - 2\delta )}} = O\left( {\frac{1}{{\left| {\ln \,(\delta )} \right| ^{\beta } }}} \right) ,\quad \frac{1}{{\left| {F^{\prime } ( - 1 + 2\delta )} \right| }} = O\left( {\frac{1}{{\left| {\ln \,(\delta )} \right| ^{\beta } }}} \right) \end{aligned}$$
  as $$\delta \rightarrow 0^+$$.
2. $\nu \in C^{2}(\mathbb {R})$, such that $\nu (s) \ge \nu _* > 0$ for every $s \in \mathbb {R}$ and some $\nu _* > 0$.
3. $\rho (s):=\frac{\widetilde{\rho }_1+\widetilde{\rho }_2}{2}+\frac{\widetilde{\rho }_2-\widetilde{\rho }_1}{2}s,\ \forall s\in \mathbb {R}$, where we set $\rho _*=\min _{s\in [-1,1]}\rho (s)>0$ and $\rho ^*=\max _{s\in [-1,1]}\rho (s)>0$.

#### Remark 2.4

A common form for the viscosity is the following

$$\begin{aligned} \nu (s)=\nu _{1}\frac{1+s}{2}+\nu _{2}\frac{1-s}{2},\quad \forall s\in [-1,1], \end{aligned}$$

which can be easily extended on the whole $\mathbb {R}$ in such way to comply with (H2).

#### Remark 2.5

Notice that the logarithmic potential, which is given by

2.18 $$\begin{aligned} F(s)=\frac{\theta }{2}((1+s)\text {ln}(1+s)+(1-s)\text {ln}(1-s)) \quad \text {for all} s\in (-1,1), \end{aligned}$$

complies with assumption (H1), with some $0<\theta <\theta _0$. Nevertheless, much more general singular potentials are allowed by (2.17) (see, for instance, [16]).

## Main result

In this section we present the main result of this paper, i.e., the existence and uniqueness of a global strong solution to (1.1). We first give the definition of a strong solution on an interval $[0,T_0]$, with $0<T_0\le T$, to problem (1.1), namely

### Definition 3.1

(Strong solution) Let us set ${\textbf {v}}_0 \in {\textbf {H}}^1(\Gamma _0)$ and $\varphi _0\in {H}^2 (\Gamma _0)$ such that $-\Delta \varphi _0+\Psi '(\varphi _0)=:\mu _0\in H^1(\Gamma _0)$, $\left\| \varphi _0\right\| _{L^\infty (\Gamma _0)}\le 1$ and $\left| \overline{\varphi }_0\right| =\left| \frac{\int _{\Gamma _0}\varphi _0d\sigma }{\left| \Gamma _0\right| }\right| <1$. We call $({\textbf {v}},p,\varphi )$ a strong solution to (1.1) on $[0,T_0]$ if

$$\begin{aligned}&{\textbf {v}} \in L ^2_{{\textbf {H}}^2(T_0)}\cap H^1_{{\textbf {L}}^2(T_0)} ,\quad p\in L^2_{H^1(T_0)},\quad \varphi \in L^\infty _{W^{2,q}(T_0)} \cap H^1_{{H}^1(T_0)} ,\quad \forall q\in [2,+\infty ),\\  &\left| \varphi \right| <1\quad \text {a.e. in }\Gamma (t) \quad \text {for a.a. }t\in [0,T_0],\quad \mu \in {L}^\infty _{H^1(T_0)}\cap L^2_{H^3(T_0)}, \end{aligned}$$

and it satisfies (1.1) in the almost everywhere sense.

### Remark 3.2

Since, by Lemma 2.1, $L ^2_{{\textbf {H}}^2}\cap H^1_{{\textbf {L}}^2}\hookrightarrow C_{{\textbf {H}}^1}$, and $H^1_{H^1}\hookrightarrow C_{H^1}$, the initial conditions ${\textbf {v}}(0)={\textbf {v}}_0$ and $\varphi (0)=\varphi _0$ make sense.

Our main result then reads

### Theorem 3.3

(Global well-posedness of strong solutions) Assume the regularity assumptions on $\Phi _t^n$ stated in Sect. 2.1, and assumptions (H1)-(H3) for $\Psi$, $\nu$, and $\rho$. Let ${\textbf {v}}_0 \in {\textbf {H}}^1(\Gamma _0)$ and $\varphi _0\in {H}^2 (\Gamma _0)$, be such that $-\Delta _{\Gamma _0}\varphi _0+\Psi '(\varphi _0)=:\mu _0\in H^1(\Gamma _0)$, $\left\| \varphi _0\right\| _{L^\infty (\Gamma _0)}\le 1$ and $\left| \overline{\varphi }_0\right| <1$. Then there exists a unique global strong solution $({\textbf {v}},p, \varphi )$ to (1.1) on [0, *T*] in the sense of Definition 3.1. Furthermore, the following additional regularity holds: there exists $\delta >0$ depending only on the parameters of the problem, *F*, *T*, the initial energy $E_{tot}(\rho _0,{\textbf {v}}_0,\varphi _0)$, and $\left\| \mu _0\right\| _{H^1(\Gamma _0)}$, such that

3.1 $$\begin{aligned} \sup _{t\in [0,T]} \left\| \varphi \right\| _{C(\Gamma (t))}\le 1-\delta . \end{aligned}$$

This entails

3.2 $$\begin{aligned} \varphi \in L^\infty _{H^3}\cap L^2_{H^4},\quad \mu \in H^1_{H^{-1}}. \end{aligned}$$

### Remark 3.4

We observe that, exploiting the estimates obtained in the first part of the proof of Theorem 3.3 (see, in particular, the energy estimate (5.14) below), one can also prove the existence of global-in-time weak solutions to the same problem (1.1), under weaker assumptions on the regularity of the intial data. The same result has been obtained, in the case of matched densities (i.e., $\rho =const$) in [14]. Using our approach, the existence result of weak solutions can also be extended to the more general case of unmatched densities (we also refer to [1, 3] for a definition of weak solutions in the unmatched densities case in bounded domains). We leave the details to the interested reader.

Before proving this result, in the next section we analyze the well-posedness of a convective Cahn-Hilliard equation on evolving surfaces. This is a milestone to prove our main result.

## Regularity results for the advective Cahn-Hilliard equation on evolving surfaces

In this section we address the well-posedness of the following problem:

4.1 $$\begin{aligned} {\left\{ \begin{array}{ll} \partial ^\circ _t\varphi -\Delta _{\Gamma }\mu +\nabla _\Gamma \varphi \cdot {\textbf {v}}=0,& \quad \text { on }\Gamma ,\\ \mu =-\Delta _\Gamma \varphi +\Psi '(\varphi ),& \quad \text { on }\Gamma ,\\ \varphi (0)=\varphi _0,& \quad \text { on }\Gamma _0,\end{array}\right. } \end{aligned}$$

where ${\textbf {v}}={\textbf {u}}+{{\textbf {V}}}_{\textbf {n}}$ is divergence-free, and ${\textbf {u}}$ is a tangent vector. In particular, it holds the following

### Theorem 4.1

Let $\Psi$ satisfy assumption (H1) and the flow map satisfy the assumptions of Sect. 2.1, and let $\varphi _0\in H^2(\Gamma _0)$ such that $\left| \overline{\varphi }_0\right| <1$, $\left\| \varphi _0\right\| _{L^\infty (\Gamma _0)}\le 1$, and $\mu _0:=-\Delta _{\Gamma _0}\varphi _0+\Psi '(\varphi _0)\in H^1(\Gamma _0)$. Assume also that ${\textbf {v}}={\textbf {u}}+{{\textbf {V}}}_{\textbf {n}}$ and ${\textbf {u}}\in L^2_{{\textbf {H}}^1(\widetilde{T})}$, for some $\widetilde{T}\in (0,T]$, and $\textrm{div}_\Gamma {\textbf {v}}\equiv 0$. Then there exists a unique pair $(\varphi ,\mu )$ with

$$\begin{aligned}&\varphi \in L^\infty _{H^3(\widetilde{T})}\cap L^2_{H^4(\widetilde{T})}\cap H^1_{H^{1}(\widetilde{T})},\\  &\mu \in L^\infty _{H^1(\widetilde{T})}\cap L^2_{H^3(\widetilde{T})}\cap H^1_{H^{-1}(\widetilde{T})},\\  &F'(\varphi )\in L^\infty _{L^{p}(\widetilde{T})},\quad \forall p\ge 2 \end{aligned}$$

such that it holds

$$ \sup _{t\in [0,\widetilde{T}]}\left\| \varphi (t)\right\| _{C(\Gamma (t))}\le 1-\delta , $$

for some $\delta =\delta (T,\left\| {\textbf {u}}\right\| _{L^2_{{\textbf {H}}^1(\widetilde{T}) }})\in (0,1)$, continuously decreasing towards 0 with the increase of the $L^2_{{\textbf {H}}^1(\widetilde{T})}$ norm of ${\textbf {u}}$. The pair $(\varphi ,\mu )$ is a strong solution to problem (4.1), i.e., it satisfies the equation in the almost everywhere sense (for almost any $t\in [0,T]$ almost everywhere on $\Gamma (t)$). In particular, there exists $C(T,\left\| {\textbf {u}}\right\| _{L^2_{{\textbf {H}}^1(\widetilde{T}) }})>0$, continuously increasing with the $L^2_{{\textbf {H}}^1(\widetilde{T})}$ norm of ${\textbf {u}}$, such that

4.2 $$\begin{aligned} \left\| \mu \right\| _{L^\infty _{H^1(\widetilde{T})}}&+\left\| \mu \right\| _{L^2_{H^3(\widetilde{T})}}+\left\| \mu \right\| _{H^1_{H^{-1}(\widetilde{T})}}+\left\| \varphi \right\| _{L^\infty _{H^3(\widetilde{T})}}+\left\| \varphi \right\| _{H^1_{H^1(\widetilde{T})}}\nonumber \\&\le C(T,\left\| {\textbf {u}}\right\| _{L^2_{{\textbf {H}}^1(\widetilde{T}) }}). \end{aligned}$$

### Remark 4.2

Note that, since $\varphi \in H^1_{H^1(\widetilde{T})}\hookrightarrow C_{H^1(\widetilde{T})}$, the initial condition $\varphi (0)=\varphi _0$ makes sense.

### Remark 4.3

Due to the assumption $\mu _0\in H^1(\Gamma _0)$, there exists $\delta _0>0$ such that

$$ \left\| \varphi _0\right\| _{C^0(\Gamma _0)}\le 1-\delta _0. $$

Indeed, it is immediate to see that we have, thanks to (2.8),

$$ \left\| \mu _0\right\| _{L^p(\Gamma _0)}+\left\| F'(\varphi _0)\right\| _{L^p(\Gamma _0)}\le C\sqrt{p}, \quad \forall p\ge 2. $$

Then we can repeat the proof of [16, Theorem 3.3] for the time $t=0$ and deduce the result (see also the proof of (4.33) below for a similar argument). Therefore, since $\mu _0\in H^1(\Gamma _0)$ and, exploiting the separation property above, $\Psi '(\varphi _0)\in H^1(\Gamma _0)$, it is immediate to deduce by elliptic regularity that $\varphi _0\in H^3(\Gamma _0)$.

### Proof

**Uniqueness.** First of all, uniqueness also holds for weak solutions and the proof is an immediate adaptation, for instance, of [10]. Let us introduce the inverse Laplacian operator $\mathcal {N}(t)$ such that, for any $f\in H^{-1}(\Gamma (t))$ with $<f,1>_{H^{-1},H^1}=0$, it holds (recall that $\mathcal {N}(t)f\in H^1(\Gamma (t))$)

$$\begin{aligned} \int _{\Gamma (t)}\nabla _\Gamma \mathcal {N}(t)f\cdot \nabla _\Gamma \eta d\sigma =<f,\eta >_{H^{-1},H^1},\quad \forall \eta \in H^1(\Gamma (t)). \end{aligned}$$

This operator defines a norm on $H^{-1}(\Gamma (t))$ with zero mean (see for instance, [10, Appendix C]) which we denote as $\left\| f\right\| _{\sharp }=\left\| \nabla \mathcal {N}(t)f\right\|$. Clearly this norm changes with $t\in [0,T]$. Furthermore, it holds, by standard elliptic regularity (see, e.g., (2.10)),

4.3 $$\begin{aligned} \left\| \mathcal {N}(t)f\right\| _{H^2(\Gamma (t))}\le C(T)\left\| f\right\| , \quad \forall f\in L^2(\Gamma (t))\quad \text {s.t. }\overline{f}=0. \end{aligned}$$

We then consider two initial data $\varphi _{0,1}$ and $\varphi _{0,2}$ satisfying the assumptions of the theorem and such that $\overline{\varphi }_{0,1}=\overline{\varphi }_{0,2}$. Let us define as $(\varphi _i,\mu _i)$, $i=1,2$, the corresponding solutions. By conservation of total mass, this means that $\overline{\varphi }_1\equiv \overline{\varphi }_2$. Let us then consider the variables $\varphi :=\varphi _1-\varphi _2$ and $\mu :=\mu _1-\mu _2$. They satisfy

4.4 $$\begin{aligned} \partial ^\circ _t\varphi +{\textbf {v}}\cdot \nabla _\Gamma \varphi -\Delta _\Gamma \mu =0. \end{aligned}$$

Now we recall that, by (2.11),

$$\begin{aligned} \frac{1}{2} \frac{d}{dt}\left\| \nabla _\Gamma \mathcal {N}(t)\varphi \right\| ^2&=\int _{\Gamma (t)}\nabla _\Gamma \mathcal {N}(t)\varphi \cdot \nabla _\Gamma \partial ^\circ _t\mathcal {N}(t)\varphi d\sigma +\frac{1}{2}\int _{\Gamma (t)}\left| \nabla _\Gamma \mathcal {N}(t)\varphi \right| ^2 Hv_{\textbf {n}}d\sigma \\  &\quad -\frac{1}{2}\int _{\Gamma (t)}\nabla _\Gamma \mathcal {N}(t)\varphi \cdot {\mathcal {E}}_S({{\textbf {V}}}_{\textbf {n}})\nabla _\Gamma \mathcal {N}(t)\varphi d\sigma \\  &=\int _{\Gamma (t)}\varphi \partial ^\circ _t\mathcal {N}(t)\varphi d\sigma +\frac{1}{2}\int _{\Gamma (t)}\left| \nabla _\Gamma \mathcal {N}(t)\varphi \right| ^2 Hv_{\textbf {n}}d\sigma \\  &\quad -\frac{1}{2}\int _{\Gamma (t)}\nabla _\Gamma \mathcal {N}(t)\varphi \cdot {\mathcal {E}}_S({{\textbf {V}}}_{\textbf {n}})\nabla _\Gamma \mathcal {N}(t)\varphi d\sigma \\  &=\frac{d}{dt}\int _{\Gamma (t)}\varphi \mathcal {N}(t)\varphi d\sigma -\int _{\Gamma (t)}\partial ^\circ _t\varphi {\mathcal {N}}(t)\varphi d\sigma -\int _{\Gamma (t)}\varphi \mathcal {N}(t)\varphi Hv_{\textbf {n}}d\sigma \\  &\quad {+}\frac{1}{2}\int _{\Gamma (t)}\left| \nabla _\Gamma \mathcal {N}(t)\varphi \right| ^2 Hv_{\textbf {n}}d\sigma {-}\frac{1}{2}\int _{\Gamma (t)}\nabla _\Gamma \mathcal {N}(t)\varphi \cdot {\mathcal {E}}_S({{\textbf {V}}}_{\textbf {n}})\nabla _\Gamma \mathcal {N}(t)\varphi d\sigma , \end{aligned}$$

so that, since $\left\| \varphi \right\| _\sharp ^2=\int _{\Gamma (t)}\varphi \mathcal {N}(t)\varphi d\sigma =\left\| \nabla _\Gamma \mathcal {N}(t)\varphi \right\| ^2$, we infer

$$\begin{aligned}&\frac{1}{2} \frac{d}{dt}\left\| \varphi \right\| _\sharp ^2=\int _{\Gamma (t)}\partial ^\circ _t\varphi {\mathcal {N}}(t)\varphi d\sigma +\int _{\Gamma (t)}\varphi \mathcal {N}(t)\varphi Hv_{\textbf {n}}d\sigma \\  &\quad -\frac{1}{2}\int _{\Gamma (t)}\left| \nabla _\Gamma \mathcal {N}(t)\varphi \right| ^2 Hv_{\textbf {n}}d\sigma +\frac{1}{2}\int _{\Gamma (t)}\nabla _\Gamma \mathcal {N}(t)\varphi \cdot {\mathcal {E}}_S({{\textbf {V}}}_{\textbf {n}})\nabla _\Gamma \mathcal {N}(t)\varphi d\sigma \end{aligned}$$

By testing equation (4.4) with $\eta =\mathcal {N}(t)\varphi$, we then deduce

4.5 $$\begin{aligned}&\frac{1}{2} \frac{d}{dt}\left\| \varphi \right\| _\sharp ^2+(\nabla _\Gamma \mu ,\nabla _\Gamma \mathcal {N}(t)\varphi )+({\textbf {v}}\cdot \nabla _\Gamma \varphi ,\mathcal {N}(t)\varphi )\nonumber \\&\quad =\int _{\Gamma (t)}\varphi \mathcal {N}(t)\varphi Hv_{\textbf {n}}d\sigma -\frac{1}{2}\int _{\Gamma (t)}\left| \nabla _\Gamma \mathcal {N}(t)\varphi \right| ^2 Hv_{\textbf {n}}d\sigma \nonumber \\&\qquad +\frac{1}{2}\int _{\Gamma (t)}\nabla _\Gamma \mathcal {N}(t)\varphi \cdot {\mathcal {E}}_S({{\textbf {V}}}_{\textbf {n}})\nabla _\Gamma \mathcal {N}(t)\varphi d\sigma . \end{aligned}$$

First, we observe that

$$\begin{aligned} (\nabla _\Gamma \mu ,\nabla _\Gamma \mathcal {N}(t)\varphi )=(\mu ,\varphi )=\int _{\Gamma (t)}(F'(\varphi _1)-F'(\varphi _2))\varphi -\theta _0\left\| \varphi \right\| ^2+\left\| \nabla _\Gamma \varphi \right\| ^2d\sigma , \end{aligned}$$

and note that

$$ \int _{\Gamma (t)}(F'(\varphi _1)-F'(\varphi _2))\varphi d\sigma \ge \theta \left\| \varphi \right\| ^2. $$

Furthermore, by Cauchy-Schwarz and Young’s inequalities,

4.6 $$\begin{aligned} \theta _0\left\| \varphi \right\| ^2=\theta _0\int _{\Gamma (t)}\nabla _\Gamma \mathcal {N}(t)\varphi \cdot \nabla _\Gamma \varphi d\sigma \le C\left\| \varphi \right\| _\sharp ^2+\frac{1}{4}\left\| \nabla _\Gamma \varphi \right\| ^2. \end{aligned}$$

Then, concerning the advective term, by Hölder’s, Young’s and Sobolev-Gagliardo-Nirenberg’s inequalities (see also (2.8)), after integrating by parts, applying Poincaré’s inequality (recall that $\overline{{\mathcal {N}}\varphi }\equiv 0$) and recalling ${\textbf {v}}={{\textbf {V}}}_{\textbf {n}}+{\textbf {u}}$, (4.3), and the same interpolation as in (4.6),

$$\begin{aligned} \left| ({\textbf {v}}\cdot \nabla _\Gamma \varphi ,\mathcal {N}(t)\varphi )\right|&\le \left| ({\textbf {v}}\varphi ,\nabla _\Gamma \mathcal {N}(t)\varphi )\right| +\left| \int _{\Gamma (t)}\varphi \mathcal {N}(t)\varphi Hv_{\textbf {n}}d\sigma \right| \\  &\le C(1+\left\| {\textbf {u}}\right\| )\left\| \varphi \right\| _{L^4(\Gamma (t))}\left\| \nabla _\Gamma \mathcal {N}(t)\varphi \right\| _{{\textbf {L}}^4(\Gamma (t))}+C\left\| \varphi \right\| \left\| \nabla _\Gamma \mathcal {N}\varphi \right\| \\  &\le C(1+\left\| {\textbf {u}}\right\| )\left\| \varphi \right\| ^\frac{1}{2}\left\| \nabla _\Gamma \varphi \right\| ^\frac{1}{2}\left\| \varphi \right\| _{\sharp }^\frac{1}{2}\left\| \varphi \right\| ^\frac{1}{2}+C\left\| \varphi \right\| \left\| \varphi \right\| _{\sharp }\\  &\le C(1+\left\| {\textbf {u}}\right\| )\left\| \nabla _\Gamma \varphi \right\| \left\| \varphi \right\| _{\sharp }+C\left\| \nabla _\Gamma \varphi \right\| ^\frac{1}{2}\left\| \varphi \right\| _{\sharp }^\frac{3}{2}\\  &\le \frac{1}{4} \left\| \nabla _\Gamma \varphi \right\| ^2+C(1+\left\| {\textbf {u}}\right\| ^2)\left\| \varphi \right\| ^2_\sharp . \end{aligned}$$

To conclude, we have, by the regularity of the flow map,

$$\begin{aligned}&\left| \int _{\Gamma (t)}\varphi \mathcal {N}(t)\varphi Hv_{\textbf {n}}d\sigma -\frac{1}{2}\int _{\Gamma (t)}\left| \nabla _\Gamma \mathcal {N}(t)\varphi \right| ^2 Hv_{\textbf {n}}d\sigma +\frac{1}{2}\int _{\Gamma (t)}\nabla _\Gamma \mathcal {N}(t)\varphi \cdot {\mathcal {E}}_S({{\textbf {V}}}_{\textbf {n}})\nabla _\Gamma \mathcal {N}(t)\varphi \right| d\sigma \\  &\quad \le C(\left\| \varphi \right\| \left\| \mathcal {N}(t)\varphi \right\| +\left\| \varphi \right\| _\sharp ^2) \le C(\left\| \nabla _\Gamma \varphi \right\| ^\frac{1}{2}\left\| \varphi \right\| _{\sharp }^\frac{3}{2}+\left\| \varphi \right\| _\sharp ^2)\\  &\quad \le \frac{1}{4} \left\| \nabla _\Gamma \varphi \right\| ^2+C\left\| \varphi \right\| _{\sharp }^2. \end{aligned}$$

To sum up, putting all these estimates in (4.5), we get

4.7 $$\begin{aligned} \frac{d}{dt}\left\| \varphi \right\| _\sharp ^2+\frac{1}{4}\left\| \nabla _\Gamma \varphi \right\| ^2+\theta _0\left\| \varphi \right\| ^2\le C(T)(1+\left\| {\textbf {u}}\right\| ^2)\left\| \varphi \right\| _\sharp ^2,\quad \forall t\in [0,\widetilde{T}], \end{aligned}$$

so that uniqueness follows by an application of the Gronwall lemma, recalling that ${\textbf {u}}\in L^2_{{\textbf {H}}^1(\widetilde{T})}$.

### Remark 4.4

We point out that uniqueness holds, with the same proof, for weak solutions. Furthermore, to ensure the sole uniqueness the regularity ${\textbf {u}}\in L^2_{{\textbf {L}}^2(\widetilde{T})}$ is enough.

**Existence.** Very similar results have been obtained also in [11, Theorem 3.9] under even more general assumptions on the normal evolution velocity ${{\textbf {V}}}_{\textbf {n}}$ of the surface. Here the main difference is related to the advective term ${\textbf {u}}$, corresponding to set ${{\textbf {V}}}_\tau ={{\textbf {V}}}_a={\textbf {u}}$ in [11], since in this case it is much less regular, compared to the assumptions on the advective terms ${\textbf {V}}_a$ and ${{\textbf {V}}}_\tau$ (see also assumption [11, (2.4)]). Nevertheless, the approximation procedure by means of a suitable Galerkin scheme and an approximation of the singular potential $\Psi$ can be borrowed identically also in this case. In particular, since ${{\textbf {V}}}_{\textbf {n}}$ is already very regular due to the flow map, we can approximate the velocity ${\textbf {u}}$ with a sequence $\{{\textbf {u}}_k\}_k\subset {C}^\infty _{{\textbf {H}}^1\cap {\textbf {L}}^2}$. Another possibility is to show the existence of a local regular solution in a way similar to what is done in [4, Theorem 4.1] and use that solutions for the computations.

In any case, for the sake of brevity, here we only present the formal necessary estimates, and we refer to the proof of [11, Theorem 3.9] for the more technical details on a possible rigorous approximating scheme.

Let us then operate formally, assuming to have a sufficiently regular solution. Since in general in the approximation schemes we do not have the bound $\left| \varphi \right| <1$, we do not assume this and will deduce it as a consequence of the estimates. Now, first, we observe that the total mass is conserved. Indeed, integrating by parts, recalling (2.11),

$$ \frac{d}{dt}\int _{\Gamma (t)}\varphi d\sigma =\int _{\Gamma (t)}\partial ^\circ _t\varphi d\sigma +\int _{\Gamma (t)}\varphi \textrm{div}_\Gamma {{\textbf {V}}}_{\textbf {n}}d\sigma =\int _{\Gamma (t)}\partial ^\circ _t\varphi d\sigma +\int _{\Gamma (t)}\varphi H{\textbf {v}}_nd\sigma , $$

and, thus by integrating over $\Gamma (t)$ equation (4.1)$_1$, we get, integrating by parts and recalling $\textrm{div}_\Gamma {\textbf {v}}=0$ and ${\textbf {v}}={\textbf {u}}+{{\textbf {V}}}_{\textbf {n}}$,

$$\begin{aligned} 0&=\int _{\Gamma (t)}\partial ^\circ _t\varphi d\sigma -\int _{\Gamma (t)}\varphi \textrm{div}_\Gamma {\textbf {v}}d\sigma +\int _{\Gamma (t)}\varphi H{\textbf {v}}\cdot {\textbf {n}}d\sigma =\int _{\Gamma (t)}\partial ^\circ _t\varphi d\sigma +\int _{\Gamma (t)}\varphi H{\textbf {v}}_nd\sigma \\  &\quad =\frac{d}{dt}\int _{\Gamma (t)}\varphi d\sigma , \end{aligned}$$

entailing the desired mass conservation

4.8 $$\begin{aligned} \overline{\varphi }(t)=\frac{ \int _{\Gamma (t)}\varphi d\sigma }{\left| \Gamma (t)\right| }=\frac{ \int _{\Gamma (t)}\varphi d\sigma }{\left| \Gamma _0\right| }\equiv \frac{ \int _{\Gamma _0} \varphi _0d\sigma }{\left| \Gamma _0\right| },\quad \forall t\in [0,T], \end{aligned}$$

where we have used the surface inextensibiliy assumption, so that $\left| \Gamma (t)\right| =\left| \Gamma _0\right|$ for any $t\in [0,T]$.

Now, recalling (2.11) and (2.12), we have, since $\textrm{div}_\Gamma {{\textbf {V}}}_{\textbf {n}}=H v_{\textbf {n}}$,

$$\begin{aligned} \frac{1}{2}\frac{d}{dt}\Vert \nabla _\Gamma \varphi \Vert ^2&=\int _{\Gamma (t)}\nabla _\Gamma \varphi \cdot \nabla _\Gamma \partial ^\circ _t\varphi d\sigma +\frac{1}{2}\int _{\Gamma (t)}\vert \nabla _\Gamma \varphi \vert ^2H v_{\textbf {n}}d\sigma \\  &\quad -\int _{\Gamma (t)}\nabla _\Gamma \varphi \cdot {\mathcal {E}}_S({{\textbf {V}}}_{\textbf {n}})\nabla _\Gamma \varphi d\sigma , \end{aligned}$$

as well as

$$\begin{aligned} \frac{d}{dt} \int _{\Gamma (t)}\Psi (\varphi )d\sigma =\int _{\Gamma (t)}\Psi '(\varphi )\partial ^\circ _t\varphi d\sigma +\int _{\Gamma (t)}\Psi (\varphi )H v_{\textbf {n}}d\sigma . \end{aligned}$$

Therefore, we obtain a standard energy estimate by multiplying (4.1)$_1$ by $\mu$: since, integrating by parts,

$$ \int _{\Gamma (t)}\partial ^\circ _t\varphi \mu d\sigma =\int _{\Gamma (t)}\nabla _\Gamma \varphi \cdot \nabla _\Gamma \partial ^\circ _t\varphi d\sigma +\int _{\Gamma (t)}\Psi '(\varphi )\partial ^\circ _t\varphi d\sigma , $$

we infer

4.9 $$\begin{aligned}&\frac{d}{dt}\left( \frac{1}{2}\Vert \nabla _\Gamma \varphi \Vert ^2+\int _{\Gamma (t)}\Psi (\varphi )\right) d\sigma +\int _{\Gamma (t)}\vert \nabla _\Gamma \mu \vert ^2d\sigma +\int _{\Gamma (t)}\nabla _\Gamma \varphi \cdot {\textbf {v}}\ \mu d\sigma \nonumber \\&\quad =\frac{1}{2}\int _{\Gamma (t)}\vert \nabla _\Gamma \varphi \vert ^2H v_{\textbf {n}}d\sigma -\int _{\Gamma (t)}\nabla _\Gamma \varphi \cdot {\mathcal {E}}_S({{\textbf {V}}}_{\textbf {n}})\nabla _\Gamma \varphi d\sigma +\int _{\Gamma (t)}\Psi (\varphi )H v_{\textbf {n}}d\sigma . \end{aligned}$$

Moreover, by multiplying equation (4.1)$_1$ by $\varphi$, we deduce, again using (2.11) and integrating by parts,

$$\begin{aligned} \frac{1}{2}\frac{d}{dt}\left\| \varphi \right\| ^2+\left\| \Delta _\Gamma \varphi \right\| ^2+\int _{\Gamma (t)}\Psi ''(\varphi )\vert \nabla _\Gamma \varphi \vert ^2d\sigma +\int _{\Gamma (t)}\vert \varphi \vert ^2 H v_{\textbf {n}}d\sigma =0, \end{aligned}$$

where we used the fact that $\textrm{div}_\Gamma {\textbf {v}}=0$. This entails, by the regularity of the flow map, that

$$ \frac{d}{dt}\left\| \varphi \right\| ^2+\left\| \Delta _\Gamma \varphi \right\| ^2+\int _{\Gamma (t)}F''(\varphi )\vert \nabla _\Gamma \varphi \vert ^2d\sigma \le C\left\| \varphi \right\| ^2+\theta _0\left\| \nabla \varphi \right\| ^2, $$

but the standard interpolation

$$ \theta _0\left\| \nabla _\Gamma \varphi \right\| ^2=-\theta _0\int _{\Gamma (t)}\varphi \Delta _{\Gamma }\varphi d\sigma \le \theta _0\left\| \varphi \right\| \left\| \Delta _{\Gamma }\varphi \right\| $$

entails by Young’s inequality that

$$ \frac{d}{dt}\left\| \varphi \right\| ^2+\frac{1}{2}\left\| \Delta _\Gamma \varphi \right\| ^2+\int _{\Gamma (t)}F''(\varphi )\vert \nabla _\Gamma \varphi \vert ^2d\sigma \le C\left\| \varphi \right\| ^2,\quad \forall t\in [0,\widetilde{T}] $$

so that, by Gronwall’s Lemma we deduce

4.10 $$\begin{aligned} \varphi \in L^\infty _{L^2(\widetilde{T})}. \end{aligned}$$

Now we can estimate all the terms appearing in the identity (4.9). First, integrating by parts, by Poincaré’s and Sobolev-Gagliardo-Nirenberg’s inequalities, recalling the regularity of ${{\textbf {V}}}_{\textbf {n}}$, (4.8) and (4.10), we deduce that

$$\begin{aligned} \left| \int _{\Gamma (t)}\nabla _\Gamma \varphi \cdot {\textbf {v}}\ \mu d\sigma \right|&\le \left| \int _{\Gamma (t)}\varphi {\textbf {v}}\cdot \nabla _\Gamma \mu d\sigma \right| +\left| \int _{\Gamma (t)}\varphi \mu Hv_{\textbf {n}}d\sigma \right| \\&\le \left\| \varphi \right\| _{L^4(\Gamma (t))}(1+\left\| {\textbf {u}}\right\| _{{\textbf {L}}^4(\Gamma (t))})\left\| \nabla _\Gamma \mu \right\| +C\left\| \varphi \right\| \left\| \mu \right\| \\&\le \left\| \varphi \right\| ^\frac{1}{2}\left\| \varphi \right\| _{H^1(\Gamma (t))}^\frac{1}{2}(1+\left\| {\textbf {u}}\right\| _{{\textbf {L}}^4(\Gamma (t))})\left\| \nabla _\Gamma \mu \right\| +C\left\| \mu \right\| \\&\le C(1+\left\| \nabla _\Gamma \varphi \right\| ^\frac{1}{2})(1+\left\| {\textbf {u}}\right\| _{{\textbf {L}}^4(\Gamma (t))})\left\| \nabla _\Gamma \mu \right\| +C(\left\| \mu -\overline{\mu }\right\| +\left| \overline{\mu }\right| )\\&\le C_\epsilon (1+\left\| {\textbf {u}}\right\| _{{\textbf {L}}^4(\Gamma (t))}^2)(1+\left\| \nabla _\Gamma \varphi \right\| ^2)+\frac{1}{2} \left\| \nabla _\Gamma \mu \right\| ^2+\epsilon \left| \overline{\mu }\right| ^2, \end{aligned}$$

for some $\epsilon >0$ to be chosen later on. Concerning the other terms, we easily get, by the regularity of the flow map,

$$\begin{aligned}&\left| \frac{1}{2}\int _{\Gamma (t)}\vert \nabla _\Gamma \varphi \vert ^2H v_{\textbf {n}}d\sigma -\int _{\Gamma (t)}\nabla _\Gamma \varphi \cdot {\mathcal {E}}_S({{\textbf {V}}}_{\textbf {n}})\nabla _\Gamma \varphi d\sigma +\int _{\Gamma (t)}\Psi (\varphi )H v_{\textbf {n}}\right| d\sigma \\&\quad \le C\left( \frac{1}{2}\Vert \nabla _\Gamma \varphi \Vert ^2+\int _{\Gamma (t)}(\Psi (\varphi )+\tilde{C})d\sigma \right) +C, \end{aligned}$$

where $\tilde{C}>0$ is a suitable constant such that $\Psi \ge -\tilde{C}$ (recall that $\Psi$ is bounded below by assumption, and can be chosen as such also in a suitable approximation). We are then left with the estimation of $\overline{\mu }$, which can be performed similarly to [10, 11]. In particular, thanks to mass conservation, it holds

4.11 $$\begin{aligned} \Vert F^{\prime }(\varphi )\Vert _{L^{1}(\Gamma (t) )}\le C_1\left| \int _{\Gamma (t)}F^{\prime }(\varphi )\big ( \varphi -\overline{\varphi }\big )d\sigma \right| +C_{2}, \end{aligned}$$

where $C_1$ and $C_2$ are positive constants only depending on $F'$, $\left| \Gamma _0\right|$ and $\overline{\varphi }_{0}$. We sketch the argument (devised, for instance, in [19]) to show that the constants are not depending on other quantities. Since by assumption it holds $\left| \overline{\varphi }\right| \in (0,1)$, and since $F'(s)\rightarrow \pm \infty$ as $s\rightarrow \pm 1$ and $F'$ is monotone increasing, there exists $m\in [0,1)$ such that $F'(s)>0$ for any $s\in [m,1)$, $F'(s)<0$ for any $s\in (-1,-m]$ and $\left| \overline{\varphi }_0\right| <m$. Then we set

$$\begin{aligned} \Gamma _1(t)&:=\{x\in \Gamma (t):\ -m\le \varphi (x,t)\le m\},\quad \Gamma _2(t):=\{x\in \Gamma (t):\ \varphi (x,t) \\  &\quad < -m \text { or }\varphi (x,t)>m\}, \end{aligned}$$

and define $\delta _1:=\min \{\overline{\varphi }_0+m,m-\overline{\varphi }_0\}>0$. Then it holds, recalling that $\left| \Gamma (t)\right| =\left| \Gamma _0\right|$ and $\overline{\varphi }=\overline{\varphi }_0$,

$$\begin{aligned} \delta _1\left\| F'(\varphi )\right\| _{L^1(\Gamma (t))}&= \delta _1\int _{{\Gamma _1}(t)}\left| F'(\varphi )\right| d\sigma +\delta _1\int _{{\Gamma _2}(t)}\left| F'(\varphi )\right| d\sigma \\  &\le \delta _1\max _{s\in [-m,m]}\left| F'(s)\right| \left| \Gamma (t)\right| +\int _{\Gamma _2(t)}{F'(\varphi )}(\varphi -\overline{\varphi }_0)d\sigma \\  &\le \delta _1\max _{s\in [-m,m]}\left| F'(s)\right| \left| \Gamma (t)\right| +\int _{\Gamma (t)}{F'(\varphi )}(\varphi -\overline{\varphi }_0)d\sigma \\  &\quad -\int _{\Gamma _1(t)}{F'(\varphi )}(\varphi -\overline{\varphi }_0)d\sigma \\  &\le 2\delta _1\max _{s\in [-m,m]}\left| F'(s)\right| \left| \Gamma _0\right| +\left| \int _{\Gamma (t)}{F'(\varphi )}(\varphi -\overline{\varphi })d\sigma \right| , \end{aligned}$$

where in the last inequality we used the fact that

$$ -\int _{\Gamma _1(t)}{F'(\varphi )}(\varphi -\overline{\varphi }_0)d\sigma \le \delta _1\max _{s\in [-m,m]}\left| F'(s)\right| \left| \Gamma _0\right| . $$

Therefore, it is easy to identify $C_1:=\frac{1}{\delta _1}$ and $C_2:=2\max _{s\in [-m,m]}\left| F'(s)\right| \left| \Gamma _0\right|$ and these constants only depend on $\overline{\varphi }_0, F', \left| \Gamma _0\right|$ as expected. Now, from the definition of $\mu$, integrating by parts, recalling (4.10), we get

4.12 $$\begin{aligned} \left\| \nabla _\Gamma \varphi \right\| ^2+\int _{\Gamma (t)}{F'(\varphi )}(\varphi -\overline{\varphi })d\sigma&\le \left\| \mu -\overline{\mu }\right\| \left\| \varphi -\overline{\varphi }\right\| +\theta _0\left\| \varphi \right\| \left\| \varphi -\overline{\varphi }\right\| \nonumber \\&\le C(1+\left\| \mu -\overline{\mu }\right\| ), \end{aligned}$$

and thus, by Young’s and Poincaré’s inequalities, and exploiting (4.11), we deduce

4.13 $$\begin{aligned} \left| \overline{ \mu }\right| =\frac{1}{\left| \Gamma _0\right| } \Vert F^{\prime }(\varphi )\Vert _{L^{1}(\Gamma (t) )}\le \frac{1}{\left| \Gamma _0\right| }C_1\left( C_3\left\| \nabla _\Gamma \mu \right\| +C\right) +\frac{1}{\left| \Gamma _0\right| }C_{2}, \end{aligned}$$

for some $C_3>0$, and thus

4.14 $$\begin{aligned} \left| \overline{ \mu }\right| ^2\le \frac{2}{\left| \Gamma _0\right| ^2}C_1^2C_3^2\left\| \nabla _\Gamma \mu \right\| ^2+\frac{2}{\left| \Gamma _0\right| ^2}\left( C_1C +C_{2}\right) ^2. \end{aligned}$$

Therefore, from (4.9) and all the inequalities above, by choosing $\epsilon =\frac{1}{8\left| \Gamma _0\right| ^2(C_1C_3)^2}$we end up with

$$\begin{aligned}&\frac{d}{dt}\left( \frac{1}{2}\Vert \nabla _\Gamma \varphi \Vert ^2+\int _{\Gamma (t)}(\Psi (\varphi )+\tilde{C})d\sigma \right) +\frac{1}{4}\int _{\Gamma (t)}\vert \nabla _\Gamma \mu \vert ^2d\sigma \\  &\quad \le C(1+\left\| {\textbf {u}}\right\| _{{\textbf {L}}^4(\Gamma (t))}^2)\left( \frac{1}{2}\Vert \nabla _\Gamma \varphi \Vert ^2+\int _{\Gamma (t)}(\Psi (\varphi )+\tilde{C})d\sigma \right) +C(1+\left\| {\textbf {u}}\right\| _{{\textbf {L}}^4(\Gamma (t))}^2),\\&\quad \forall t\in [0,\widetilde{T}]. \end{aligned}$$

Therefore, an application of Gronwall’s Lemma and (4.13), assuming that $\left\| {\textbf {u}}\right\| _{L^2_{{\textbf {H}}^1(\widetilde{T})}}\le C_A$, for some $C_A>0$, entail

4.15 $$\begin{aligned}&\left\| \varphi \right\| _{L^\infty _{H^1(\widetilde{T})}}+ \left\| \mu \right\| _{ L^2_{H^1(\widetilde{T})}}+\left\| \Psi (\varphi )\right\| _{L^\infty _{L^1(\widetilde{T})}}\le C(T,C_A) . \end{aligned}$$

Observe now that, by testing the equation for $\mu$ by $-\Delta _{\Gamma (t)}\varphi$ we immediately deduce, by standard estimates, since $F''\ge \theta$,

$$\begin{aligned} \left\| \Delta _{\Gamma (t)}\varphi \right\| ^2\le \left\| \Delta _{\Gamma (t)}\varphi \right\| ^2+\int _{\Gamma (t)}F''(\varphi )\left| \nabla _\Gamma \varphi \right| ^2d\sigma \le C(\left\| \nabla _\Gamma \mu \right\| \left\| \nabla _\Gamma \varphi \right\| +\left\| \nabla _\Gamma \varphi \right\| ^2), \end{aligned}$$

so that we also deduce

$$ \left\| \varphi \right\| _{L^4_{H^2(\widetilde{T})}}\le C(T,C_A), $$

from which we also deduce by comparison in the equation for $\mu$ that

4.16 $$\begin{aligned} \left\| F'(\varphi )\right\| _{L^2_{L^2(\widetilde{T})}}\le C(T,C_A). \end{aligned}$$

This last regularity result also allows to infer in the end, by standard arguments (after passing to the limit in the complete approximating scheme), that $\vert \varphi \vert <1$ almost everywhere on $\Gamma (t)$, for any $t\in [0,T]$.

In conclusion, by comparison it is now immediate to deduce, recalling $\left| \varphi \right| <1$, that

$$ \left\| \partial ^\circ _t\varphi \right\| _{H^1(\Gamma (t))'}\le C(\left\| \nabla _\Gamma \mu \right\| +\left\| {\textbf {v}}\right\| ), $$

and thus also

$$ \left\| \varphi \right\| _{H^1_{H^{-1}(\widetilde{T})}}\le C(T,C_A). $$

The regularity shown so far is enough to show the existence of a weak solution (notice that the initial data can be less regular, in particular it is enough that $\varphi _0\in H^1(\Gamma _0)$, $\left| \overline{\varphi }_0\right| <1$ and $\left\| \varphi _0\right\| _{L^\infty (\Gamma _0)}\le 1$). We now aim at showing higher-order estimates (always with the idea of being in the same Galerkin framework with the use of a potential $\Psi$ approximating the singular one as in [11]). Let us multiply equation (4.1)$_1$ by $\partial ^\circ _t\mu$. Again from (2.12) we infer

$$\begin{aligned} \frac{1}{2}\frac{d}{dt}\left\| \nabla _\Gamma \mu \right\| ^2&=\int _{\Gamma (t)}\nabla _\Gamma \partial ^\circ _t\mu \cdot \nabla _\Gamma \mu d\sigma +\int _{\Gamma (t)}\left| \nabla _\Gamma \mu \right| ^2Hv_{\textbf {n}}d\sigma \\&\quad -2\int _{\Gamma (t)}\nabla _\Gamma \mu \cdot {\mathcal {E}}_s({{\textbf {V}}}_{\textbf {n}})\nabla _\Gamma \mu d\sigma . \end{aligned}$$

Therefore we have

4.17 $$\begin{aligned}&\frac{1}{2} \frac{d}{dt}\left\| \nabla _\Gamma \mu \right\| ^2+\int _{\Gamma (t)}\partial ^\circ _t\varphi \partial ^\circ _t\mu d\sigma +\int _{\Gamma (t)}\nabla _\Gamma \varphi \cdot {\textbf {v}}\partial ^\circ _t\mu d\sigma \nonumber \\&\quad =\int _{\Gamma (t)}\left| \nabla _\Gamma \mu \right| ^2Hv_{\textbf {n}}d\sigma -2\int _{\Gamma (t)}\nabla _\Gamma \mu \cdot {\mathcal {E}}_s({{\textbf {V}}}_{\textbf {n}})\nabla _\Gamma ^T\mu d\sigma . \end{aligned}$$

Now, we can argue in the same way as to obtain [11, (2.36)], namely, assuming $\eta \in C^\infty _{H^1}$, we can write, exploiting (2.11) and (2.12),

4.18 $$\begin{aligned}&\frac{d}{dt}\int _{\Gamma (t)}\nabla _\Gamma \varphi \cdot \nabla _\Gamma \eta d\sigma +\frac{d}{dt}\int _{\Gamma (t)}\Psi '(\varphi )\eta d\sigma =\frac{d}{dt}\int _{\Gamma (t)}\mu \eta d\sigma \nonumber \\  &\quad =\int _{\Gamma (t)}\partial ^\circ _t\mu \eta d\sigma +\int _{\Gamma (t)}\mu \partial ^\circ _t\eta d\sigma +\int _{\Gamma (t)}\mu \eta \textrm{div}_\Gamma {{\textbf {V}}}_{\textbf {n}}d\sigma , \end{aligned}$$

but, recalling the definition of $\mu$,

$$\begin{aligned}&\frac{d}{dt}\int _{\Gamma (t)}\nabla _\Gamma \varphi \cdot \nabla _\Gamma \eta d\sigma +\frac{d}{dt}\int _{\Gamma (t)}\Psi '(\varphi )\eta d\sigma \\  &\quad =\int _{\Gamma (t)}\nabla _\Gamma \partial ^\circ _t\varphi \cdot \nabla _\Gamma \eta d\sigma +\int _{\Gamma (t)}\Psi ''(\varphi )\partial ^\circ _t\varphi \eta d\sigma +\int _{\Gamma (t)}\mu \partial ^\circ _t\eta d\sigma \\  &\qquad +\int _{\Gamma (t)}\nabla _\Gamma \varphi \cdot \nabla _\Gamma \eta \textrm{div}_\Gamma {{\textbf {V}}}_{\textbf {n}}d\sigma +\int _{\Gamma (t)}\Psi '(\varphi )\eta \textrm{div}_\Gamma {{\textbf {V}}}_{\textbf {n}}d\sigma \\&\qquad -2\int _{\Gamma (t)}\nabla _\Gamma \varphi \cdot {\mathcal {E}}_S({{\textbf {V}}}_{\textbf {n}})\nabla _\Gamma \eta d\sigma , \end{aligned}$$

so that, inserting in (4.18), we deduce

4.19 $$\begin{aligned} \int _{\Gamma (t)}\partial ^\circ _t\mu \eta d\sigma&=-\int _{\Gamma (t)}\mu \eta \textrm{div}_\Gamma {{\textbf {V}}}_{\textbf {n}}d\sigma +\int _{\Gamma (t)}\nabla _\Gamma \partial ^\circ _t\varphi \cdot \nabla _\Gamma \eta d\sigma +\int _{\Gamma (t)}\Psi ''(\varphi )\partial ^\circ _t\varphi \eta d\sigma \nonumber \\&\quad +\int _{\Gamma (t)}\nabla _\Gamma \varphi \cdot \nabla _\Gamma \eta \textrm{div}_\Gamma {{\textbf {V}}}_{\textbf {n}}d\sigma +\int _{\Gamma (t)}\Psi '(\varphi )\eta \textrm{div}_\Gamma {{\textbf {V}}}_{\textbf {n}}d\sigma \nonumber \\&\quad -2\int _{\Gamma (t)}\nabla _\Gamma \varphi \cdot {\mathcal {E}}_S({{\textbf {V}}}_{\textbf {n}})\nabla _\Gamma \eta d\sigma , \end{aligned}$$

and then by density it is immediate to deduce that this holds for any $\eta \in L^2_{H^1}$. By setting $\eta =\partial ^\circ _t\varphi$ we can then estimate the term $\int _{\Gamma (t)}\partial ^\circ _t\varphi \partial ^\circ _t\mu$. In the end we thus obtain from (4.17), recalling the definition of $\Psi$ and that $\textrm{div}_\Gamma {{\textbf {V}}}_{\textbf {n}}=H v_{\textbf {n}}$, that

4.20 $$\begin{aligned}&\frac{1}{2} \frac{d}{dt}\left\| \nabla _\Gamma \mu \right\| ^2+\left\| \nabla _\Gamma \partial ^\circ _t\varphi \right\| ^2+ (\partial ^\circ _t\varphi ,F''(\varphi )\partial ^\circ _t\varphi )+\int _{\Gamma (t)}\nabla _\Gamma \varphi \cdot {\textbf {v}}\partial ^\circ _t\mu d\sigma \nonumber \\&\quad =\theta _0\left\| \partial ^\circ _t\varphi \right\| ^2+\int _{\Gamma (t)}\left| \nabla _\Gamma \mu \right| ^2Hv_{\textbf {n}}d\sigma -2\int _{\Gamma (t)}\nabla _\Gamma \mu \cdot {\mathcal {E}}_s({{\textbf {V}}}_{\textbf {n}})\nabla _\Gamma \mu d\sigma \nonumber \\&\qquad +\int _{\Gamma (t)}\mu \partial ^\circ _t\varphi Hv_{\textbf {n}}d\sigma -\int _{\Gamma (t)}\nabla _\Gamma \varphi \cdot \nabla _\Gamma \partial ^\circ _t\varphi Hv_{\textbf {n}}d\sigma -\int _{\Gamma (t)}\Psi '(\varphi )\partial ^\circ _t\varphi Hv_{\textbf {n}}d\sigma \nonumber \\&\qquad +2\int _{\Gamma (t)}\nabla _\Gamma \varphi \cdot {\mathcal {E}}_S({{\textbf {V}}}_{\textbf {n}})\nabla _\Gamma \partial ^\circ _t\varphi d\sigma . \end{aligned}$$

Now we observe that, by the regularity of the flow map, and estimate (4.13), we have

$$\begin{aligned}&\left| \int _{\Gamma (t)}\left| \nabla _\Gamma \mu \right| ^2Hv_{\textbf {n}}d\sigma -2\int _{\Gamma (t)}\nabla _\Gamma \mu \cdot {\mathcal {E}}_s({{\textbf {V}}}_{\textbf {n}})\nabla _\Gamma \mu d\sigma +\int _{\Gamma (t)}\mu \partial ^\circ _t\varphi Hv_{\textbf {n}}d\sigma \right| \\&\quad \le C(1+\left\| \nabla _\Gamma \mu \right\| ^2)+C\left\| \partial ^\circ _t\varphi \right\| ^2, \end{aligned}$$

and, similarly, recalling that $\varphi \in L^\infty _{H^1}$,

$$\begin{aligned}&\left| -\int _{\Gamma (t)}\nabla _\Gamma \varphi \cdot \nabla _\Gamma \partial ^\circ _t\varphi Hv_{\textbf {n}}d\sigma +2\int _{\Gamma (t)}\nabla _\Gamma \varphi \cdot {\mathcal {E}}_S({{\textbf {V}}}_{\textbf {n}})\nabla _\Gamma \partial ^\circ _t\varphi d\sigma \right| \le C+\frac{1}{2}\left\| \nabla \partial ^\circ _t\varphi \right\| ^2. \end{aligned}$$

Now we need to control the term with $\Psi '$. We follow [11], showing some estimates also working in the Galerkin setting. In particular, observe that, by (2.11),

$$\begin{aligned} \frac{d}{dt}\int _{\Gamma (t)}\Psi (\varphi )Hv_{\textbf {n}}d\sigma&=\int _{\Gamma (t)}\Psi '(\varphi )\partial ^\circ _t\varphi Hv_{\textbf {n}}d\sigma +\int _{\Gamma (t)}\Psi (\varphi )\partial ^\circ _t(Hv_{\textbf {n}})d\sigma \\&\quad +\int _{\Gamma (t)}\Psi (\varphi )(Hv_{\textbf {n}})^2d\sigma , \end{aligned}$$

and also, by the regularity of the flow map, we have

$$\begin{aligned} \left| \int _{\Gamma (t)}\Psi (\varphi )\partial ^\circ _t(Hv_{\textbf {n}})d\sigma +\int _{\Gamma (t)}\Psi (\varphi )(Hv_{\textbf {n}})^2\right| d\sigma \le C\left\| \Psi (\varphi )\right\| _{L^1(\Gamma (t))}\le C, \end{aligned}$$

recalling (4.15). Therefore, recalling that $F''\ge \theta$, (4.20) becomes

4.21 $$\begin{aligned}&\frac{d}{dt}\left( \frac{1}{2}\left\| \nabla _\Gamma \mu \right\| ^2+\int _{\Gamma (t)}\Psi (\varphi )Hv_{\textbf {n}}\right) d\sigma +\frac{1}{2} \left\| \nabla _\Gamma \partial ^\circ _t\varphi \right\| ^2+ \theta \left\| \partial ^\circ _t\varphi \right\| ^2+\int _{\Gamma (t)}\nabla _\Gamma \varphi \cdot {\textbf {v}}\partial ^\circ _t\mu d\sigma \nonumber \\&\quad \le \tilde{C}\left\| \partial ^\circ _t\varphi \right\| ^2+C(1+\left\| \nabla _\Gamma \mu \right\| ^2), \end{aligned}$$

for some $\tilde{C}>0$. In order to control $\left\| \partial ^\circ _t\varphi \right\| ^2$, we test (4.1)$_1$ by $\partial ^\circ _t\varphi$ and integrate by parts. This gives, by standard estimates, recalling $\varphi \in L^\infty _{H^1}$,

4.22 $$\begin{aligned} \tilde{C}\left\| \partial ^\circ _t\varphi \right\| ^2&\le \left\| \nabla _\Gamma \mu \right\| \left\| \nabla _\Gamma \partial ^\circ _t\varphi \right\| +\left\| \varphi \right\| _{L^4(\Gamma (t))}\left\| {\textbf {v}}\right\| _{{\textbf {L}}^4(\Gamma (t))}\left\| \nabla _\Gamma \partial ^\circ _t\varphi \right\| \nonumber \\  &\le \frac{1}{8}\left\| \nabla _\Gamma \partial ^\circ _t\varphi \right\| ^2+C(\left\| \nabla _\Gamma \mu \right\| ^2+\left\| {\textbf {v}}\right\| _{{\textbf {L}}^4(\Gamma (t))}^2), \end{aligned}$$

Now, to estimate the advective term related to ${\textbf {v}}$, we have to work a bit more. First, if we consider the Galerkin setting, together with the approximation ${\textbf {u}}_k\in C^\infty _{{\textbf {H}}^1\cap {\textbf {L}}^2}$ of the velocity (here still denoted for simplicity by ${\textbf {v}}$), we have, by (2.11)-(2.13),

$$\begin{aligned}&{\int _{\Gamma (t)}\nabla _\Gamma \varphi \cdot {\textbf {v}}\partial ^\circ _t\mu d\sigma }=\frac{d}{dt}\int _{\Gamma (t)}\nabla _\Gamma \varphi \cdot {\textbf {v}}\mu d\sigma -\int _{\Gamma (t)}\nabla _\Gamma \partial ^\circ _t\varphi \cdot {\textbf {v}}\mu d\sigma -\int _{\Gamma (t)}\nabla _\Gamma \varphi \cdot \partial ^\circ _t{\textbf {v}}\mu d\sigma \\  &\quad -\int _{\Gamma (t)}\nabla _\Gamma \varphi \cdot {\textbf {v}}\mu Hv_{\textbf {n}}d\sigma +\int _{\Gamma (t)}{\textbf {v}}\mu \cdot (\nabla _\Gamma ^T{{\textbf {V}}}_{\textbf {n}})\nabla _\Gamma \varphi d\sigma , \end{aligned}$$

and by standard estimates, recalling (4.14) and the regularity of the flow map, we deduce

$$\begin{aligned}&\left| \int _{\Gamma (t)}\nabla _\Gamma \partial ^\circ _t\varphi \cdot {\textbf {v}}\mu d\sigma +\int _{\Gamma (t)}\nabla _\Gamma \varphi \cdot \partial ^\circ _t{\textbf {v}}\mu d\sigma +\int _{\Gamma (t)}\nabla _\Gamma \varphi \cdot {\textbf {v}}\mu Hv_{\textbf {n}}d\sigma -\int _{\Gamma (t)}{\textbf {v}}\mu \cdot (\nabla _\Gamma ^T{{\textbf {V}}}_{\textbf {n}})\nabla _\Gamma \varphi d\sigma \right| \\  &\quad \le C(1+\left\| \partial ^\circ _t{\textbf {v}}\right\| _{{\textbf {L}}^\infty (\Gamma (t))}+\left\| {\textbf {v}}\right\| _{{\textbf {L}}^\infty (\Gamma (t))}^2)(1+\left\| \nabla _\Gamma \mu \right\| ^2)+\frac{1}{8} \left\| \nabla _\Gamma \partial ^\circ _t\varphi \right\| ^2, \end{aligned}$$

and thus we obtain from (4.21) that

4.23 $$\begin{aligned}&\frac{d}{dt}\left( \frac{1}{2}\left\| \nabla _\Gamma \mu \right\| ^2+\int _{\Gamma (t)}\Psi (\varphi )Hv_{\textbf {n}}d\sigma +\int _{\Gamma (t)}\nabla _\Gamma \varphi \cdot {\textbf {v}}\mu d\sigma \right) +\frac{1}{4} \left\| \nabla _\Gamma \partial ^\circ _t\varphi \right\| ^2+ \theta \left\| \partial ^\circ _t\varphi \right\| ^2\nonumber \\&\quad \le C(1+\left\| \partial ^\circ _t{\textbf {v}}\right\| _{{\textbf {L}}^\infty (\Gamma (t))}+\left\| {\textbf {v}}\right\| _{{\textbf {L}}^\infty (\Gamma (t))}^2)(1+\left\| \nabla _\Gamma \mu \right\| ^2). \end{aligned}$$

Since $\textrm{div}_\Gamma {\textbf {v}}=0$, it holds

$$ \left| \int _{\Gamma (t)}\nabla _\Gamma \varphi \cdot {\textbf {v}}\mu d\sigma \right| =\left| -\int _{\Gamma (t)}\varphi {\textbf {v}}\cdot \nabla _\Gamma \mu d\sigma \right| \le \frac{1}{4}\left\| \nabla _\Gamma \mu \right\| ^2-C\left\| {\textbf {v}}\right\| _{{\textbf {L}}^\infty (\Gamma (t))}^2, $$

and also, by (4.15), we have

$$ \left| \int _{\Gamma (t)}\Psi (\varphi )Hv_{\textbf {n}}d\sigma \right| \le C. $$

Then, an application of Gronwall’s lemma, together with (4.13), ensures that $\mu \in L^\infty _{H^1(\widetilde{T})}$ and $\varphi \in H^1_{H^1(\widetilde{T})}$. As a matter of facts, this result depends on the approximating sequence ${\textbf {u}}_k$ and the approximation of the singular potential $\Psi$, but it is independent of the Galerkin parameters. Therefore, we can easily pass to the limit in these Galerkin parameters and think of finding uniform estimates in the parameter *k* of the approximating velocities and also uniform with respect to the approximation of $\Psi$.

We now start again from (4.20), assuming a solution after passing to the limit in the Galerkin scheme, which is sufficiently regular to perform the estimate, since the singular potential is still approximated by a regular one and the velocity is still the approximated one (i.e., ${\textbf {u}}_k$). We are allowed to perform estimates in the $L^p$ setting, which are not allowed in a Galerkin scheme. In particular, exactly with the same arguments as in [11, (3.60)], by testing the equation for $\mu$ by $F'(\varphi )\left| F'(\varphi )\right| ^{p-2}$, for $p\ge 2$ and applying suitable Hölder and Young’s inequalities, we can deduce, also recalling (4.13), that

4.24 $$\begin{aligned}&\left\| F'(\varphi )\right\| _{L^p(\Gamma (t))}^p+\int _{\Gamma (t)}(p-1)F''(\varphi )\left| F'(\varphi )\right| ^{p-2}\left| \nabla \varphi \right| ^2d\sigma \nonumber \\  &\quad \le \left\| \mu +\theta _0\varphi \right\| _{L^p(\Gamma (t))}\left\| F'(\varphi )\right\| ^{p-1}_{L^p(\Gamma (t))}\nonumber \\&\quad \le \frac{1}{2}\left\| F'(\varphi )\right\| _{L^p(\Gamma (t))}^p+C(p)\left\| \mu +\theta _0\varphi \right\| _{L^p(\Gamma (t))}^p, \end{aligned}$$

entailing, since $H^1(\Gamma (t))\hookrightarrow L^p(\Gamma (t))$ for any $p\ge 2$ ad recalling (4.13), that

4.25 $$\begin{aligned} \left\| F'(\varphi )\right\| _{L^p(\Gamma (t))}\le C(p)(1+\left\| \nabla _\Gamma \mu \right\| ), \end{aligned}$$

which also entails, since $\varphi \in L^\infty _{H^1(\widetilde{T})}$,

4.26 $$\begin{aligned} \left\| \Psi '(\varphi )\right\| _{L^p(\Gamma (t))}\le C(p)(1+\left\| \nabla _\Gamma \mu \right\| ). \end{aligned}$$

From the equation for $\mu$ we thus obtain, by elliptic regularity,

4.27 $$\begin{aligned} \left\| \varphi \right\| _{W^{2,p}(\Gamma (t))}\le C(p)(1+\left\| \nabla _\Gamma \mu \right\| ), \quad \forall \in [2,+\infty ). \end{aligned}$$

Now we can analyze the remaining advective term (4.21). Thanks to (4.19), with $\eta =\nabla _\Gamma \varphi \cdot {\textbf {v}}$, we infer

4.28 $$\begin{aligned}&\int _{\Gamma (t)}\partial ^\circ _t\mu \nabla _\Gamma \varphi \cdot {\textbf {v}}d\sigma =-\int _{\Gamma (t)}\mu \nabla _\Gamma \varphi \cdot {\textbf {v}}\textrm{div}_\Gamma {{\textbf {V}}}_{\textbf {n}}d\sigma +\int _{\Gamma (t)}\nabla _\Gamma \partial ^\circ _t\varphi \cdot \nabla _\Gamma (\nabla _\Gamma \varphi \cdot {\textbf {v}}))d\sigma \nonumber \\  &\quad +\int _{\Gamma (t)}\Psi ''(\varphi )\partial ^\circ _t\varphi \nabla _\Gamma \varphi \cdot {\textbf {v}}d\sigma \nonumber +\int _{\Gamma (t)}\nabla _\Gamma \varphi \cdot \nabla _\Gamma (\nabla _\Gamma \varphi \cdot {\textbf {v}}) \textrm{div}_\Gamma {{\textbf {V}}}_{\textbf {n}}d\sigma \nonumber \\&\quad +\int _{\Gamma (t)}\Psi '(\varphi )\nabla _\Gamma \varphi \cdot {\textbf {v}}\textrm{div}_\Gamma {{\textbf {V}}}_{\textbf {n}}d\sigma -2\int _{\Gamma (t)}\nabla _\Gamma \varphi \cdot {\mathcal {E}}_S({{\textbf {V}}}_{\textbf {n}})\nabla _\Gamma (\nabla _\Gamma \varphi \cdot {\textbf {v}})d\sigma . \end{aligned}$$

We can now estimate all the terms appearing in the right-hand side. First, we have, recalling (4.13) and (4.27) with $p=2$, and the regularity of the flow map,

$$\begin{aligned}&\left| \int _{\Gamma (t)}\mu \nabla _\Gamma \varphi \cdot {\textbf {v}}\textrm{div}_\Gamma {{\textbf {V}}}_{\textbf {n}}d\sigma \right| \le C\left\| \mu \right\| \left\| \nabla _\Gamma \varphi \right\| _{{\textbf {L}}^4(\Gamma (t))}\left\| {\textbf {v}}\right\| _{{\textbf {L}}^4(\Gamma (t))}\\  &\quad \le C(1+\left\| \nabla _\Gamma \mu \right\| )\left\| \varphi \right\| _{H^2(\Gamma (t))}(1+\left\| {\textbf {u}}\right\| _{{\textbf {L}}^4(\Gamma (t))})\le C(1+\left\| \nabla _\Gamma \mu \right\| ^2)(1+\left\| {\textbf {u}}\right\| _{{\textbf {L}}^4(\Gamma (t))}). \end{aligned}$$

Then, we have, recalling the regularity of the flow map and ${\textbf {v}}={\textbf {u}}+{{\textbf {V}}}_{\textbf {n}}$, the embedding $W^{2,4}(\Gamma (t))\hookrightarrow W^{1,\infty }(\Gamma (t))$ and (4.27) with $p=4$,

$$\begin{aligned}&\left| \int _{\Gamma (t)}\nabla _\Gamma \partial ^\circ _t\varphi \cdot \nabla _\Gamma (\nabla _\Gamma \varphi \cdot {\textbf {v}}))d\sigma \right| +\left| \int _{\Gamma (t)}\nabla _\Gamma \varphi \cdot \nabla _\Gamma (\nabla _\Gamma \varphi \cdot {\textbf {v}}) \textrm{div}_\Gamma {{\textbf {V}}}_{\textbf {n}}d\sigma \right| \\  &\qquad +\left| 2\int _{\Gamma (t)}\nabla _\Gamma \varphi \cdot {\mathcal {E}}_S({{\textbf {V}}}_{\textbf {n}})\nabla _\Gamma (\nabla _\Gamma \varphi \cdot {\textbf {v}})d\sigma \right| \\  &\quad \le C(\left\| \nabla _\Gamma \partial ^\circ _t\varphi \right\| +\left\| \nabla _\Gamma \varphi \right\| )\left( \left\| \nabla _\Gamma \varphi \right\| _{{\textbf {L}}^\infty (\Gamma (t))}(1+\left\| {\textbf {u}}\right\| _{{\textbf {H}}^1(\Gamma (t))})+\left\| \varphi \right\| _{W^{2,4}(\Gamma (t))}(1+\left\| {\textbf {u}}\right\| _{{\textbf {L}}^4(\Gamma (t))})\right) \\  &\quad \le C(1+\left\| \nabla _\Gamma \partial ^\circ _t\varphi \right\| )\left( \left\| \varphi \right\| _{W^{2,4}(\Gamma (t))}(1+\left\| {\textbf {u}}\right\| _{{\textbf {H}}^1(\Gamma (t))})+\left\| \varphi \right\| _{W^{2,4}(\Gamma (t))}(1+\left\| {\textbf {u}}\right\| _{{\textbf {L}}^4(\Gamma (t))})\right) \\  &\quad \le C(1+\left\| \nabla _\Gamma \partial ^\circ _t\varphi \right\| )(1+\left\| \nabla _\Gamma \mu \right\| )(1+\left\| {\textbf {u}}\right\| _{{\textbf {H}}^1(\Gamma (t))}+\left\| {\textbf {u}}\right\| _{{\textbf {L}}^4(\Gamma (t)})\\  &\quad \le \frac{1}{16}\left\| \nabla _\Gamma \partial ^\circ _t\varphi \right\| ^2+\left\| \nabla \mu \right\| ^2(1+\left\| {\textbf {u}}\right\| _{{\textbf {H}}^1(\Gamma (t))}^2+\left\| {\textbf {u}}\right\| _{{\textbf {L}}^4(\Gamma (t))}^2)+C(1+\left\| {\textbf {u}}\right\| _{{\textbf {H}}^1(\Gamma (t))}^2+\left\| {\textbf {u}}\right\| _{{\textbf {L}}^4(\Gamma (t))}^2). \end{aligned}$$

Then we have, integrating by parts recalling $\textrm{div}_\Gamma {\textbf {v}}=0$ and (4.26), and by Hölder’s and Young’s inequalities,

$$\begin{aligned} \left| \int _{\Gamma (t)}\Psi ''(\varphi )\partial ^\circ _t\varphi \nabla _\Gamma \varphi \cdot {\textbf {v}}d\sigma \right|&= \left| \int _{\Gamma (t)}\nabla _\Gamma \Psi '(\varphi )\partial ^\circ _t\varphi \cdot {\textbf {v}}d\sigma \right| \\  &\le \left| \int _{\Gamma (t)}\Psi '(\varphi )\nabla _\Gamma \partial ^\circ _t\varphi \cdot {\textbf {v}}d\sigma \right| +\left| \int _{\Gamma (t)}\Psi '(\varphi )\partial ^\circ _t\varphi Hv_{\textbf {n}}d\sigma \right| \\  &\le \left\| \nabla _\Gamma \partial ^\circ _t\varphi \right\| \left\| \Psi '(\varphi )\right\| _{L^4(\Gamma (t))}\left\| {\textbf {v}}\right\| _{{\textbf {L}}^4(\Gamma (t))}+C\left\| \Psi '(\varphi )\right\| \left\| \partial ^\circ _t\varphi \right\| \\  &\le \frac{1}{16}\left\| \nabla _\Gamma \partial ^\circ _t\varphi \right\| ^2+\frac{\theta }{2}\left\| \partial ^\circ _t\varphi \right\| ^2+C(1+\left\| \nabla _\Gamma \mu \right\| ^2)(1+\left\| {\textbf {u}}\right\| _{{\textbf {L}}^4(\Gamma (t))}^2). \end{aligned}$$

Proceeding in the estimates, we are left with

$$\begin{aligned} \left| \int _{\Gamma (t)}\Psi '(\varphi )\nabla _\Gamma \varphi \cdot {\textbf {v}}\textrm{div}_\Gamma {{\textbf {V}}}_{\textbf {n}}d\sigma \right|&\le C\left\| \Psi '(\varphi )\right\| _{L^4(\Gamma (t))}\left\| \nabla _\Gamma \varphi \right\| \left\| {\textbf {v}}\right\| _{{\textbf {L}}^4(\Gamma (t))}\\  &\le C\left\| \Psi '(\varphi )\right\| _{L^4(\Gamma (t))}\left\| {\textbf {v}}\right\| _{{\textbf {L}}^4(\Gamma (t))}\\  &\le C\left\| \nabla _\Gamma \mu \right\| \left\| {\textbf {v}}\right\| _{{\textbf {L}}^4(\Gamma (t))}+C(1+\left\| {\textbf {u}}\right\| _{{\textbf {L}}^4(\Gamma (t))}), \end{aligned}$$

where again we exploited (4.26). To sum up all these estimates, coming back to (4.21) and using also (4.22), we end up with

4.29 $$\begin{aligned}&\frac{d}{dt}\frac{1}{2}\left\| \nabla _\Gamma \mu \right\| ^2+\frac{1}{4} \left\| \nabla _\Gamma \partial ^\circ _t\varphi \right\| ^2+ \frac{\theta }{2}\left\| \partial ^\circ _t\varphi \right\| ^2 \\&\quad \le C\left\| \nabla _\Gamma \mu \right\| ^2(1+\left\| {\textbf {u}}\right\| ^2_{{\textbf {H}}^1(\Gamma (t))}+\left\| {\textbf {u}}\right\| _{{\textbf {L}}^4(\Gamma (t))}^2)+C(1+\left\| {\textbf {u}}\right\| ^2_{{\textbf {H}}^1(\Gamma (t))}+\left\| {\textbf {u}}\right\| _{{\textbf {L}}^4(\Gamma (t))}^2)\nonumber \end{aligned}$$

from which, since ${\textbf {u}}\in L^2_{{\textbf {H}}^1(\widetilde{T})}$, $\left\| {\textbf {u}}\right\| _{L^2_{{\textbf {H}}^1(\widetilde{T})}}\le C_A$, and ${\textbf {H}}^1(\Gamma (t))\hookrightarrow {\textbf {L}}^4(\Gamma (t))$, by Gronwall’s Lemma we infer, since $\mu _0\in H^1(\Gamma _0)$ and recalling once more (4.14), that

4.30 $$\begin{aligned} \left\| \mu \right\| _{L^\infty _{H^1(\widetilde{T})}}+\left\| \partial ^\circ _t\varphi \right\| _{L^2_{H^1(\widetilde{T})}}\le C(T,C_A), \end{aligned}$$

this time independently on *any* approximating parameter. Also, thanks to (4.25) and (4.27), we get, for any $p\in [2,\infty )$,

4.31 $$\begin{aligned} \left\| \varphi \right\| _{L^\infty _{W^{2,p}(\widetilde{T})}}+\left\| F'(\varphi )\right\| _{L^\infty _{L^p(\widetilde{T})}}\le C(p,T,C_A). \end{aligned}$$

Now, by elliptic regularity (see also (2.10)) applied to (4.1)$_1$ we deduce, recalling $\varphi \in L^\infty _{W^{2,p}(\widetilde{T})}$ for any $p\ge 2$, and $\mu \in L^\infty _{H^1(\widetilde{T})}$,

$$\begin{aligned} \left\| \mu \right\| _{H^3(\Gamma (t))}&\le C(T)({1}+\left\| \partial ^\circ _t\varphi \right\| _{H^1(\Gamma (t))}+\left\| {\textbf {v}}\cdot \nabla _\Gamma \varphi \right\| _{H^1(\Gamma (t))})\\&\le C(T)({1}+\left\| \partial ^\circ _t\varphi \right\| _{H^1(\Gamma (t))}+\left\| {\textbf {u}}\right\| _{{\textbf {H}}^1(\Gamma (t))}), \end{aligned}$$

so that $\mu \in L^2_{H^3(\widetilde{T})}$. All these estimates allow to conclude the regularity part of the theorem, since now we can pass to the limit in the approximating sequence $\{{\textbf {u}}_k\}_k$ and in the approximating sequence of regular potentials to conclude. We are left to discuss the strict separation property, which can be retrieved *a posteriori*. In particular, by repeating the computations leading to (4.24) (this time for the solution without any approximating scheme) and, recalling that, since we are using the singular potential $\Psi$, it holds $\left| \varphi \right| <1$ for almost any *t* and $x\in \Gamma (t)$, one more precisely obtains, recalling (2.8) and (4.30),

4.32 $$\begin{aligned}&\left\| \mu \right\| _{L^p(\Gamma (t))}+\left\| F'(\varphi )\right\| _{L^p(\Gamma (t))}\le C(1+\left\| \mu \right\| _{L^p(\Gamma (t))})\nonumber \\&\le C(T)\sqrt{p}(1+\left\| \mu \right\| _{H^1(\Gamma (t))})\nonumber \\  &\le C(T,C_A)\sqrt{p}\quad \forall p\in [2,+\infty ),\quad \text { for a.a. }t\in [0,\widetilde{T}]. \end{aligned}$$

Thanks to this inequality and assumption (2.17) on $F'$, and recalling (2.8), we can reproduce word by word the De Giorgi iteration scheme applied on the equation for the chemical potential

$$\mu (t)=-\Delta _\Gamma \varphi (t)+\Psi (\varphi (t)),$$

for almost any $t\in [0,\widetilde{T}]$ as in the proof of [16, Theorem 3.3]. Note that the scheme can be performed *at any* fixed time $t\ge 0$ for which the uniform estimate (4.32) is valid, since it is based only on the definition of $\mu (t)$ and on the Gagliardo-Nirenberg’s inequality (2.8) with uniform constants on [0, *T*] (see inequality [16, (3.20)] for its application). Therefore, since we have assumed $\mu _0\in H^1(\Gamma _0)$, (2.8) and (4.32) also hold for $t=0$, and thus in the proof of [16, Theorem 3.3] we can also obtain the result for $t=0$, and deduce that there exists $\delta =\delta (T,C_A)>0$ such that

4.33 $$\begin{aligned} \sup _{t\in [0,\widetilde{T}]}\left\| \varphi \right\| _{C(\Gamma (t))}\le 1-\delta . \end{aligned}$$

The last regularity $\varphi \in L^\infty _{H^3(\widetilde{T})}\cap L^2_{H^4(\widetilde{T})}$ is obtained by elliptic regularity from

$$ -\Delta _\Gamma \varphi =-\Psi '(\varphi )+\mu , $$

and, thanks to the regularity of $\Psi$,

$$ \left\| -\Psi '(\varphi )+\mu \right\| _{L^\infty _{H^1(\widetilde{T})}}+\left\| -\Psi '(\varphi )+\mu \right\| _{L^2_{W^{2,p}(\widetilde{T})}}\le C(T,C_A), $$

which holds thanks to the separation property. In conclusion, again setting this estimate in a suitable approximation scheme (or, for instance, by means of increment quotients), by standard estimates we deduce from (4.19) that

$$\begin{aligned} \left| <\partial ^\circ _t\mu ,\eta >_{H^{-1},H^{1}}\right|&\le C(\left\| \mu \right\| +\left\| \nabla _\Gamma \partial ^\circ _t\varphi \right\| +\left\| \Psi ''(\varphi )\right\| _{L^\infty (\Gamma (t))}\left\| \partial ^\circ _t\varphi \right\| \\&\quad +\left\| \nabla _\Gamma \varphi \right\| +\left\| \Psi '(\varphi )\right\| _{L^\infty (\Gamma (t))})\left\| \eta \right\| _{H^1(\Gamma (t))}, \end{aligned}$$

for all $\eta \in H^1(\Gamma (t))$. Therefore, we immediately infer from the regularities above that

$$ \left\| \partial ^\circ _t\mu \right\| _{L^2_{H^{-1}(\widetilde{T})}}\le C(T,C_A), $$

concluding the proof of the theorem. $\square$

## Proof of Theorem 3.3

We divide the proof in many steps. First, we show the local existence of a strong solution according to Definition 3.1. We exploit the result of [4, Theorem 4.1].

**Step 0. Existence of a local strong solution on an interval** $[0,T_0]$. Observe that, thanks to Remark 4.3, the initial data are such that

$$ {\textbf {v}}_0\in {\textbf {H}}^1(\Gamma _0),\quad \varphi _0\in H^3(\Gamma _0),$$

and

$$\exists \delta _0\in (0,1):\ \left\| \varphi \right\| _{L^\infty (\Gamma _0)}\le 1-2\delta _0. $$

Then there exist $p>4$ and $q\in (2,4)$ such that

5.1 $$\begin{aligned} H^3(\Gamma _0)\hookrightarrow W^{4+\epsilon -\frac{4}{q},p}(\Gamma _0)\hookrightarrow B^{4-\frac{4}{q}}_{p,q}(\Gamma _0), \end{aligned}$$

for $\epsilon >0$ sufficiently small (for instance, we can choose $p=\tfrac{9}{2}$, $q=\tfrac{5}{2}$ and $\epsilon =\tfrac{2}{45}$). Thus the initial data and the functions $\Psi ,\nu ,\rho$ are sufficiently regular to apply [4, Theorem 4.1], recalling [4, Remarks 4.3$-$4.4]. This means that there exists $0<\widetilde{T}\le T$ and $(\tilde{{\textbf {v}}},\pi ,\varphi ,\mu )$, solution to (1.1), such that

$$\begin{aligned} \tilde{{\textbf {v}}}\in L^\infty _{{\textbf {L}}^2}\cap L^2_{{\textbf {H}}^2(\widetilde{T})}\cap H^1_{{\textbf {L}}^2(\widetilde{T})},\quad \pi \in L^2_{H^1(\widetilde{T})},\quad \varphi \in L^2_{W^{4,p}(\widetilde{T})}\cap H^1_{L^p(\widetilde{T})},\quad \mu \in L^2_{W^{2,p}(\widetilde{T})}, \end{aligned}$$

and

5.2 $$\begin{aligned} \sup _{t\in [0,\widetilde{T}]}\left\| \varphi (t)\right\| _{L^\infty (\Gamma (t))}\le 1-\delta _0. \end{aligned}$$

Now consider a strong solution $\varphi _2$ to (4.1) with ${\textbf {v}}:= \tilde{{\textbf {v}}}+{{\textbf {V}}}_{\textbf {n}}$ and initial datum $\varphi _0$. Since the velocity $\tilde{{\textbf {v}}}$ is sufficiently regular and since, by construction, $\textrm{div}_\Gamma (\tilde{{\textbf {v}}}+{{\textbf {V}}}_{\textbf {n}})=0$, this solution exists by Theorem 4.1. Now we can set $\varphi _1=\varphi$ and repeat word by word the proof leading to the continuous dependence estimate (4.7). This entails $\varphi \equiv \varphi _2$, so that we also deduce the regularity

$$ \varphi \in L^\infty _{W^{2,p}(\widetilde{T})}\cap H^1_{H^1(\widetilde{T})},\quad \mu \in L^\infty _{H^1(\widetilde{T})}\cap L^2_{H^3(\widetilde{T})},\quad \forall p\in [2,+\infty ). $$

Therefore we have shown that $(\tilde{{\textbf {v}}},\pi ,\varphi )$ is a local strong solution to (1.1) on $[0,T_0]$ (with $T_0=\widetilde{T}$), according to Definition 3.1, concluding the proof of local in time existence of a strong solution.

*Step 1. A problem for a divergence-free velocity.* We now need to introduce a problem for a divergence-free velocity, as done in [4, Section 5]. Namely, we solve the problem

$$\begin{aligned} -\Delta _\Gamma \Pi =Hv_{\textbf {n}}\end{aligned}$$

for $\Pi$ such that $\overline{\Pi }=0$. This admits a unique solution by elliptic theory. We then define $\widehat{{\textbf {u}}}=\nabla _\Gamma \Pi$ and set ${\textbf {v}}={{\textbf {V}}}+\widehat{{\textbf {u}}}$. Then, thanks to [4, Lemma A.1], the regularity on the flow map ensures that we have $\Pi \in C^1_{W^{4,p}}$, and then $\widehat{{\textbf {u}}}\in C^0_{{\textbf {W}}^{3,p}}$ with ${\textbf {P}}\partial ^\circ _t\widehat{{\textbf {u}}}\in C^0_{{\textbf {W}}^{3,p}}$, for any $p\in [2,+\infty )$, meaning also that $\widehat{{\textbf {u}}}\in C^0_{\mathcal {C}^2}$ as well as ${\textbf {P}}\partial ^\circ _t\widehat{{\textbf {u}}}\in C^0_{C^2}$. In this way, we obtain the following equivalent problem: find $({{\textbf {V}}},\pi ,\varphi )$ such that

5.3 $$\begin{aligned} {\left\{ \begin{array}{ll} \rho {\textbf {P}}\partial ^\circ _t{{\textbf {V}}}+((\rho {{\textbf {V}}}+{\textbf {J}}_\rho )\cdot \nabla _\Gamma ){{\textbf {V}}}+\rho (\widehat{{\textbf {u}}}\cdot \nabla _\Gamma ){{\textbf {V}}}+\rho ({{\textbf {V}}}\cdot \nabla _\Gamma )\widehat{{\textbf {u}}}+\rho v_{\textbf {n}}{\textbf {H}}{{\textbf {V}}}-2{\textbf {P}}\textrm{div}_\Gamma (\nu (\varphi ){\mathcal {E}}_S({{\textbf {V}}}))\\ \quad +v_{\textbf {n}}{\textbf {H}}{\textbf {J}}_\rho +\nabla _\Gamma \pi =-{\textbf {P}}\textrm{div}_\Gamma (\nabla _\Gamma \varphi \otimes \nabla _\Gamma \varphi )+2{\textbf {P}}\textrm{div}_\Gamma (\nu (\varphi )v_{\textbf {n}}{\textbf {H}})+\frac{\rho }{2}\nabla _\Gamma (v_{\textbf {n}})^2\\ \quad -\rho {\textbf {P}}\partial ^\circ _t\widehat{{\textbf {u}}} -((\rho \widehat{{\textbf {u}}}+{\textbf {J}}_\rho )\cdot \nabla _\Gamma )\widehat{{\textbf {u}}}-\rho v_{\textbf {n}}{\textbf {H}}\widehat{{\textbf {u}}}+2{\textbf {P}}\textrm{div}_\Gamma (\nu (\varphi ){\mathcal {E}}_S(\widehat{{\textbf {u}}})),\\ \textrm{div}_\Gamma {{\textbf {V}}}=0, \\ \partial ^\circ _t\varphi -\Delta _{\Gamma }\mu +\nabla _\Gamma \varphi \cdot {{\textbf {V}}}+\nabla _\Gamma \varphi \cdot \widehat{{\textbf {u}}}=0,\\ \mu =-\Delta _\Gamma \varphi +\Psi '(\varphi ). \end{array}\right. } \end{aligned}$$

Here we have ${{\textbf {V}}}(0)={{\textbf {V}}}_0:={\textbf {v}}_0-\widehat{{\textbf {u}}}(0)$ and $\varphi (0)=\varphi _0$. We will exploit this equivalent problem in the next step.

*Step 2. Extension of *$$T_0$$ **to** *T*. We need to show that actually there exists a strong solution which is globally defined over [0, *T*], by means of a contradiction argument using energy estimates. Assume then by contradiction that there exists a maximal time of existence $$T_m\in [T_0,T)$$. This means that there is no strict extension of $$({\textbf {v}}, p,\varphi )$$ defined on a strictly larger time interval $$[0,T_m')$$ being also a local strong solution therein.

Then it holds, for any $0<\widehat{T}<T_m$,

$$\begin{aligned}&{{\textbf {v}}} \in L ^2_{{\textbf {H}}^2(\widehat{T})}\cap H^1_{{\textbf {L}}^2_\sigma (\widehat{T})} ,\quad p\in L^2_{H^1(\widehat{T})},\quad \varphi \in L^\infty _{W^{2,q}(\widehat{T})} \cap H^1_{{H}^1(\widehat{T})} ,\quad \forall q\in [2,+\infty ),\\  &\left| \varphi \right| <1\quad \text {a.e. in }\Gamma (t) \quad \text {for a.a. }t\in [0,T_m), \quad \mu \in {L}^\infty _{H^1(\widehat{T})}\cap L^2_{H^3(\widehat{T})}. \end{aligned}$$

Now we consider the corresponding triple $({{\textbf {V}}},p,\varphi )$, satisfying (5.3), i.e., we set ${{\textbf {V}}}={\textbf {v}}-\widehat{{\textbf {u}}}$. This divergence-free velocity enjoys the same regularity properties as ${\textbf {v}}$. Now we show that all the norms in the spaces above are uniformly bounded by a constant in $(0,T_m)$ and this allows to define the solution also at $\widehat{T}=T_m$. First, let us observe that the regularity above is enough to perform all the following computations, which are therefore rigorous in this framework. We divide the estimates in many steps. All the estimates are related to problem (5.3).

*First estimate: energy identity. *We primarily notice that it holds, recalling the definition of $\mu$,

5.4 $$\begin{aligned} -&{\textbf {P}}\textrm{div}_\Gamma (\nabla _\Gamma \varphi \otimes \nabla \varphi )=-\nabla _\Gamma \varphi \Delta _{\Gamma }\varphi -\nabla _\Gamma (\nabla _\Gamma \varphi )\nabla _\Gamma \varphi \nonumber \\&=\nabla _\Gamma \varphi (\mu -\Psi '(\varphi ))-\frac{1}{2}\nabla _\Gamma (\vert \nabla _\Gamma \varphi \vert ^2) =\mu \nabla _\Gamma \varphi -\nabla _\Gamma \left( \Psi (\varphi )-\frac{1}{2}\vert \nabla _\Gamma \varphi \vert ^2\right) . \end{aligned}$$

Note that, in the first identity, we exploited

$$\begin{aligned} \textrm{div}_\Gamma (\nabla _\Gamma \varphi \otimes \nabla \varphi )=-\nabla _\Gamma \varphi \Delta \varphi -\widehat{\nabla }_\Gamma (\nabla _\Gamma \varphi )\nabla _\Gamma \varphi , \end{aligned}$$

and then observed that (see, for instance, ([8], Lemma 15))

$$\begin{aligned} \widehat{\nabla }_\Gamma (\nabla _\Gamma \varphi )\nabla _\Gamma \varphi =\frac{1}{2} \nabla _\Gamma \left| \nabla _\Gamma \varphi \right| ^2-[\nabla _\Gamma \varphi \cdot ((\nabla _\Gamma {{\textbf {n}}})\nabla _\Gamma \varphi )]{{\textbf {n}}}+({{\textbf {n}}}\cdot \nabla _\Gamma \varphi )((\nabla _\Gamma {{\textbf {n}}})\nabla _\Gamma \varphi ), \end{aligned}$$

so that

$$ {\nabla }_\Gamma (\nabla _\Gamma \varphi )\nabla _\Gamma \varphi ={\textbf {P}}\widehat{\nabla }_\Gamma (\nabla _\Gamma \varphi )\nabla _\Gamma \varphi =\frac{1}{2} \nabla _\Gamma \left| \nabla _\Gamma \varphi \right| ^2. $$

Therefore, up to redefining the pressure $\tilde{p}=p+\Psi (\varphi )+\frac{1}{2}\vert \nabla _\Gamma \varphi \vert ^2$ we can substitute the quantity $- {\textbf {P}}\textrm{div}_\Gamma (\nabla _\Gamma \varphi \otimes \nabla \varphi )$ with $\mu \nabla _\Gamma \varphi$. Again, in the estimates we will consider system (5.3), and then recover the original velocity. Note that, by (2.11), for any $t\in [0,T_m]$,

5.5 $$\begin{aligned} \frac{1}{2}\frac{d}{dt}\int _{\Gamma (t)}\rho \left| {{\textbf {V}}}\right| ^2d\sigma =\frac{1}{2} \int _{\Gamma (t)}\partial ^\circ _t\rho \left| {{\textbf {V}}}\right| ^2d\sigma +\int _{\Gamma (t)}\rho {{\textbf {V}}}\cdot \partial ^\circ _t{{\textbf {V}}}d\sigma +\frac{1}{2} \int _{\Gamma (t)}\rho \left| {{\textbf {V}}}\right| ^2Hv_{\textbf {n}}d\sigma . \end{aligned}$$

Moreover, after integration by parts, recalling that ${\textbf {J}}_\rho \cdot {\textbf {n}}=0$,

5.6 $$\begin{aligned} 0=\int _{\Gamma (t)}\textrm{div}_\Gamma \left( \frac{1}{2}\left| {{\textbf {V}}}\right| ^2{\textbf {J}}_\rho \right) d\sigma =\int _{\Gamma (t)}({\textbf {J}}_\rho \cdot \nabla _\Gamma ){{\textbf {V}}}\cdot {{\textbf {V}}}d\sigma +\int _{\Gamma (t)}\frac{1}{2} \left| {{\textbf {V}}}\right| ^2\textrm{div}_\Gamma ({\textbf {J}}_\rho )d\sigma , \end{aligned}$$

as well as, since ${{\textbf {V}}}\cdot {\textbf {n}}=0$ and $\textrm{div}_\Gamma {{\textbf {V}}}=-\textrm{div}_\Gamma \widehat{{\textbf {u}}}=-Hv_{\textbf {n}}$ by construction,

5.7 $$\begin{aligned}&0=\int _{\Gamma (t)}\textrm{div}_\Gamma \left( \rho {{\textbf {V}}}\frac{1}{2}\left| {{\textbf {V}}}\right| ^2\right) d\sigma =\int _{\Gamma (t)}{{\textbf {V}}}\cdot \nabla _\Gamma \rho \frac{1}{2}\left| {{\textbf {V}}}\right| ^2d\sigma +\int _{\Gamma (t)}\rho \frac{1}{2}\left| {{\textbf {V}}}\right| ^2\textrm{div}_\Gamma {{\textbf {V}}}d\sigma \nonumber \\&\qquad +\int _{\Gamma (t)}\rho ({{\textbf {V}}}\cdot \nabla _\Gamma ){{\textbf {V}}}\cdot {{\textbf {V}}}d\sigma \nonumber \\&\,\,\,=\int _{\Gamma (t)}{{\textbf {V}}}\cdot \nabla _\Gamma \rho \frac{1}{2}\left| {{\textbf {V}}}\right| ^2d\sigma -\int _{\Gamma (t)}\frac{1}{2}\rho \left| {{\textbf {V}}}\right| ^2Hv_{\textbf {n}}d\sigma +\int _{\Gamma (t)}\rho ({{\textbf {V}}}\cdot \nabla _\Gamma ){{\textbf {V}}}\cdot {{\textbf {V}}}d\sigma . \end{aligned}$$

Additionally, we get, recalling that the density $\rho$ satisfies (see (1.2)), since $\textrm{div}_\Gamma ({{\textbf {V}}}+\widehat{{\textbf {u}}}+{{\textbf {V}}}_{\textbf {n}})=0$,

$$ 0=\partial ^\circ _t\rho +\textrm{div}_\Gamma ({\textbf {J}}_\rho )+\textrm{div}_\Gamma ( \rho ({{\textbf {V}}}+\widehat{{\textbf {u}}}+{{\textbf {V}}}_{\textbf {n}}))=\partial ^\circ _t\rho +\textrm{div}_\Gamma ({\textbf {J}}_\rho )+ \nabla _\Gamma \rho \cdot ({{\textbf {V}}}+\widehat{{\textbf {u}}}), $$

we deduce, after an integration by parts on the term with $\widehat{ {\textbf {u}}}$, noting that $\textrm{div}_\Gamma \widehat{{\textbf {u}}}=Hv_{\textbf {n}}$,

$$\begin{aligned}&\frac{1}{2} \int _{\Gamma (t)}\partial ^\circ _t\rho \left| {{\textbf {V}}}\right| ^2d\sigma =-\int _{\Gamma (t)}\textrm{div}_\Gamma ({\textbf {J}}_\rho )\frac{1}{2}\left| {{\textbf {V}}}\right| ^2d\sigma -\int _{\Gamma (t)}\frac{1}{2}\left| {{\textbf {V}}}\right| ^2\nabla _\Gamma \rho \cdot {{\textbf {V}}}d\sigma \\  &\quad +\int _{\Gamma (t)}\frac{1}{2}\rho \left| {{\textbf {V}}}\right| ^2\rho Hv_{\textbf {n}}d\sigma +\int _{\Gamma (t)}\rho (\nabla _\Gamma {{\textbf {V}}})\widehat{{\textbf {u}}}\cdot {{\textbf {V}}}d\sigma . \end{aligned}$$

Then, from (5.6) and (5.7), we deduce

$$\begin{aligned} \frac{1}{2} \int _{\Gamma (t)}\partial ^\circ _t\rho \left| {{\textbf {V}}}\right| ^2d\sigma = \int _{\Gamma (t)}(\nabla _\Gamma {{\textbf {V}}})({\textbf {J}}_\rho +\rho ({{\textbf {V}}}+\widehat{{\textbf {u}}}))\cdot {{\textbf {V}}}d\sigma . \end{aligned}$$

Exploiting (5.3)$_1$ and (5.4), we also deduce, integrating by parts,

$$\begin{aligned} \int _{\Gamma (t)}\rho {{\textbf {V}}}\cdot \partial ^\circ _t{{\textbf {V}}}d\sigma =&-\int _{\Gamma (t)}(({\textbf {J}}_\rho +\rho ({{\textbf {V}}}+\widehat{{\textbf {u}}}))\cdot \nabla _\Gamma ){{\textbf {V}}})\cdot {{\textbf {V}}}d\sigma -\int _{\Gamma (t)}\rho ({{\textbf {V}}}\cdot \nabla _\Gamma )\widehat{{\textbf {u}}}\cdot {{\textbf {V}}}d\sigma \\  &-\int _{\Gamma (t)}\rho v_{\textbf {n}}{\textbf {H}}{{{\textbf {V}}}}\cdot {{\textbf {V}}}d\sigma -\int _{\Gamma (t)}v_{\textbf {n}}{\textbf {H}}{\textbf {J}}_\rho \cdot {{\textbf {V}}}-2\int _{\Gamma (t)}\nu (\varphi )\left| {\mathcal {E}}_S({{\textbf {V}}})\right| ^2d\sigma \\  &+\int _{\Gamma (t)}\mu \nabla _\Gamma \varphi \cdot {{\textbf {V}}}d\sigma -2\int _{\Gamma (t)}\nu (\varphi )v_{\textbf {n}}{\textbf {H}}:\nabla _\Gamma {{\textbf {V}}}d\sigma +\frac{1}{2}\int _{\Gamma (t)}\rho \nabla _\Gamma (v_{\textbf {n}})^2\cdot {{\textbf {V}}}d\sigma \\  &-\int _{\Gamma (t)}\rho \partial ^\circ _t\widehat{{\textbf {u}}}\cdot {{\textbf {V}}}-((\rho \widehat{{\textbf {u}}}+{\textbf {J}}_\rho )\cdot \nabla _\Gamma )\widehat{{\textbf {u}}}\cdot {{\textbf {V}}}d\sigma \\  &-\int _{\Gamma (t)}\rho v_{\textbf {n}}{\textbf {H}}\widehat{{\textbf {u}}}\cdot {{\textbf {V}}}d\sigma -2\int _{\Gamma (t)}\nu (\varphi ){\mathcal {E}}_S(\widehat{{\textbf {u}}}):{\mathcal {E}}_S({{\textbf {V}}})d\sigma . \end{aligned}$$

Putting all these identities above in (5.5), we infer

$$\begin{aligned}&\frac{1}{2}\frac{d}{dt}\int _{\Gamma (t)}\rho \left| {{\textbf {V}}}\right| ^2d\sigma +2\int _{\Gamma (t)}\nu (\varphi )\left| {\mathcal {E}}_S({{\textbf {V}}})\right| ^2d\sigma =\frac{1}{2}\int _{\Gamma (t)}\rho \left| {{\textbf {V}}}\right| ^2H v_{\textbf {n}}d\sigma \\  &\quad -\int _{\Gamma (t)}\rho ({{\textbf {V}}}\cdot \nabla _\Gamma )\widehat{{\textbf {u}}}\cdot {{\textbf {V}}}d\sigma -\int _{\Gamma (t)}\rho v_{\textbf {n}}{\textbf {H}}{{{\textbf {V}}}}\cdot {{\textbf {V}}}d\sigma -\int _{\Gamma (t)}v_{\textbf {n}}{\textbf {H}}{\textbf {J}}_\rho \cdot {{\textbf {V}}}d\sigma \\  &\qquad +\int _{\Gamma (t)}\mu \nabla _\Gamma \varphi \cdot {{\textbf {V}}}d\sigma -2\int _{\Gamma (t)}\nu (\varphi )v_{\textbf {n}}{\textbf {H}}: \nabla _\Gamma {{\textbf {V}}}d\sigma +\frac{1}{2}\int _{\Gamma (t)}\rho \nabla _\Gamma (v_{\textbf {n}})^2\cdot {{\textbf {V}}}d\sigma \\  &\qquad -\int _{\Gamma (t)}\rho \partial ^\circ _t\widehat{{\textbf {u}}}\cdot {{\textbf {V}}}-((\rho \widehat{{\textbf {u}}}-{\textbf {J}}_\rho )\cdot \nabla _\Gamma )\widehat{{\textbf {u}}}\cdot {{\textbf {V}}}d\sigma \\&\qquad -\int _{\Gamma (t)}\rho v_{\textbf {n}}{\textbf {H}}\widehat{{\textbf {u}}}\cdot {{\textbf {V}}}d\sigma -2\int _{\Gamma (t)}\nu (\varphi ){\mathcal {E}}_S(\widehat{{\textbf {u}}}):{\mathcal {E}}_S({{\textbf {V}}})d\sigma . \end{aligned}$$

From (5.3)$_3$, multiplying by $\mu$, integrating by parts where necessary, we obtain (see also (4.9) with ${\textbf {v}}={{\textbf {V}}}+\widehat{{\textbf {u}}}+{{\textbf {V}}}_{\textbf {n}}$)

5.8 $$\begin{aligned}&\frac{d}{dt}\left( \frac{1}{2}\Vert \nabla _\Gamma \varphi \Vert ^2+\int _{\Gamma (t)}\Psi (\varphi )d\sigma \right) +\int _{\Gamma (t)}\vert \nabla _\Gamma \mu \vert ^2d\sigma +\int _{\Gamma (t)}\nabla _\Gamma \varphi \cdot (\widehat{{\textbf {u}}}+{{\textbf {V}}}) \ \mu d\sigma \nonumber \\&\quad =\frac{1}{2}\int _{\Gamma (t)}\vert \nabla _\Gamma \varphi \vert ^2H v_{\textbf {n}}d\sigma -\int _{\Gamma (t)}\nabla _\Gamma \varphi \cdot {\mathcal {E}}_S({{\textbf {V}}}_{\textbf {n}})\nabla _\Gamma \varphi d\sigma +\int _{\Gamma (t)}\Psi (\varphi )H v_{\textbf {n}}d\sigma , \end{aligned}$$

since $\nabla _\Gamma \varphi \cdot {{\textbf {V}}}_{\textbf {n}}=0$. Summing up the two identities above, and setting

5.9 $$\begin{aligned} E_{tot}(\rho ,{{\textbf {V}}},\varphi ):=\frac{1}{2}\int _{\Gamma (t)}\rho \left| {{\textbf {V}}}\right| ^2d\sigma + \frac{1}{2}\Vert \nabla _\Gamma \varphi \Vert ^2+\int _{\Gamma (t)}\Psi (\varphi )d\sigma , \end{aligned}$$

we get the following energy identity

5.10 $$\begin{aligned}&\frac{d}{dt}E_{tot}(\rho ,{{\textbf {V}}},\varphi )+2\int _{\Gamma (t)}\nu (\varphi )\left| {\mathcal {E}}_S({{\textbf {V}}})\right| ^2d\sigma +\int _{\Gamma (t)}\vert \nabla _\Gamma \mu \vert ^2d\sigma \nonumber \\&\quad =\frac{1}{2}\int _{\Gamma (t)}\rho \left| {{\textbf {V}}}\right| ^2H v_{\textbf {n}}d\sigma -\int _{\Gamma (t)}\rho ({{\textbf {V}}}\cdot \nabla _\Gamma )\widehat{{\textbf {u}}}\cdot {{\textbf {V}}}d\sigma \nonumber \\&\qquad -\int _{\Gamma (t)}\rho v_{\textbf {n}}{\textbf {H}}{{{\textbf {V}}}}\cdot {{\textbf {V}}}d\sigma -\int _{\Gamma (t)}v_{\textbf {n}}{\textbf {H}}{\textbf {J}}_\rho \cdot {{\textbf {V}}}d\sigma \nonumber \\&\qquad -\int _{\Gamma (t)}\mu \nabla _\Gamma \varphi \cdot \widehat{{\textbf {u}}}d\sigma -2\int _{\Gamma (t)}\nu (\varphi )v_{\textbf {n}}{\textbf {H}}: \nabla _\Gamma {{\textbf {V}}}d\sigma \nonumber \\&\qquad +\frac{1}{2}\int _{\Gamma (t)}\rho \nabla _\Gamma (v_{\textbf {n}})^2\cdot {{\textbf {V}}}d\sigma \nonumber \\&\qquad -\int _{\Gamma (t)}\rho \partial ^\circ _t\widehat{{\textbf {u}}}\cdot {{\textbf {V}}}-((\rho \widehat{{\textbf {u}}}-{\textbf {J}}_\rho )\cdot \nabla _\Gamma )\widehat{{\textbf {u}}}\cdot {{\textbf {V}}}d\sigma -\int _{\Gamma (t)}\rho v_{\textbf {n}}{\textbf {H}}\widehat{{\textbf {u}}}\cdot {{\textbf {V}}}d\sigma \nonumber \\  &\qquad -2\int _{\Gamma (t)}\nu (\varphi ){\mathcal {E}}_S(\widehat{{\textbf {u}}}):{\mathcal {E}}_S({{\textbf {V}}})d\sigma \nonumber \\&\qquad +\frac{1}{2}\int _{\Gamma (t)}\vert \nabla _\Gamma \varphi \vert ^2H v_{\textbf {n}}d\sigma -\int _{\Gamma (t)}\nabla _\Gamma \varphi \cdot {\mathcal {E}}_S({{\textbf {V}}}_{\textbf {n}})\nabla _\Gamma \varphi d\sigma +\int _{\Gamma (t)}\Psi (\varphi )H v_{\textbf {n}}d\sigma , \end{aligned}$$

since, due to $\textrm{div}_\Gamma {{\textbf {V}}}=0$, integrating by parts, $\frac{1}{2}\int _{\Gamma (t)}\nabla _\Gamma (v_{\textbf {n}})^2\cdot {{\textbf {V}}}d\sigma =0$. Now we recall that, thanks to the regularity of the flow map (see (2.2)-(2.3)), we have

5.11 $$\begin{aligned}&\left\| {\textbf {H}}\right\| _{{{\textbf {C}}}^4(\Gamma (t))}+\left\| {{\textbf {V}}}_{\textbf {n}}\right\| _{{{\textbf {C}}}^4(\Gamma (t))}+\left\| {\textbf {A}}\right\| _{C^4(\Gamma (t))} \\&\quad +\left\| {\textbf {A}}^{-1}\right\| _{C^4(\Gamma (t))}+\left\| \partial ^\circ _t{\textbf {A}}^{-1}\right\| _{C^3(\Gamma (t))}+\left\| \widehat{{\textbf {u}}}\right\| _{C^4_{{\textbf {W}}^{3,p}}}\le C(T),\quad \forall t\in [0,T],\quad \forall p\in [2,+\infty ),\nonumber \end{aligned}$$

and we recall that, for $p>2$, ${\textbf {W}}^{3,p}(\Gamma (t))\hookrightarrow {\textbf {W}}^{2,\infty }(\Gamma (t))$. We can thus estimate all the terms accordingly. In particular, we have, recalling the definition of ${\textbf {J}}_\rho$, and by Hölder’s and Young’s inequality,

$$\begin{aligned}&\left| \frac{1}{2}\int _{\Gamma (t)}\rho \left| {{\textbf {V}}}\right| ^2H v_{\textbf {n}}d\sigma -\int _{\Gamma (t)}\rho ({{\textbf {V}}}\cdot \nabla _\Gamma )\widehat{{\textbf {u}}}\cdot {{\textbf {V}}}d\sigma -\int _{\Gamma (t)}\rho v_{\textbf {n}}{\textbf {H}}{{{\textbf {V}}}}\cdot {{\textbf {V}}}d\sigma -\int _{\Gamma (t)}v_{\textbf {n}}{\textbf {H}}{\textbf {J}}_\rho \cdot {{\textbf {V}}}d\sigma \right| \\  &\quad \le C\left\| Hv_{\textbf {n}}\right\| _{L^\infty (\Gamma (t))}\left\| {{\textbf {V}}}\right\| ^2+C\left\| {{\textbf {V}}}\right\| ^2\left\| \nabla _\Gamma \widehat{{\textbf {u}}}\right\| _{{\textbf {L}}^\infty (\Gamma (t))}+C\left\| v_{\textbf {n}}{\textbf {H}}\right\| _{{\textbf {L}}^\infty (\Gamma (t))}\left\| {{\textbf {V}}}\right\| ^2\\  &\qquad +C\left\| v_{\textbf {n}}{\textbf {H}}\right\| _{{\textbf {L}}^\infty (\Gamma (t))}\left\| \nabla _\Gamma \mu \right\| \left\| {{\textbf {V}}}\right\| \\  &\quad \le C(T)\left\| {{\textbf {V}}}\right\| ^2+\frac{1}{4} \left\| \nabla _\Gamma \mu \right\| ^2. \end{aligned}$$

Then, after an integration by parts, recalling $\textrm{div}_\Gamma \widehat{{\textbf {u}}}=Hv_{\textbf {n}}$, we infer

$$\begin{aligned} \left| -\int _{\Gamma (t)}\mu \nabla _\Gamma \varphi \cdot \widehat{{\textbf {u}}}d\sigma \right| \le \left| \int _{\Gamma (t)}\varphi \nabla _\Gamma \mu \cdot \widehat{{\textbf {u}}}d\sigma \right| + \left| \int _{\Gamma (t)}\mu \varphi Hv_{\textbf {n}}d\sigma \right| . \end{aligned}$$

By following the same argument leading to (4.13), recalling that $$\left| \varphi \right| <1$$ on $$[0,T_m)$$ by assumption, we deduce that

5.12 $$\begin{aligned} \left| \overline{\mu }\right| \le C(T)(1+\left\| \nabla _\Gamma \mu \right\| ) \end{aligned}$$

so that, since $\left| \Gamma (t)\right| \equiv \left| \Gamma _0\right|$,

$$\begin{aligned} \left| -\int _{\Gamma (t)}\mu \nabla _\Gamma \varphi \cdot \widehat{{\textbf {u}}}d\sigma \right|&\le C\left\| \varphi \right\| _{L^\infty (\Gamma (t))}\left\| \mu \right\| \left\| \widehat{{\textbf {u}}}\right\| _{{\textbf {L}}^2(\Gamma (t))} \le C(T)(1+\left\| \nabla _\Gamma \mu \right\| )\le C(T)\\&\quad +\frac{1}{4} \left\| \nabla _\Gamma \mu \right\| ^2. \end{aligned}$$

Then, we have, recalling again the definition of ${\textbf {J}}_\rho$ and Young’s inequality,

$$\begin{aligned}&\left| -2\int _{\Gamma (t)}\nu (\varphi )v_{\textbf {n}}{\textbf {H}}:\nabla _\Gamma {{\textbf {V}}}d\sigma -\int _{\Gamma (t)}\rho \partial ^\circ _t\widehat{{\textbf {u}}}\cdot {{\textbf {V}}}-((\rho \widehat{{\textbf {u}}}-{\textbf {J}}_\rho )\cdot \nabla _\Gamma )\widehat{{\textbf {u}}}\cdot {{\textbf {V}}}d\sigma \right. \\  &\left. \qquad +\frac{1}{2}\int _{\Gamma (t)}\rho \nabla _\Gamma (v_{\textbf {n}})^2\cdot {{\textbf {V}}}d\sigma -\int _{\Gamma (t)}\rho v_{\textbf {n}}{\textbf {H}}\widehat{{\textbf {u}}}\cdot {{\textbf {V}}}d\sigma -2\int _{\Gamma (t)}\nu (\varphi ){\mathcal {E}}_S(\widehat{{\textbf {u}}}):{\mathcal {E}}_S({{\textbf {V}}})d\sigma \right| \\  &\quad \le C\left\| v_{\textbf {n}}{\textbf {H}}\right\| _{{\textbf {L}}^\infty (\Gamma (t))}\left\| \nabla _\Gamma {{\textbf {V}}}\right\| +C\left\| \partial ^\circ _t\widehat{{\textbf {u}}}\right\| _{{\textbf {L}}^\infty (\Gamma (t))}\left\| {{\textbf {V}}}\right\| +C\left\| \widehat{{\textbf {u}}}\right\| _{{\textbf {L}}^\infty (\Gamma (t))}\left\| \nabla _\Gamma \widehat{{\textbf {u}}}\right\| _{{\textbf {L}}^\infty (\Gamma (t))}\left\| {{\textbf {V}}}\right\| \\  &\qquad +C\left\| \nabla _\Gamma \mu \right\| \left\| \nabla _\Gamma \widehat{{\textbf {u}}}\right\| _{{\textbf {L}}^\infty (\Gamma (t))}\left\| {{\textbf {V}}}\right\| +C\left\| \nabla _\Gamma (v_{\textbf {n}})^2\right\| _{{\textbf {L}}^\infty (\Gamma (t))}\left\| {{\textbf {V}}}\right\| \\  &\qquad +C\left\| v_{\textbf {n}}{\textbf {H}}\widehat{{\textbf {u}}}\right\| _{{\textbf {L}}^\infty (\Gamma (t))}\left\| {{\textbf {V}}}\right\| +C\left\| \widehat{{\textbf {u}}}\right\| _{{\textbf {W}}^{1,\infty }(\Gamma (t))}\left\| \nabla _\Gamma {{\textbf {V}}}\right\| \\  &\quad \le C(T)(1+\left\| {{\textbf {V}}}\right\| ^2)+\frac{1}{4} \left\| \nabla _\Gamma \mu \right\| ^2+{\nu _*}\left\| {\mathcal {E}}_S({{\textbf {V}}})\right\| ^2, \end{aligned}$$

where in the last estimate we applied the (evolving) Korn’s inequality (2.7). By similar estimates, we infer

$$\begin{aligned}&\left| \frac{1}{2}\int _{\Gamma (t)}\vert \nabla _\Gamma \varphi \vert ^2H v_{\textbf {n}}d\sigma -\int _{\Gamma (t)}\nabla _\Gamma \varphi {\mathcal {E}}_S({{\textbf {V}}}_{\textbf {n}})\nabla _\Gamma ^T\varphi d\sigma +\int _{\Gamma (t)}\Psi (\varphi )H v_{\textbf {n}}d\sigma \right| \\  &\quad \le C(T)\left( \left\| \nabla _\Gamma \varphi \right\| ^2+\int _{\Gamma (t)}(\Psi (\varphi )+\widehat{C})d\sigma \right) , \end{aligned}$$

where $\widehat{C}>0$ is such that $\Psi +\widehat{C}>0$. By redefining $E_{tot}$ as to be

5.13 $$\begin{aligned} \widehat{E}_{tot}(t):=\frac{1}{2}\int _{\Gamma (t)}\rho \left| {{\textbf {V}}}\right| ^2d\sigma + \frac{1}{2}\Vert \nabla _\Gamma \varphi \Vert ^2+\int _{\Gamma (t)}(\Psi (\varphi )+\widehat{C})d\sigma , \end{aligned}$$

and recalling that $\nu (\varphi )\ge \nu _*$ by assumption, we deduce from all the estimates above put together in (5.10), that

5.14 $$\begin{aligned} \frac{d}{dt}\widehat{E}_{tot}(t)+\nu _*\left\| {\mathcal {E}}_S({{\textbf {V}}})\right\| ^2+\frac{1}{4}\left\| \nabla _\Gamma \mu \right\| ^2\le C(T)(1+\widehat{E}_{tot}(t)), \quad \forall t\in [0,T_m). \end{aligned}$$

Since $T_m\le T$, this allows to deduce by the Gronwall lemma and (5.12) (by Poincaré’s inequality), that

5.15 $$\begin{aligned} \left\| {{\textbf {V}}}\right\| _ {L^\infty _{{\textbf {L}}^2_\sigma (T_m)}}+\left\| {{\textbf {V}}}\right\| _{L^2_{{\textbf {H}}^1_\sigma (T_m)}}+\left\| \varphi \right\| _{L^\infty _{H^1(T_m)}}+\left\| \mu \right\| _{L^2_{H^1(T_m)}}\le C(T). \end{aligned}$$

Since $\varphi$ satisfies problem (4.1) with ${\textbf {v}}={{\textbf {V}}}+\widehat{{\textbf {u}}}+{{\textbf {V}}}_{\textbf {n}}$, the regularity on the initial datum $\varphi _0$, together with estimates (5.11) and (5.15) allow to apply Theorem 4.1 and deduce, by uniqueness of the solution to (4.1), that

5.16 $$\begin{aligned} \left\| \mu \right\| _{H^1_{H^{-1}(T_m)}}+\left\| \mu \right\| _{L^\infty _{H^1(T_m)}}+\left\| \mu \right\| _{L^2_{H^3(T_m)}}+\left\| \varphi \right\| _{L^\infty _{H^3(T_m)}}+\left\| \varphi \right\| _{H^1_{H^1(T_m)}}&\le C(T,\left\| {\textbf {u}}\right\| _{L^2_{{\textbf {H}}^1(T_m) }})\nonumber \\&\le C(T), \end{aligned}$$

as well as there exists $\delta =\delta (T)\in (0,1)$ such that

5.17 $$\begin{aligned} \sup _{t\in [0,T_m)}\left\| \varphi \right\| _{L^\infty (\Gamma (t))}\le 1-\delta . \end{aligned}$$

*Higher-order estimates. *To conclude the contradiction argument, we need to show some higher-order estimates on the velocity ${{\textbf {V}}}$. First, we notice that (5.3)$_1$, satisfied by $({{\textbf {V}}},p,\varphi )$, can be rewritten as

$$\begin{aligned}&-2{\textbf {P}}\textrm{div}_\Gamma (\nu (\varphi ){\mathcal {E}}_S({{\textbf {V}}}))+\omega {{\textbf {V}}}+\nabla _\Gamma \pi \\  &\quad ={{\textbf {f}}}:= \omega {{\textbf {V}}}-\rho {\textbf {P}}\partial ^\circ _t{{\textbf {V}}}-((\rho {{\textbf {V}}}+{\textbf {J}}_\rho )\cdot \nabla _\Gamma ){{\textbf {V}}}-\rho (\widehat{{\textbf {u}}}\cdot \nabla _\Gamma ){{\textbf {V}}}-\rho ({{\textbf {V}}}\cdot \nabla _\Gamma )\widehat{{\textbf {u}}}-\rho v_{\textbf {n}}{\textbf {H}}{{\textbf {V}}}-v_{\textbf {n}}{\textbf {H}}{\textbf {J}}_\rho \\  &\qquad -{\textbf {P}}\textrm{div}_\Gamma (\nabla _\Gamma \varphi \otimes \nabla _\Gamma \varphi )+2{\textbf {P}}\textrm{div}_\Gamma (\nu (\varphi )v_{\textbf {n}}{\textbf {H}}) +\frac{\rho }{2}\nabla _\Gamma ^T(v_{\textbf {n}})^2-\rho {\textbf {P}}\partial ^\circ _t\widehat{{\textbf {u}}} -((\rho \widehat{{\textbf {u}}}+{\textbf {J}}_\rho )\cdot \nabla _\Gamma )\widehat{{\textbf {u}}}\\  &\qquad -\rho v_{\textbf {n}}{\textbf {H}}\widehat{{\textbf {u}}}+2{\textbf {P}}\textrm{div}_\Gamma (\nu (\varphi ){\mathcal {E}}_S(\widehat{{\textbf {u}}})), \end{aligned}$$

where $\omega >0$ is arbitrary. This means that the corresponding weak formulation coincides with the one in [4, Lemma 7.4]. Therefore, we can apply [4, Lemma 7.4] and infer that

5.18 $$\begin{aligned} \left\| {{\textbf {V}}}(t)\right\| _{{{\textbf {H}}^2}(t)}&\le C(T,\omega )(1+\left\| \varphi (t)\right\| _{L_{W^{1,\infty }(\Gamma (t))}})\left\| {{\textbf {f}}}(t)\right\| _{{{\textbf {L}}^2}(\Gamma (t))}\nonumber \\&\le C(T,\omega )\left\| {{\textbf {f}}}(t)\right\| _{{{\textbf {L}}^2}(\Gamma (t))}, \end{aligned}$$

for almost any $t\in (0,T_m)$, recalling the embedding (with a uniform-in-time embedding constant) $H^3(\Gamma (t))\hookrightarrow W^{1,\infty }(\Gamma (t))$ and (5.16). Now we need to estimate the right-hand side. We have, recalling (5.15),

$$ \left\| \omega {{\textbf {V}}}-\rho {\textbf {P}}\partial ^\circ _t{{\textbf {V}}}\right\| \le C\left\| {{\textbf {V}}}\right\| +\rho ^*\left\| \partial ^\circ _t{{\textbf {V}}}\right\| )\le C(T)+\rho ^*\left\| {\textbf {P}}\partial ^\circ _t{{\textbf {V}}}\right\| , $$

as well as, by Sobolev-Gagliardo-Nirenberg’s and Young’s inequalities, and (2.7),

5.19 $$\begin{aligned} \left\| \rho ({{\textbf {V}}}\cdot \nabla _\Gamma ){{\textbf {V}}}\right\|&\le \rho ^*\left\| {{\textbf {V}}}\right\| _{{\textbf {L}}^4(\Gamma (t))}\left\| \nabla _\Gamma {{\textbf {V}}}\right\| _{{\textbf {L}}^4(\Gamma (t))}\nonumber \\&\le C(T)\left\| {{\textbf {V}}}\right\| ^\frac{1}{2}\left\| {{\textbf {V}}}\right\| _{{\textbf {H}}^1(\Gamma (t))}\left\| {{\textbf {V}}}\right\| _{{\textbf {H}}^2(\Gamma (t))}^\frac{1}{2}\nonumber \\  &\le C(T)\left\| {{\textbf {V}}}\right\| ^\frac{1}{2}(\left\| {{\textbf {V}}}\right\| +\left\| {\mathcal {E}}_S({{\textbf {V}}})\right\| )\left\| {{\textbf {V}}}\right\| _{{\textbf {H}}^2(\Gamma (t))}^\frac{1}{2}\nonumber \\  &\le C(T)(1+\left\| {\mathcal {E}}_S({{\textbf {V}}})\right\| )\left\| {{\textbf {V}}}\right\| _{{\textbf {H}}^2(\Gamma (t))}^\frac{1}{2}\nonumber \\  &\le C(T)(1+\left\| {\mathcal {E}}_S({{\textbf {V}}})\right\| ^2)+\frac{1}{2C(T,\omega )}\left\| {{\textbf {V}}}\right\| _{{\textbf {H}}^2(\Gamma (t))}. \end{aligned}$$

Then, by Agmon’s inequality (2.9) and (5.15)-(5.16), we infer, recalling the definition of ${\textbf {J}}_\rho$,

5.20 $$\begin{aligned} \left\| ({\textbf {J}}_\rho \cdot \nabla _\Gamma ){{\textbf {V}}}\right\|&\le C\left\| \nabla _\Gamma \mu \right\| _{{\textbf {L}}^\infty (\Gamma (t))}\left\| \nabla _\Gamma {{\textbf {V}}}\right\| \nonumber \\&\le C(T)\left\| \nabla _\Gamma \mu \right\| ^\frac{1}{2}\left\| \mu \right\| _{H^3(\Gamma (t))}^\frac{1}{2}(\left\| {{\textbf {V}}}\right\| +\left\| {\mathcal {E}}_S({{\textbf {V}}})\right\| )\nonumber \\&\le C(T)\left\| \mu \right\| _{H^3(\Gamma (t))}^\frac{1}{2}(1+\left\| {\mathcal {E}}_S({{\textbf {V}}})\right\| ). \end{aligned}$$

Furthermore, again by (5.16), we deduce

$$\begin{aligned} \left\| {\textbf {P}}\textrm{div}_\Gamma (\nabla _\Gamma \varphi \otimes \nabla _\Gamma \varphi )\right\|&\le C\left\| \varphi \right\| _{H^2(\Gamma (t))}\left\| \nabla _\Gamma \varphi \right\| _{{\textbf {L}}^\infty (\Gamma (t))} \le C(T), \end{aligned}$$

again by the embedding $H^3(\Gamma (t))\hookrightarrow W^{1,\infty }(\Gamma (t))$. Analogously, we have

$$\begin{aligned}&\left\| 2{\textbf {P}}\textrm{div}_\Gamma (\nu (\varphi )v_{\textbf {n}}{\textbf {H}})+2{\textbf {P}}\textrm{div}_\Gamma (\nu (\varphi ){\mathcal {E}}_S(\widehat{{\textbf {u}}}))\right\| \\  &\le C(1+\left\| \varphi \right\| _{H^1(\Gamma (t))})(\left\| v_{\textbf {n}}{\textbf {H}}\right\| _{{\textbf {W}}^{1,\infty }(\Gamma (t))}+\left\| \widehat{{\textbf {u}}}\right\| _{{\textbf {W}}^{2,\infty }(\Gamma (t))})\le C(T). \end{aligned}$$

Proceeding in estimating the lower-order terms in ${{\textbf {f}}}$, we have, recalling (5.11), (5.15)-(5.16) and the definition of ${\textbf {J}}_\rho$,

$$\begin{aligned}&\left\| -\rho ({{\textbf {V}}}\cdot \nabla _\Gamma )\widehat{{\textbf {u}}}-\rho v_{\textbf {n}}{\textbf {H}}{{\textbf {V}}}-v_{\textbf {n}}{\textbf {H}}{\textbf {J}}_\rho +\frac{\rho }{2}\nabla _\Gamma ^T(v_{\textbf {n}})^2-\rho {\textbf {P}}\partial ^\circ _t\widehat{{\textbf {u}}} -((\rho \widehat{{\textbf {u}}}+{\textbf {J}}_\rho )\cdot \nabla _\Gamma )\widehat{{\textbf {u}}}-\rho v_{\textbf {n}}{\textbf {H}}\widehat{{\textbf {u}}}\right\| \\  &\quad \le C(T)(\left\| {{\textbf {V}}}\right\| \left\| \nabla _\Gamma \widehat{{\textbf {u}}}\right\| _{{\textbf {L}}^\infty (\Gamma (t))}+\left\| v_{\textbf {n}}{\textbf {H}}\right\| _{{\textbf {L}}^\infty (\Gamma (t))}(\left\| {{\textbf {V}}}\right\| +\left\| \nabla _\Gamma \mu \right\| )+\left\| \nabla _\Gamma (v_{\textbf {n}})^2\right\| _{{\textbf {L}}^\infty (\Gamma (t))}\\  &\qquad +\left\| \partial ^\circ _t\widehat{{\textbf {u}}}\right\| _{{\textbf {L}}^\infty (\Gamma (t))}+(\left\| \widehat{{\textbf {u}}}\right\| _{{\textbf {L}}^\infty (\Gamma (t))}+\left\| \nabla _\Gamma \mu \right\| )\left\| \nabla _\Gamma \widehat{{\textbf {u}}}\right\| _{{\textbf {L}}^\infty (\Gamma (t))}+\left\| v_{\textbf {n}}{\textbf {H}}\right\| _{{\textbf {L}}^\infty (\Gamma (t))})\\  &\quad \le C(T). \end{aligned}$$

Therefore, we can conclude from (5.18), elevating to the square, that

5.21 $$\begin{aligned}&\left\| {{\textbf {V}}}\right\| _{{\textbf {H}}^2(\Gamma (t))}^2\le C(T,\omega )(1+\left\| \partial ^\circ _t{{\textbf {V}}}\right\| ^2+\left\| {\mathcal {E}}_S({{\textbf {V}}})\right\| ^4+\left\| \mu \right\| _{H^3(\Gamma (t))}(1+\left\| {\mathcal {E}}_S({{\textbf {V}}})\right\| ^2)). \end{aligned}$$

We recall that, thanks to (5.11) and (5.15),

5.22 $$\begin{aligned} \left\| \partial ^\circ _t{{\textbf {V}}}\right\| \le C(T)(1+\left\| \partial _t^*{{\textbf {V}}}\right\| ),\quad \left\| \partial _t^*{{\textbf {V}}}\right\| \le C(T)(1+\left\| \partial ^\circ _t{{\textbf {V}}}\right\| ), \end{aligned}$$

so that

5.23 $$\begin{aligned}&\left\| {{\textbf {V}}}\right\| _{{\textbf {H}}^2(\Gamma (t))}^2\le C(T,\omega )(1+\left\| \partial _t^*{{\textbf {V}}}\right\| ^2+\left\| {\mathcal {E}}_S({{\textbf {V}}})\right\| ^4+\left\| \mu \right\| _{H^3(\Gamma (t))}(1+\left\| {\mathcal {E}}_S({{\textbf {V}}})\right\| ^2)). \end{aligned}$$

Let us now multiply (5.3)$_1$ by $\partial _t^*{{\textbf {V}}}$ and integrate over $\Gamma (t)$. Recall that $\textrm{div}_\Gamma (\partial _t^*{{\textbf {V}}})=0$ and ${\textbf {P}}\partial _t^*{{\textbf {V}}}=\partial _t^*{{\textbf {V}}}$. We cannot simply use $\partial ^\circ _t{{\textbf {V}}}$, since it does not preserve the divergence-free property. The most involved part is related to the following term

$$-\int _{\Gamma (t)}\textrm{div}_\Gamma (\nu (\varphi ){\mathcal {E}}_S({{\textbf {V}}}))\cdot \partial _t^*{{\textbf {V}}}d\sigma .$$

Indeed, from (2.14) and Remark 2.3 we infer

5.24 $$\begin{aligned}&\frac{d}{dt}\int _{\Gamma (t)}\nu (\varphi ) \left| {\mathcal {E}}_S({{\textbf {V}}})\right| ^2d\sigma =\int _{\Gamma }\nu '(\varphi )\partial ^\circ _t\varphi \left| {\mathcal {E}}_S({{\textbf {V}}})\right| ^2d\sigma \nonumber -2\int _\Gamma \textrm{div}_\Gamma (\nu (\varphi ){\mathcal {E}}_S( {{\textbf {V}}}))\cdot \partial ^\circ _t{{\textbf {V}}}d\sigma \nonumber \\&\quad +4\int _{\Gamma }\nu (\varphi )\mathcal {S}(({\textbf {n}}\otimes {\textbf {n}})\widehat{\nabla }_\Gamma {{\textbf {V}}}_{\textbf {n}})\widehat{{\mathcal {E}}}_S({{\textbf {V}}}):{\mathcal {E}}_S({{\textbf {V}}})d\sigma +4\int _{\Gamma }\nu (\varphi ){{\mathcal {E}}}_S({{\textbf {V}}}):\widehat{{\mathcal {E}}}_S({{\textbf {V}}})\mathcal {S}(({\textbf {n}}\otimes {\textbf {n}})\widehat{\nabla }_\Gamma {{\textbf {V}}}_{\textbf {n}})d\sigma \nonumber \\&\quad -2\int _\Gamma \nu (\varphi )\mathcal {S}((\nabla _\Gamma {{\textbf {V}}})\nabla _\Gamma {{\textbf {V}}}_{\textbf {n}}):{\mathcal {E}}_S({{\textbf {V}}})d\sigma +\int _{\Gamma (t)}\nu (\varphi ) \left| {\mathcal {E}}_S({{\textbf {V}}})\right| ^2\ \textrm{div}_\Gamma {{\textbf {V}}}_{\textbf {n}}d\sigma . \end{aligned}$$

The difficulty is that we only control $\partial _t^*{{\textbf {V}}}$ (or ${\textbf {P}}\partial ^\circ _t{{\textbf {V}}}$) and not the entire $\partial ^\circ _t{{\textbf {V}}}$. Recalling (2.6), we perform the following splitting

$$\begin{aligned}&-2\int _\Gamma \textrm{div}_\Gamma (\nu (\varphi ){\mathcal {E}}_S( {{\textbf {V}}}))\cdot \partial _t^*{{\textbf {V}}}d\sigma \\  &\quad =-2\int _\Gamma \textrm{div}_\Gamma (\nu (\varphi ){\mathcal {E}}_S( {{\textbf {V}}}))\cdot \partial ^\circ _t{{\textbf {V}}}d\sigma +2\int _\Gamma \textrm{div}_\Gamma (\nu (\varphi ){\mathcal {E}}_S( {{\textbf {V}}}))\cdot ({\textbf {A}}(\partial ^\circ _t{\textbf {A}}^{-1}){{\textbf {V}}})d\sigma . \end{aligned}$$

In the end we have:

5.25 $$\begin{aligned}&\frac{d}{dt}\int _{\Gamma (t)}\nu (\varphi ) \left| {\mathcal {E}}_S({{\textbf {V}}})\right| ^2d\sigma +\int _{\Gamma (t)}\rho \left| \partial _t^*{{\textbf {V}}}\right| ^2d\sigma \nonumber \\  &\quad =-\int _{\Gamma (t)}\rho \partial _t^*{{\textbf {V}}}\cdot {\textbf {A}}(\partial ^\circ _t{\textbf {A}}^{-1}){{\textbf {V}}}d\sigma +\int _{\Gamma }\nu '(\varphi )\partial ^\circ _t\varphi \left| {\mathcal {E}}_S({{\textbf {V}}})\right| ^2d\sigma \nonumber \\  &\qquad -2\int _\Gamma \textrm{div}_\Gamma (\nu (\varphi ){\mathcal {E}}_S( {{\textbf {V}}}))\cdot {\textbf {A}}(\partial ^\circ _t{\textbf {A}}^{-1}){{\textbf {V}}}d\sigma \nonumber \\&\qquad +4\int _{\Gamma }\nu (\varphi )\mathcal {S}(({\textbf {n}}\otimes {\textbf {n}})\widehat{\nabla }_\Gamma {{\textbf {V}}}_{\textbf {n}})\widehat{{\mathcal {E}}}_S({{\textbf {V}}}):{\mathcal {E}}_S({{\textbf {V}}})d\sigma \nonumber \\&\qquad +4\int _{\Gamma }\nu (\varphi ){{\mathcal {E}}}_S({{\textbf {V}}}):\widehat{{\mathcal {E}}}_S({{\textbf {V}}})\mathcal {S}(({\textbf {n}}\otimes {\textbf {n}})\widehat{\nabla }_\Gamma {{\textbf {V}}}_{\textbf {n}})d\sigma \nonumber \\  &\qquad -2\int _\Gamma \nu (\varphi )\mathcal {S}((\nabla _\Gamma {{\textbf {V}}})\nabla _\Gamma {{\textbf {V}}}_{\textbf {n}}):{\mathcal {E}}_S({{\textbf {V}}})d\sigma +\int _{\Gamma (t)}\nu (\varphi ) \left| {\mathcal {E}}_S({{\textbf {V}}})\right| ^2\ \textrm{div}_\Gamma {{\textbf {V}}}_{\textbf {n}}d\sigma \nonumber \\&\qquad -\int _{\Gamma (t)}((\rho {{\textbf {V}}}+{\textbf {J}}_\rho )\cdot \nabla _\Gamma ){{\textbf {V}}}\cdot \partial _t^*{{\textbf {V}}}d\sigma -\int _{\Gamma (t)}\rho (\widehat{{\textbf {u}}}\cdot \nabla _\Gamma ){{\textbf {V}}}\cdot \partial _t^*{{\textbf {V}}}d\sigma \nonumber \\&\qquad +\int _{\Gamma (t)}\left( -\rho ({{\textbf {V}}}\cdot \nabla _\Gamma )\widehat{{\textbf {u}}}-\rho v_{\textbf {n}}{\textbf {H}}{{\textbf {V}}}-v_{\textbf {n}}{\textbf {H}}{\textbf {J}}_\rho \right) \cdot \partial _t^*{{\textbf {V}}}d\sigma \nonumber \\&\qquad +\int _{\Gamma (t)}\left( -{\textbf {P}}\textrm{div}_\Gamma (\nabla _\Gamma \varphi \otimes \nabla _\Gamma \varphi )+2{\textbf {P}}\textrm{div}_\Gamma (\nu (\varphi )v_{\textbf {n}}{\textbf {H}})\right) \cdot \partial _t^*{{\textbf {V}}}d\sigma \nonumber \\&\qquad +\int _{\Gamma (t)}\left( \frac{\rho }{2}\nabla _\Gamma (v_{\textbf {n}})^2-\rho {\textbf {P}}\partial ^\circ _t\widehat{{\textbf {u}}} -((\rho \widehat{{\textbf {u}}}+{\textbf {J}}_\rho )\cdot \nabla _\Gamma )\widehat{{\textbf {u}}}-\rho v_{\textbf {n}}{\textbf {H}}\widehat{{\textbf {u}}}+2{\textbf {P}}\textrm{div}_\Gamma (\nu (\varphi ){\mathcal {E}}_S(\widehat{{\textbf {u}}}))\right) \nonumber \\&\qquad \cdot \partial _t^*{{\textbf {V}}}d\sigma . \end{aligned}$$

We now estimate all the terms appearing here. First, we have from (5.11) (see also (2.2)) and (5.15),

$$\begin{aligned} \left| -\int _{\Gamma (t)}\rho \partial _t^*{{\textbf {V}}}\cdot {\textbf {A}}(\partial ^\circ _t{\textbf {A}}^{-1}){{\textbf {V}}}d\sigma \right| \le C(T)\left\| \partial _t^*{{\textbf {V}}}\right\| \left\| {{\textbf {V}}}\right\| \le C(T)+\frac{\rho _*}{16}\left\| \partial _t^*{{\textbf {V}}}\right\| ^2. \end{aligned}$$

Then, recalling (2.7), (2.8), and (5.16),

$$\begin{aligned}&\left| \int _{\Gamma }\nu '(\varphi )\partial ^\circ _t\varphi \left| {\mathcal {E}}_S({{\textbf {V}}})\right| ^2d\sigma \right| \le C\left\| \partial ^\circ _t\varphi \right\| \left\| {\mathcal {E}}_S({{\textbf {V}}})\right\| ^2_{{\textbf {L}}^4((\Gamma (t)))}\\  &\quad \le C\left\| \partial ^\circ _t\varphi \right\| \left\| {\mathcal {E}}_S({{\textbf {V}}})\right\| \left\| {{\textbf {V}}}\right\| _{{\textbf {H}}^2(\Gamma (t))} \le C(T,\epsilon )\left\| \partial ^\circ _t\varphi \right\| ^2\left\| {\mathcal {E}}_S({{\textbf {V}}})\right\| ^2+\epsilon \left\| {{\textbf {V}}}\right\| _{{\textbf {H}}^2(\Gamma (t))}^2, \end{aligned}$$

for some $\epsilon >0$ suitably chosen later on. Then, thanks to (5.11) and (5.16),

$$\begin{aligned}&\left| -2\int _\Gamma \textrm{div}_\Gamma (\nu (\varphi ){\mathcal {E}}_S( {{\textbf {V}}}))\cdot {\textbf {A}}(\partial ^\circ _t{\textbf {A}}^{-1}){{\textbf {V}}}d\sigma \right| \\&\quad \le C\left\| \varphi \right\| _{W^{1,\infty }(\Gamma (t))}\left\| {\mathcal {E}}_S( {{\textbf {V}}})\right\| \left\| {{\textbf {V}}}\right\| +C\left\| {{\textbf {V}}}\right\| _{{\textbf {H}}^2(\Gamma (t))}\left\| {{\textbf {V}}}\right\| \\  &\quad \le C(T)\left\| {\mathcal {E}}_S({{\textbf {V}}})\right\| +C(T)\left\| {{\textbf {V}}}\right\| _{{\textbf {H}}^2(\Gamma (t))}\le C(T,\epsilon )(1+\left\| {\mathcal {E}}_S({{\textbf {V}}})\right\| ^2)+\epsilon \left\| {{\textbf {V}}}\right\| _{{\textbf {H}}^2(\Gamma (t))}^2. \end{aligned}$$

Proceeding in the estimates, recalling (2.7) and (5.11), we have

$$\begin{aligned}&\left| 4\int _{\Gamma }\nu (\varphi )\mathcal {S}(({\textbf {n}}\otimes {\textbf {n}})\widehat{\nabla }_\Gamma {{\textbf {V}}}_{\textbf {n}})\widehat{{\mathcal {E}}}_S({{\textbf {V}}}):{\mathcal {E}}_S({{\textbf {V}}})d\sigma +4\int _{\Gamma }\nu (\varphi ){{\mathcal {E}}}_S({{\textbf {V}}}):\widehat{{\mathcal {E}}}_S({{\textbf {V}}})\mathcal {S}(({\textbf {n}}\otimes {\textbf {n}})\widehat{\nabla }_\Gamma {{\textbf {V}}}_{\textbf {n}})d\sigma \right. \\  &\qquad \left. -2\int _\Gamma \nu (\varphi )\mathcal {S}((\nabla _\Gamma {{\textbf {V}}})\nabla _\Gamma {{\textbf {V}}}_{\textbf {n}}):{\mathcal {E}}_S({{\textbf {V}}})d\sigma +\int _{\Gamma (t)}\nu (\varphi ) \left| {\mathcal {E}}_S({{\textbf {V}}})\right| ^2\ \textrm{div}_\Gamma {{\textbf {V}}}_{\textbf {n}}d\sigma \right| \\  &\quad \le C(T)\left\| {{\textbf {V}}}\right\| _{{\textbf {H}}^1(\Gamma (t))}^2\le C(T)(\left\| {{\textbf {V}}}\right\| ^2+\left\| {\mathcal {E}}_S({{\textbf {V}}})\right\| ^2)\le C(T)(1+\left\| {\mathcal {E}}_S({{\textbf {V}}})\right\| ^2). \end{aligned}$$

Moreover, by similar estimates as in (5.19), we get

$$\begin{aligned} \left| \int _{\Gamma (t)}\rho ({{\textbf {V}}}\cdot \nabla _\Gamma ){{\textbf {V}}}\cdot \partial _t^*{{\textbf {V}}}d\sigma \right|&\le \rho ^*\left\| ({{\textbf {V}}}\cdot \nabla _\Gamma ){{\textbf {V}}}\right\| \left\| \partial _t^*{{\textbf {V}}}\right\| \\  &\le \rho ^*\left\| {{\textbf {V}}}\right\| _{{\textbf {L}}^4(\Gamma (t))}\left\| \nabla _\Gamma {{\textbf {V}}}\right\| _{{\textbf {L}}^4(\Gamma (t))}\left\| \partial _t^*{{\textbf {V}}}\right\| \\  &\le C(T)(1+\left\| {\mathcal {E}}_S({{\textbf {V}}})\right\| )\left\| {{\textbf {V}}}\right\| _{{\textbf {H}}^2(\Gamma (t))}^\frac{1}{2}\left\| \partial _t^*{{\textbf {V}}}\right\| \\  &\le C(T,\epsilon )(1+\left\| {\mathcal {E}}_S({{\textbf {V}}})\right\| ^4)+\frac{\rho _*}{16}\left\| \partial _t^*{{\textbf {V}}}\right\| ^2+\epsilon \left\| {{\textbf {V}}}\right\| _{{\textbf {H}}^2(\Gamma (t))}^2, \end{aligned}$$

and, similarly to (5.20),

$$\begin{aligned}&\left| \int _{\Gamma (t)}({\textbf {J}}_\rho \cdot \nabla _\Gamma ){{\textbf {V}}}\cdot \partial _t^*{{\textbf {V}}}d\sigma \right| \le \left\| ({\textbf {J}}_\rho \cdot \nabla _\Gamma ){{\textbf {V}}}\right\| \left\| \partial _t^*{{\textbf {V}}}\right\| \le C\left\| \nabla _\Gamma \mu \right\| _{{\textbf {L}}^\infty (\Gamma (t))}\left\| \nabla _\Gamma {{\textbf {V}}}\right\| \left\| \partial _t^*{{\textbf {V}}}\right\| \\  &\quad \le C(T)\left\| \nabla _\Gamma \mu \right\| ^\frac{1}{2}\left\| \mu \right\| _{H^3(\Gamma (t))}^\frac{1}{2}(\left\| {{\textbf {V}}}\right\| +\left\| {\mathcal {E}}_S({{\textbf {V}}})\right\| )\left\| \partial _t^*{{\textbf {V}}}\right\| \le C(T)\left\| \mu \right\| _{H^3(\Gamma (t))}(1+\left\| {\mathcal {E}}_S({{\textbf {V}}})\right\| ^2)\\  &\quad +\frac{\rho _*}{16}\left\| \partial _t^*{{\textbf {V}}}\right\| ^2. \end{aligned}$$

We can then proceed in the estimates: recalling that $\textrm{div}_\Gamma \partial _t^*{\textbf {v}}=0$ and ${\textbf {P}}\partial _t^*{\textbf {v}}=\partial _t^*{\textbf {v}}$, as well as (5.4) and (5.16), we get

$$\begin{aligned}&\left| \int _{\Gamma (t)}{\textbf {P}}\textrm{div}_\Gamma (\nabla _\Gamma \varphi \otimes \nabla _\Gamma \varphi )\cdot \partial _t^*{{\textbf {V}}}d\sigma \right| =\left| \int _{\Gamma (t)}\mu \nabla _\Gamma \varphi \cdot \partial _t^*{{\textbf {V}}}d\sigma \right| \le \left| \int _{\Gamma (t)}\nabla _\Gamma \mu \varphi \cdot \partial _t^*{{\textbf {V}}}d\sigma \right| \\  &\quad \le \left\| \nabla _\Gamma \mu \right\| \left\| \varphi \right\| _{L^\infty (\Gamma (t))}\left\| \partial _t^*{{\textbf {V}}}\right\| \le C(T)+\frac{\rho _*}{16}\left\| \partial _t^*{{\textbf {V}}}\right\| ^2. \end{aligned}$$

Concerning the lower order terms, we then observe that, thanks to (2.7), and (5.15), (5.16), and recalling the definition of ${\textbf {J}}_\rho$,

$$\begin{aligned}&\left| -\int _{\Gamma (t)}\rho (\widehat{{\textbf {u}}}\cdot \nabla _\Gamma ){{\textbf {V}}}\cdot \partial _t^*{{\textbf {V}}}d\sigma +\int _{\Gamma (t)}\left( -\rho ({{\textbf {V}}}\cdot \nabla _\Gamma )\widehat{{\textbf {u}}}-\rho v_{\textbf {n}}{\textbf {H}}{{\textbf {V}}}-v_{\textbf {n}}{\textbf {H}}{\textbf {J}}_\rho \right) \cdot \partial _t^*{{\textbf {V}}}d\sigma \right| \\  &\quad \le C(\left\| \widehat{{\textbf {u}}}\right\| _{{\textbf {L}}^\infty (\Gamma (t))}\left\| \nabla _\Gamma {{\textbf {V}}}\right\| \left\| \partial _t^*{{\textbf {V}}}\right\| +\left\| {{\textbf {V}}}\right\| \left\| \nabla _\Gamma \widehat{{\textbf {u}}}\right\| _{{\textbf {L}}^\infty (\Gamma (t))}\left\| \partial _t^*{{\textbf {V}}}\right\| \\  &\qquad +(\left\| {{\textbf {V}}}\right\| +\left\| \nabla _\Gamma \mu \right\| )\left\| v_{\textbf {n}}{\textbf {H}}\right\| _{{\textbf {L}}^\infty (\Gamma (t))}\left\| \partial _t^*{{\textbf {V}}}\right\| )\\  &\quad \le C(T)(1+\left\| {\mathcal {E}}_s({{\textbf {V}}})\right\| ^2)+\frac{\rho _*}{16}\left\| \partial _t^*{{\textbf {V}}}\right\| ^2, \end{aligned}$$

as well as,

$$\begin{aligned}&\left| \int _{\Gamma (t)}\left( \frac{\rho }{2}\nabla _\Gamma (v_{\textbf {n}})^2-\rho {\textbf {P}}\partial ^\circ _t\widehat{{\textbf {u}}} -((\rho \widehat{{\textbf {u}}}+{\textbf {J}}_\rho )\cdot \nabla _\Gamma )\widehat{{\textbf {u}}}-\rho v_{\textbf {n}}{\textbf {H}}\widehat{{\textbf {u}}}\right) \cdot \partial _t^*{{\textbf {V}}}d\sigma \right| \\  &\quad \le C(T)\left( \left\| \nabla _\Gamma (v_{\textbf {n}})^2\right\| _{{\textbf {L}}^\infty (\Gamma (t))}+\left\| \partial ^\circ _t\widehat{{\textbf {u}}}\right\| _{{\textbf {L}}^\infty (\Gamma (t))}\right. \\  &\qquad \left. +(\left\| \widehat{{\textbf {u}}}\right\| _{{\textbf {L}}^\infty (\Gamma (t))}+\left\| \nabla _\Gamma \mu \right\| )\left\| \nabla _\Gamma \widehat{{\textbf {u}}}\right\| _{{\textbf {L}}^\infty (\Gamma (t))}+C\left\| v_{\textbf {n}}{\textbf {H}}\right\| _{{\textbf {L}}^\infty (\Gamma (t))}\left\| \widehat{{\textbf {u}}}\right\| _{{\textbf {L}}^\infty (\Gamma (t))}\right) \left\| \partial _t^*{{\textbf {V}}}\right\| \\  &\quad \le C(T)+\frac{\rho ^*}{16}\left\| \partial _t^*{{\textbf {V}}}\right\| ^2. \end{aligned}$$

In conclusion, we have

$$\begin{aligned}&\left| \int _{\Gamma (t)}\left( 2{\textbf {P}}\textrm{div}_\Gamma (\nu (\varphi ){\mathcal {E}}_S(\widehat{{\textbf {u}}}))+2{\textbf {P}}\textrm{div}_\Gamma (\nu (\varphi )v_{\textbf {n}}{\textbf {H}})\right) \cdot \partial _t^*{{\textbf {V}}}d\sigma \right| \\  &\quad \le C(\left\| \varphi \right\| _{H^1(\Gamma (t))}\left\| {\mathcal {E}}_S(\widehat{{\textbf {u}}})\right\| _{{\textbf {L}}^\infty (\Gamma (t))}+\left\| \widehat{{\textbf {u}}}\right\| _{{\textbf {H}}^2(\Gamma (t))})\left\| \partial _t^*{{\textbf {V}}}\right\| \\  &\qquad +C(\left\| \varphi \right\| _{H^1(\Gamma (t))}\left\| v_{\textbf {n}}{\textbf {H}}\right\| _{{\textbf {L}}^\infty (\Gamma (t))}+\left\| v_{\textbf {n}}{\textbf {H}}\right\| _{{\textbf {H}}^1(\Gamma (t))})\left\| \partial _t^*{{\textbf {V}}}\right\| \\  &\quad \le C(T)+\frac{\rho _*}{16}\left\| \partial _t^*{{\textbf {V}}}\right\| ^2. \end{aligned}$$

Therefore, to sum up, we have from (5.25), recalling that $\rho \ge \rho _*$,

$$\begin{aligned}&\frac{d}{dt}\int _{\Gamma (t)}\nu (\varphi ) \left| {\mathcal {E}}_S({{\textbf {V}}})\right| ^2d\sigma +\frac{9\rho _*}{16}\left\| \partial _t^*{{\textbf {V}}}\right\| ^2\\  &\quad \le C(T,\epsilon )(1+\left\| \partial ^\circ _t\varphi \right\| ^2+\left\| \mu \right\| _{H^3(\Gamma (t))}+\left\| {\mathcal {E}}_S({{\textbf {V}}})\right\| ^2)\left\| {\mathcal {E}}_S({{\textbf {V}}})\right\| ^2+3\epsilon \left\| {{\textbf {V}}}\right\| _{{\textbf {H}}^2(\Gamma (t))}^2. \end{aligned}$$

In conclusion, we sum up this inequality with (5.21) multiplied by $\tfrac{\rho _*}{16C(\omega ,T)}$, and we choose $\epsilon =\tfrac{\rho _*}{64C(\omega ,T)}$, to deduce in the end that

$$\begin{aligned}&\frac{d}{dt}\int _{\Gamma (t)}\nu (\varphi ) \left| {\mathcal {E}}_S({{\textbf {V}}})\right| ^2d\sigma +\frac{\rho _*}{2}\left\| \partial _t^*{{\textbf {V}}}\right\| ^2+\tfrac{\rho _*}{64C(\omega ,T)}\left\| {{\textbf {V}}}\right\| _{{\textbf {H}}^2(\Gamma (t))}^2\\  &\quad \le C(T)(1+\left\| \partial ^\circ _t\varphi \right\| ^2+\left\| \mu \right\| _{H^3(\Gamma (t))}+\left\| {\mathcal {E}}_S({{\textbf {V}}})\right\| ^2)\left\| {\mathcal {E}}_S({{\textbf {V}}})\right\| ^2,\quad \text {for almost any }t\in (0,T_m), \end{aligned}$$

which entails, thanks to (5.15)-(5.16) and (2.6), that, by the Gronwall lemma, since $T_m<T$,

5.26 $$\begin{aligned} \left\| {{\textbf {V}}}\right\| _{L^\infty _{{\textbf {H}}^1_\sigma (T_m)}}+\left\| {{\textbf {V}}}\right\| _{L^2_{{\textbf {H}}^2(T_m)}}+\left\| {{\textbf {V}}}\right\| _{H^1_{{\textbf {L}}^2(T_m)}}\le C(T). \end{aligned}$$

Now, let us observe that the regularity (5.16), together with (5.26), entails (see also Lemma 2.1) that

$$ \left\| {{\textbf {V}}}\right\| _{C^0_{{\textbf {H}}^1_\sigma (T_m)}}+\left\| \varphi \right\| _{C^0_{H^2 (T_m)}}+\left\| \mu \right\| _{C^0_{H^1(T_m)}}\le C(T). $$

This means that we can also extend the solution at $t=T_m$, and, in particular, it holds

$$\begin{aligned} {{\textbf {V}}}(T_m)\in {\textbf {H}}^1_\sigma (\Gamma (T_m)),\quad \varphi (T_m)\in H^2(\Gamma (T_m)),\quad \mu (T_m)\in H^1(\Gamma (T_m)), \end{aligned}$$

together with $\left\| \varphi (T_m)\right\| _{L^\infty (\Gamma (T_m))}\le 1-2\delta _m$ (see (5.17)), for some $\delta _m\in (0,1)$. Moreover, it holds

$$ -\Delta _\Gamma \varphi (T_m)+\Psi '(\varphi (T_m))=\mu (T_m),\quad \text {for almost any }x\in \Gamma (T_m), $$

entailing by elliptic regularity, exploiting the separation property, that

$$ \varphi (T_m)\in H^3(\Gamma (T_m))\hookrightarrow B_{p,q}^{4-\frac{4}{q}}(\Gamma (T_m)), $$

for some $$q\in (2,4]$$ and $$p>4$$, see also (5.1). This means that the pair $$({\textbf {v}}(T_m),\varphi (T_m))$$, with $${\textbf {v}}(T_m):={{\textbf {V}}}(T_m)+\widehat{{\textbf {u}}}(T_m)$$, is sufficiently regular to be considered as initial datum in the application of [4, Theorem 4.1] (together with [4, Remarks 4.3$$-$$4.4]) to deduce, following word by word the argument in Step 0, that there exists a strong solution according to Definition 3.1, defined on an interval $$[T_m,T_1]$$, with $$T_1>T_m$$, extending the one on $$[0,T_m)$$. This contradicts the maximality of the considered solution on $$(0,T_m)$$, entailing that $$T_m=T$$, i.e., any strong solution is globally defined on [0, *T*], concluding the first part of the proof. We now need to prove the uniqueness of global strong solutions.

*Step 3. Continuous dependence and Uniqueness of strong solutions.* Let us consider two sets of initial data $({{\textbf {V}}}_{0,i},\varphi _{0,i})$, $i=1,2$, satisfying the assumptions of Theorem 3.3 and $\overline{\varphi }_1=\overline{\varphi }_2$, so that there exist two global strong solutions $({{\textbf {V}}}_i,\varphi _i,p_i)$ departing from these data. We also define $\rho _i:=\rho (\varphi _i)$. Let us set ${{\textbf {V}}}:={{\textbf {V}}}_1-{{\textbf {V}}}_2$, $\varphi :=\varphi _1-\varphi _2$, $\pi =\pi _1-\pi _2$ and consider the equations satisfied by these variables. In particular, we multiply the equation for the difference of velocities by ${{\textbf {V}}}$ and integrate over $\Gamma (t)$, whereas we take the gradient of the Cahn-Hilliard equation (written for the difference $\varphi$), multiply it by $\nabla _\Gamma \varphi$ and again integrate over $\Gamma (t)$. We have

5.27 $$\begin{aligned} {\left\{ \begin{array}{ll} \int _{\Gamma (t)}\rho _1\partial ^\circ _t{{\textbf {V}}}\cdot {{\textbf {V}}}d\sigma +\int _{\Gamma (t)}(\rho _1-\rho _2)\partial ^\circ _t{{\textbf {V}}}_2\cdot {{\textbf {V}}}d\sigma +\int _{\Gamma (t)}\rho _1({{\textbf {V}}}\cdot \nabla _\Gamma ){{\textbf {V}}}_1\cdot {{\textbf {V}}}d\sigma \\ \qquad +\int _{\Gamma (t)}(\rho _1-\rho _2)({{\textbf {V}}}_2\cdot \nabla _\Gamma ){{\textbf {V}}}_1\cdot {{\textbf {V}}}d\sigma \int _{\Gamma (t)}\rho _2({{\textbf {V}}}_2\cdot \nabla _\Gamma ){{\textbf {V}}}\cdot {{\textbf {V}}}d\sigma \\ \qquad +\int _{\Gamma (t)}({\textbf {J}}_{\rho _1}\cdot \nabla _\Gamma ){{\textbf {V}}}\cdot {{\textbf {V}}}d\sigma +\int _{\Gamma (t)}(({\textbf {J}}_{\rho _1}-{\textbf {J}}_{\rho _2})\cdot \nabla _\Gamma {{\textbf {V}}}_2)\cdot {{\textbf {V}}}d\sigma \\ \qquad +\int _{\Gamma (t)}\rho _1(\widehat{{\textbf {u}}}\cdot \nabla _\Gamma ){{\textbf {V}}}\cdot {{\textbf {V}}}d\sigma +\int _{\Gamma (t)}(\rho _1-\rho _2)(\widehat{{\textbf {u}}}\cdot \nabla _\Gamma ){{\textbf {V}}}_2\cdot {{\textbf {V}}}d\sigma \\ \qquad +\int _{\Gamma (t)}\rho _1({{\textbf {V}}}\cdot \nabla _\Gamma )\widehat{{\textbf {u}}}\cdot {{\textbf {V}}}d\sigma +\int _{\Gamma (t)}(\rho _1-\rho _2)({{\textbf {V}}}_2\cdot \nabla _\Gamma )\widehat{{\textbf {u}}}\cdot {{\textbf {V}}}d\sigma +\int _{\Gamma (t)}\rho _1v_{\textbf {n}}{\textbf {H}}{{\textbf {V}}}\cdot {{\textbf {V}}}d\sigma \\ \qquad +\int _{\Gamma (t)}(\rho _1-\rho _2)v_{\textbf {n}}{\textbf {H}}{{\textbf {V}}}_2\cdot {{\textbf {V}}}d\sigma +\int _{\Gamma (t)}v_{\textbf {n}}{\textbf {H}}({\textbf {J}}_{\rho _1}- {\textbf {J}}_{\rho _2})\cdot {{\textbf {V}}}d\sigma \\ \qquad +2\int _{\Gamma (t)}\nu (\varphi _1)\left| {\mathcal {E}}_S({{\textbf {V}}})\right| ^2d\sigma +2\int _{\Gamma (t)}(\nu (\varphi _1)-\nu (\varphi _2)){\mathcal {E}}_S({{\textbf {V}}}_2):{\mathcal {E}}_S({{\textbf {V}}})d\sigma \\ \quad =\int _{\Gamma (t)}(\nabla _\Gamma \varphi _1\otimes \nabla _\Gamma \varphi ):\nabla _\Gamma {{\textbf {V}}}d\sigma +\int _{\Gamma (t)}(\nabla _\Gamma \varphi \otimes \nabla _\Gamma \varphi _2):\nabla _\Gamma {{\textbf {V}}}d\sigma \\ \qquad -2\int _{\Gamma (t)}(\nu (\varphi _1)-\nu (\varphi _2))v_{\textbf {n}}{\textbf {H}}:\nabla _\Gamma {{\textbf {V}}}d\sigma -\int _{\Gamma (t)}(\rho _1-\rho _2)\partial ^\circ _t\widehat{{\textbf {u}}}\cdot {{\textbf {V}}}d\sigma \\ \qquad -\int _{\Gamma (t)}(\rho _1-\rho _2)(\widehat{{\textbf {u}}}\cdot \nabla _\Gamma )\widehat{{\textbf {u}}}\cdot {{\textbf {V}}}d\sigma \\ \qquad -\int _{\Gamma (t)}(({\textbf {J}}_{\rho _1}- {\textbf {J}}_{\rho _2})\cdot \nabla _\Gamma )\widehat{{\textbf {u}}}\cdot {{\textbf {V}}}d\sigma -\int _{\Gamma (t)}(\rho _1-\rho _2)(v_{\textbf {n}}{\textbf {H}}\widehat{{\textbf {u}}}-\frac{1}{2}\nabla _\Gamma (v_{\textbf {n}})^2)\cdot {{\textbf {V}}}d\sigma \\ \qquad -2\int _{\Gamma (t)}(\nu (\varphi _1)-\nu (\varphi _2)){\mathcal {E}}_S(\widehat{{\textbf {u}}}):{\mathcal {E}}_S({{\textbf {V}}})d\sigma , \\ \\ \int _{\Gamma (t)}\nabla _\Gamma \partial ^\circ _t\varphi \cdot \nabla _\Gamma \varphi d\sigma -\int _{\Gamma (t)}\nabla _\Gamma \Delta (\mu _1-\mu _2)\cdot \nabla _\Gamma \varphi d\sigma \\ \quad +\int _{\Gamma (t)}\nabla _\Gamma (\nabla _\Gamma (\varphi _1)\cdot {{\textbf {V}}})\cdot \nabla _\Gamma \varphi d\sigma +\int _{\Gamma (t)}\nabla _\Gamma (\nabla _\Gamma \varphi \cdot ({{\textbf {V}}}_2+\widehat{{\textbf {u}}}))\cdot \nabla _\Gamma \varphi d\sigma =0. \end{array}\right. } \end{aligned}$$

First, we recall that, by the regularity of strong solutions, there exists $\delta >0$ such that, for $i=1,2$,

$$ \sup _{t\in [0,T]}\left\| \varphi _i\right\| _{L^\infty (\Gamma (t))}\le 1-\delta , $$

so that it holds, for $i=1,2$,

5.28 $$\begin{aligned}&\left\| {{\textbf {V}}}_i\right\| _{H^1_{{\textbf {L}}^2}}+\left\| {{\textbf {V}}}_i\right\| _{L^2_{{\textbf {H}}^2}}+\left\| {{\textbf {V}}}_i\right\| _{L^\infty _{{\textbf {H}}^1_\sigma }}+\left\| \varphi _i\right\| _{L^\infty _{H^3}}+\left\| \varphi _i\right\| _{H^1_{H^1}}\nonumber \\&\quad +\left\| F''(\varphi _i)\right\| _{L^\infty (\Gamma (t))}+\left\| F'''(\varphi _i)\right\| _{L^\infty (\Gamma (t))}+\left\| F^{(iv)}(\varphi _i)\right\| _{L^\infty (\Gamma (t))}\le C(T), \end{aligned}$$

for almost any $t\in [0,T]$. Moreover, concerning the regularity of the flow map, (5.11) holds. We start form the second identity in (5.27). By (2.12) we have

$$\begin{aligned} \frac{1}{2}\frac{d}{dt}\left\| \nabla _\Gamma \varphi \right\| ^2&=\int _{\Gamma (t)}\nabla _\Gamma \varphi \cdot \nabla _\Gamma \partial ^\circ _t\varphi +\frac{1}{2}\int _{\Gamma (t)}\left| \nabla _\Gamma \varphi \right| ^2v_{\textbf {n}}Hd\sigma \\  &\quad -\int _{\Gamma (t)}\nabla _\Gamma \varphi \cdot {\mathcal {E}}_S({{\textbf {V}}}_{\textbf {n}})\nabla _\Gamma \varphi d\sigma , \end{aligned}$$

as well as, integrating by parts and recalling that $\Psi ''=F''-\theta _0$,

$$\begin{aligned}&-\int _{\Gamma (t)}\nabla _\Gamma \Delta (\mu _1-\mu _2)\cdot \nabla _\Gamma \varphi d\sigma =\int _{\Gamma (t)}\left| \nabla _\Gamma \Delta _\Gamma \varphi \right| ^2d\sigma \\&\qquad +\int _{\Gamma (t)}(F'''(\varphi _1)-F'''(\varphi _2))\left| \nabla _\Gamma \varphi _1\right| ^2\Delta _{\Gamma }\varphi d\sigma \\  &\qquad +\int _{\Gamma (t)}F'''(\varphi _2){\nabla _\Gamma \varphi }\cdot \nabla _\Gamma (\varphi _1+\varphi _2)\Delta _\Gamma \varphi d\sigma +\int _{\Gamma (t)}F''(\varphi _1)\left| \Delta _\Gamma \varphi \right| ^2d\sigma \\  &\qquad +\int _{\Gamma (t)}(F''(\varphi _1)-F''(\varphi _2))\Delta _\Gamma \varphi _2\Delta _\Gamma \varphi d\sigma -\int _{\Gamma (t)}\theta _0\left| \Delta _\Gamma \varphi \right| ^2d\sigma . \end{aligned}$$

Furthermore, we have

5.29 $$\begin{aligned} \int _{\Gamma (t)}\nabla _\Gamma (\nabla _\Gamma \varphi _1\cdot {{\textbf {V}}})\cdot \nabla _\Gamma \varphi d\sigma =\int _{\Gamma (t)}\nabla _\Gamma (\nabla _\Gamma \varphi _1){{\textbf {V}}}\cdot \nabla _\Gamma \varphi d\sigma +\int _{\Gamma (t)}\nabla _\Gamma ^T{{\textbf {V}}}\nabla _\Gamma \varphi _1\cdot \nabla _\Gamma \varphi d\sigma , \end{aligned}$$

and analogously,

5.30 $$\begin{aligned}&\int _{\Gamma (t)}\nabla _\Gamma (\nabla _\Gamma \varphi \cdot ({{\textbf {V}}}_2+\widehat{{\textbf {u}}}))\cdot \nabla _\Gamma \varphi d\sigma \nonumber \\&\quad =\int _{\Gamma (t)}\nabla _\Gamma (\nabla _\Gamma \varphi )({{\textbf {V}}}_2+\widehat{{\textbf {u}}})\cdot \nabla _\Gamma \varphi d\sigma +\int _{\Gamma (t)}\nabla _\Gamma ^T({{\textbf {V}}}_2+\widehat{{\textbf {u}}})\nabla _\Gamma \varphi \cdot \nabla _\Gamma \varphi d\sigma . \end{aligned}$$

Therefore, from (5.27) we deduce, recalling $F''\ge \theta$,

5.31 $$\begin{aligned}&\frac{1}{2}\frac{d}{dt}\left\| \nabla _\Gamma \varphi \right\| ^2+\left\| \nabla _\Gamma \Delta _\Gamma \varphi \right\| ^2+\theta \left\| \Delta _\Gamma \varphi \right\| ^2\nonumber \\&\quad \le \underbrace{\frac{1}{2}\int _{\Gamma (t)}\left| \nabla _\Gamma \varphi \right| ^2v_{\textbf {n}}Hd\sigma -\int _{\Gamma (t)}\nabla _\Gamma \varphi :{\mathcal {E}}_S({{\textbf {V}}}_{\textbf {n}})\nabla _\Gamma \varphi d\sigma }_{I_1}\nonumber \\&\qquad \underbrace{-\int _{\Gamma (t)}(F'''(\varphi _1)-F'''(\varphi _2))\left| \nabla _\Gamma \varphi _1\right| ^2\Delta _{\Gamma }\varphi d\sigma }_{I_2}\nonumber \\&\qquad \underbrace{-\int _{\Gamma (t)}F'''(\varphi _2){\nabla _\Gamma \varphi }\cdot \nabla _\Gamma (\varphi _1+\varphi _2)\Delta _\Gamma \varphi d\sigma }_{I_3}\nonumber \\&\qquad \underbrace{-\int _{\Gamma (t)}(F''(\varphi _1)-F''(\varphi _2))\Delta _\Gamma \varphi _2\Delta _\Gamma \varphi d\sigma +\int _{\Gamma (t)}\theta _0\left| \Delta _\Gamma \varphi \right| ^2d\sigma }_{I_4}\nonumber \\  &\qquad \underbrace{-\int _{\Gamma (t)}\nabla _\Gamma (\nabla _\Gamma \varphi _1){{\textbf {V}}}\cdot \nabla _\Gamma \varphi -\int _{\Gamma (t)}\nabla _\Gamma ^T{{\textbf {V}}}\nabla _\Gamma \varphi _1\cdot \nabla _\Gamma \varphi d\sigma }_{I_5}\nonumber \\  &\qquad \underbrace{-\int _{\Gamma (t)}\nabla _\Gamma (\nabla _\Gamma \varphi )({{\textbf {V}}}_2+\widehat{{\textbf {u}}})\cdot \nabla _\Gamma \varphi -\int _{\Gamma (t)}\nabla _\Gamma ^T({{\textbf {V}}}_2+\widehat{{\textbf {u}}})\nabla _\Gamma \varphi \cdot \nabla _\Gamma \varphi d\sigma }_{I_6}. \end{aligned}$$

We now estimate all these terms separately. First, we have, exploiting (5.11),

$$\begin{aligned} \left| I_1\right| \le C(T)\left\| \nabla _\Gamma \varphi \right\| ^2. \end{aligned}$$

Then, exploiting (5.28) and $H^3(\Gamma (t))\hookrightarrow W^{1,\infty }(\Gamma (t))$, we have, thanks to the strict separation property,

$$\begin{aligned} \left| I_2\right|&= \left| \int _{\Gamma (t)}\int _0^1 F^{(iv)}(s\varphi _1+(1-s)\varphi _2)\varphi ds\left| \nabla _\Gamma \varphi _1\right| ^2\Delta _\Gamma \varphi d\sigma \right| \\  &\le C\left\| \varphi _1\right\| ^2_{W^{1,\infty }(\Gamma (t))}\left\| \varphi \right\| \left\| \Delta _\Gamma \varphi \right\| \le C(T)\left\| \nabla _\Gamma \varphi \right\| ^2+\frac{\theta }{8} \left\| \Delta _\Gamma \varphi \right\| ^2, \end{aligned}$$

where we used Poincaré’s inequality, since $\overline{\varphi }\equiv 0$. Then, similarly,

$$\begin{aligned} \left| I_3\right|&\le C\left\| F'''(\varphi _2)\right\| _{L^\infty (\Gamma (t))}\left\| \nabla _\Gamma \varphi \right\| (\left\| \nabla _\Gamma \varphi _1\right\| _{{\textbf {L}}^\infty (\Gamma (t))}+\left\| \nabla _\Gamma \varphi _2\right\| _{{\textbf {L}}^\infty (\Gamma (t))})\left\| \Delta _\Gamma \varphi \right\| \\  &\le C(T)\left\| \nabla _\Gamma \varphi \right\| ^2+\frac{\theta }{8} \left\| \Delta \varphi \right\| ^2. \end{aligned}$$

Proceeding in the estimate, we have, in a similar way, by the embedding $H^3(\Gamma (t))\hookrightarrow W^{2,4}(\Gamma (t))$,

$$\begin{aligned} \left| I_4\right|&=\left| \int _{\Gamma (t)}\int _0^1 F'''(s\varphi _1+(1-s)\varphi _2)\varphi ds\Delta _\Gamma \varphi _2\Delta _\Gamma \varphi d\sigma \right| +\theta _0\left\| \Delta _\Gamma \varphi \right\| ^2\\  &\le C(T)\left\| \varphi \right\| _{L^4(\Gamma (t))}\left\| \Delta _\Gamma \varphi _2\right\| _{L^4(\Gamma (t))}\left\| \Delta _\Gamma \varphi \right\| +C\left\| \nabla _\Gamma \Delta _\Gamma \varphi \right\| \left\| \nabla _\Gamma \varphi \right\| \\  &\le C(T)\left\| \nabla _\Gamma \varphi \right\| ^2+\frac{\theta }{8}\left\| \Delta _\Gamma \varphi \right\| ^2+\frac{1}{2}\left\| \nabla _\Gamma \Delta _\Gamma \varphi \right\| ^2, \end{aligned}$$

where we used that

$$\begin{aligned} \int _{\Gamma (t)}\left| \Delta _\Gamma \varphi \right| ^2d\sigma =-\int _{\Gamma (t)}\nabla \Delta _\Gamma \varphi \cdot \nabla _\Gamma \varphi d\sigma \le \left\| \nabla \Delta _\Gamma \varphi \right\| \left\| \nabla _\Gamma \varphi \right\| . \end{aligned}$$

About the advective terms, we have, by standard inequalities, (2.7) and (2.8), recalling (5.11) and (5.28),

$$\begin{aligned} \left| I_5\right|&\le C\left\| \varphi _1\right\| _{W^{2,4}(\Gamma (t))}\left\| {{\textbf {V}}}\right\| _{{\textbf {L}}^4(\Gamma (t))}\left\| \nabla _\Gamma \varphi \right\| \\&\quad +C(T)(1+\left\| {{\textbf {V}}}\right\| _{{\textbf {H}}^1_\sigma (\Gamma (t))})\left\| \nabla _\Gamma \varphi _1\right\| _{{\textbf {L}}^4(\Gamma (t))}\left\| \nabla _\Gamma \varphi \right\| _{{\textbf {L}}^4(\Gamma (t))}\\  &\le C(T)(\left\| {{\textbf {V}}}\right\| +\left\| {{\textbf {V}}}\right\| ^\frac{1}{2}\left\| {\mathcal {E}}_S({{\textbf {V}}})\right\| ^\frac{1}{2})\left\| \nabla _\Gamma \varphi \right\| \\  &\quad +C(T)(1+\left\| {{\textbf {V}}}\right\| +\left\| {\mathcal {E}}_S({{\textbf {V}}})\right\| )(\left\| \nabla _\Gamma \varphi \right\| +\left\| \nabla _\Gamma \varphi \right\| ^\frac{1}{2}\left\| \Delta _\Gamma \varphi \right\| ^\frac{1}{2})\\  &\le C(T)(\left\| {{\textbf {V}}}\right\| ^2+\left\| \nabla _\Gamma \varphi \right\| ^2)+\frac{3}{8}\nu _*\left\| {\mathcal {E}}_S({{\textbf {V}}})\right\| ^2+\frac{\theta }{8}\left\| \Delta _\Gamma \varphi \right\| ^2. \end{aligned}$$

Here we have also used the inequality

5.32 $$\begin{aligned} \left\| \varphi \right\| _{W^{1,4}(\Gamma (t))}&\le C(T)\left\| \varphi \right\| _{H^1(\Gamma (t))}^\frac{1}{2}\left\| \Delta _\Gamma \varphi \right\| ^\frac{1}{2}\nonumber \\  &\le C(T)\left\| \nabla _\Gamma \varphi \right\| ^\frac{1}{2}(\left\| \varphi \right\| ^\frac{1}{2}+\left\| \Delta _\Gamma \varphi \right\| ^\frac{1}{2})\nonumber \\&\le C(T)(\left\| \nabla _\Gamma \varphi \right\| +\left\| \nabla _\Gamma \varphi \right\| ^\frac{1}{2}\left\| \Delta _\Gamma \varphi \right\| ^\frac{1}{2}), \end{aligned}$$

which holds by Poincaré’s inequality. In conclusion, by similar estimates, recalling again (2.9), (5.11), (5.28), and (5.32),

$$\begin{aligned} \left| I_6\right|&\le C\left\| \varphi \right\| _{H^2(\Gamma (t))}(\left\| {{\textbf {V}}}_2\right\| _{{\textbf {L}}^\infty (\Gamma (t))}+1)\left\| \nabla _\Gamma \varphi \right\| +C(T)(1+\left\| {{\textbf {V}}}_2\right\| _{{\textbf {H}}^1_\sigma (\Gamma (t))})\left\| \nabla _\Gamma \varphi _1\right\| _{{\textbf {L}}^4(\Gamma (t))}^2\\  &\le C(\left\| \varphi \right\| +\left\| \Delta _\Gamma \varphi \right\| )(1+\left\| {{\textbf {V}}}_2\right\| ^\frac{1}{2}\left\| {{\textbf {V}}}_2\right\| _{{\textbf {H}}^2(\Gamma (t))}^\frac{1}{2})\left\| \nabla _\Gamma \varphi \right\| +C(T)(\left\| \nabla _\Gamma \varphi \right\| ^2+\left\| \nabla _\Gamma \varphi \right\| \left\| \Delta _\Gamma \varphi \right\| )\\  &\le C(T)(1+\left\| {{\textbf {V}}}_2\right\| _{{\textbf {H}}^2(\Gamma (t))}^\frac{1}{2}+\left\| {{\textbf {V}}}_2\right\| _{{\textbf {H}}^2(\Gamma (t))})\left\| \nabla _\Gamma \varphi \right\| ^2+\frac{\theta }{8}\left\| \Delta _\Gamma \varphi \right\| ^2. \end{aligned}$$

Therefore, we have obtained from (5.31) that

5.33 $$\begin{aligned}&\frac{1}{2}\frac{d}{dt}\left\| \nabla _\Gamma \varphi \right\| ^2+\frac{1}{2}\left\| \nabla _\Gamma \Delta _\Gamma \varphi \right\| ^2+\frac{3\theta }{8}\left\| \Delta _\Gamma \varphi \right\| ^2\nonumber \\  &\quad \le C(T)(1+\left\| {{\textbf {V}}}_2\right\| _{{\textbf {H}}^2(\Gamma (t))}^\frac{1}{2}+\left\| {{\textbf {V}}}_2\right\| _{{\textbf {H}}^2(\Gamma (t))})(\left\| \nabla _\Gamma \varphi \right\| ^2+\left\| {{\textbf {V}}}\right\| ^2)+\frac{3}{8}\nu _*\left\| {\mathcal {E}}_S({{\textbf {V}}})\right\| ^2. \end{aligned}$$

Now we need to analyze (5.27)$_1$. First, we notice that

$$\begin{aligned} \frac{1}{2}\frac{d}{dt}\int _{\Gamma (t)}\rho _1\left| {{\textbf {V}}}\right| ^2d\sigma&=\int _{\Gamma (t)}\rho _1\partial ^\circ _t{{\textbf {V}}}\cdot {{\textbf {V}}}d\sigma +\frac{1}{2}\int _{\Gamma (t)}\rho '(\varphi _1)\partial ^\circ _t\varphi _1\left| {{\textbf {V}}}\right| ^2d\sigma \\&\quad +\int _{\Gamma (t)}\rho _1\left| {{\textbf {V}}}\right| ^2v_{\textbf {n}}Hd\sigma , \end{aligned}$$

so that, recalling also $\nu \ge \nu _*>0$, we can rewrite (5.27)$_1$ as

5.34 $$\begin{aligned}&\frac{1}{2}\frac{d}{dt}\int _{\Gamma (t)}\rho _1\left| {{\textbf {V}}}\right| ^2d\sigma +2\nu _*\int _{\Gamma (t)}\left| {\mathcal {E}}_S({{\textbf {V}}})\right| ^2d\sigma \nonumber \\&\quad \le \underbrace{\frac{1}{2}\int _{\Gamma (t)}\rho '(\varphi _1)\partial ^\circ _t\varphi _1\left| {{\textbf {V}}}\right| ^2d\sigma }_{I_7}+\underbrace{\int _{\Gamma (t)}\rho _1\left| {{\textbf {V}}}\right| ^2v_{\textbf {n}}Hd\sigma }_{I_8}\nonumber \\  &\qquad -\underbrace{\int _{\Gamma (t)}(\rho _1-\rho _2)\partial ^\circ _t{{\textbf {V}}}_2\cdot {{\textbf {V}}}d\sigma }_{I_9}-\underbrace{\int _{\Gamma (t)}\rho _1({{\textbf {V}}}\cdot \nabla _\Gamma ){{\textbf {V}}}_1\cdot {{\textbf {V}}}d\sigma -\int _{\Gamma (t)}(\rho _1-\rho _2)({{\textbf {V}}}_2\cdot \nabla _\Gamma ){{\textbf {V}}}_1\cdot {{\textbf {V}}}d\sigma }_{I_{10}}\nonumber \\&\qquad -\underbrace{\int _{\Gamma (t)}\rho _2({{\textbf {V}}}_2\cdot \nabla _\Gamma ){{\textbf {V}}}\cdot {{\textbf {V}}}d\sigma }_{I_{11}}-\underbrace{\int _{\Gamma (t)}({\textbf {J}}_{\rho _1}\cdot \nabla _\Gamma ){{\textbf {V}}}\cdot {{\textbf {V}}}d\sigma -\int _{\Gamma (t)}(({\textbf {J}}_{\rho _1}-{\textbf {J}}_{\rho _2})\cdot \nabla _\Gamma {{\textbf {V}}}_2)\cdot {{\textbf {V}}}d\sigma }_{I_{12}}\nonumber \\  &\qquad -\underbrace{\int _{\Gamma (t)}\rho _1(\widehat{{\textbf {u}}}\cdot \nabla _\Gamma ){{\textbf {V}}}\cdot {{\textbf {V}}}d\sigma -\int _{\Gamma (t)}(\rho _1-\rho _2)(\widehat{{\textbf {u}}}\cdot \nabla _\Gamma ){{\textbf {V}}}_2\cdot {{\textbf {V}}}d\sigma -\int _{\Gamma (t)}\rho _1({{\textbf {V}}}\cdot \nabla _\Gamma )\widehat{{\textbf {u}}}\cdot {{\textbf {V}}}d\sigma }_{I_{13}}\nonumber \\  &\qquad -\underbrace{\int _{\Gamma (t)}(\rho _1-\rho _2)({{\textbf {V}}}_2\cdot \nabla _\Gamma )\widehat{{\textbf {u}}}\cdot {{\textbf {V}}}d\sigma -\int _{\Gamma (t)}\rho _1v_{\textbf {n}}{\textbf {H}}{{\textbf {V}}}\cdot {{\textbf {V}}}d\sigma -\int _{\Gamma (t)}(\rho _1-\rho _2)(v_{\textbf {n}}{\textbf {H}}{{\textbf {V}}}_2-\frac{1}{2}\nabla _\Gamma (v_{\textbf {n}})^2)\cdot {{\textbf {V}}}d\sigma }_{I_{14}}\nonumber \\  &\qquad -\underbrace{\int _{\Gamma (t)}v_{\textbf {n}}{\textbf {H}}({\textbf {J}}_{\rho _1}-{\textbf {J}}_{\rho _2})\cdot {{\textbf {V}}}d\sigma }_{I_{15}}-\underbrace{2\int _{\Gamma (t)}(\nu (\varphi _1)-\nu (\varphi _2)){\mathcal {E}}_S({{\textbf {V}}}_2):{\mathcal {E}}_S({{\textbf {V}}})d\sigma }_{I_{16}}\nonumber \\  &\qquad \underbrace{+\int _{\Gamma (t)}(\nabla _\Gamma \varphi _1\otimes \nabla _\Gamma \varphi ):\nabla _\Gamma {{\textbf {V}}}d\sigma +\int _{\Gamma (t)}(\nabla _\Gamma \varphi \otimes \nabla _\Gamma \varphi _2):\nabla _\Gamma {{\textbf {V}}}d\sigma }_{I_{17}}\nonumber \\  &\qquad -\underbrace{2\int _{\Gamma (t)}(\nu (\varphi _1)-\nu (\varphi _2))v_{\textbf {n}}{\textbf {H}}:\nabla _\Gamma {{\textbf {V}}}d\sigma }_{I_{18}}\nonumber \\  &\qquad -\underbrace{\int _{\Gamma (t)}(\rho _1-\rho _2)\partial ^\circ _t\widehat{{\textbf {u}}}\cdot {{\textbf {V}}}-\int _{\Gamma (t)}(\rho _1-\rho _2)(\widehat{{\textbf {u}}}\cdot \nabla _\Gamma )\widehat{{\textbf {u}}}\cdot {{\textbf {V}}}d\sigma }_{I_{19}}-\underbrace{\int _{\Gamma (t)}(({\textbf {J}}_{\rho _1}-{\textbf {J}}_{\rho _2})\cdot \nabla _\Gamma )\widehat{{\textbf {u}}}\cdot {{\textbf {V}}}d\sigma }_{I_{20}}\nonumber \\  &\qquad -\underbrace{\int _{\Gamma (t)}(\rho _1-\rho _2)v_{\textbf {n}}{\textbf {H}}\widehat{{\textbf {u}}}\cdot {{\textbf {V}}}d\sigma -2\int _{\Gamma (t)}(\nu (\varphi _1)-\nu (\varphi _2)){\mathcal {E}}_S(\widehat{{\textbf {u}}}):{\mathcal {E}}_S({{\textbf {V}}})d\sigma }_{I_{21}}. \end{aligned}$$

We need to estimate all these terms. Now, by (2.7) and (2.8),

$$\begin{aligned} \left| I_7\right|&\le C\left\| \partial ^\circ _t\varphi _1\right\| \left\| {{\textbf {V}}}\right\| _{{\textbf {L}}^4(\Gamma (t))}^2\le C\left\| \partial ^\circ _t\varphi _1\right\| \left\| {{\textbf {V}}}\right\| (\left\| {{\textbf {V}}}\right\| +\left\| {\mathcal {E}}_S({{\textbf {V}}})\right\| ) \\  &\le C\left\| \partial ^\circ _t\varphi _1\right\| ^2\left\| {{\textbf {V}}}\right\| ^2+\frac{\nu _*}{16}\left\| {\mathcal {E}}_S({{\textbf {V}}})\right\| ^2. \end{aligned}$$

Then, by (5.11),

$$\begin{aligned} \left| I_8\right| \le C(T)\left\| {{\textbf {V}}}\right\| ^2. \end{aligned}$$

Then, recalling that $\rho$ is linear, and again by (2.7),

$$\begin{aligned} \left| I_9\right|&\le C\left\| \varphi \right\| _{L^4(\Gamma (t))}\left\| \partial ^\circ _t{{\textbf {V}}}_2\right\| \left\| {{\textbf {V}}}\right\| _{{\textbf {L}}^4(\Gamma (t))}\\  &\le C\left\| \nabla _\Gamma \varphi \right\| \left\| \partial ^\circ _t{{\textbf {V}}}_2\right\| \left\| {{\textbf {V}}}\right\| ^\frac{1}{2}(\left\| {{\textbf {V}}}\right\| ^\frac{1}{2}+\left\| {\mathcal {E}}_S({{\textbf {V}}})\right\| ^\frac{1}{2})\\  &\le C(1+\left\| \partial ^\circ _t{{\textbf {V}}}_2\right\| ^2)(\left\| \nabla _\Gamma \varphi \right\| ^2+\left\| {{\textbf {V}}}\right\| ^2)+\frac{\nu _*}{16}\left\| {\mathcal {E}}_S({{\textbf {V}}})\right\| ^2. \end{aligned}$$

Then, recalling (5.28) and Agmon’s inequality (2.9),

$$\begin{aligned} \left| I_{10}\right|&\le C\left\| {{\textbf {V}}}\right\| _{{\textbf {L}}^4(\Gamma (t))}\left\| \nabla _\Gamma {{\textbf {V}}}_1\right\| \left\| {{\textbf {V}}}\right\| _{{\textbf {L}}^4(\Gamma (t))}+C\left\| \varphi \right\| _{L^4(\Gamma (t))}\left\| {{\textbf {V}}}_2\right\| _{{\textbf {L}}^\infty (\Gamma (t))}\left\| \nabla _\Gamma {{\textbf {V}}}_1\right\| \left\| {{\textbf {V}}}\right\| _{{\textbf {L}}^4(\Gamma (t))}\\  &\le C(T)\left\| {{\textbf {V}}}\right\| (\left\| {{\textbf {V}}}\right\| +\left\| {\mathcal {E}}_S({{\textbf {V}}})\right\| )+C(T)\left\| \nabla _\Gamma \varphi \right\| \left\| {{\textbf {V}}}_2\right\| ^\frac{1}{2}\left\| {{\textbf {V}}}_2\right\| _{{\textbf {H}}^2(\Gamma (t))}^\frac{1}{2}\left\| {{\textbf {V}}}\right\| ^\frac{1}{2}(\left\| {{\textbf {V}}}\right\| ^\frac{1}{2}+\left\| {\mathcal {E}}_S({{\textbf {V}}})\right\| ^\frac{1}{2})\\  &\le C(T)(1+\left\| {{\textbf {V}}}_2\right\| _{{\textbf {H}}^2(\Gamma (t))})(\left\| {{\textbf {V}}}\right\| ^2+\left\| \nabla _\Gamma \varphi \right\| ^2)+\frac{\nu _*}{16}\left\| {\mathcal {E}}_S({{\textbf {V}}})\right\| ^2. \end{aligned}$$

Proceeding in the estimates, we have, similarly,

$$\begin{aligned} \left| I_{11}\right|&\le C\left\| {{\textbf {V}}}_2\right\| _{{\textbf {L}}^\infty (\Gamma (t))}\left\| \nabla _\Gamma {{\textbf {V}}}\right\| \left\| {{\textbf {V}}}\right\| \le C\left\| {{\textbf {V}}}_2\right\| ^\frac{1}{2}\left\| {{\textbf {V}}}_2\right\| _{{\textbf {H}}^2(\Gamma (t))}^\frac{1}{2}(\left\| {{\textbf {V}}}\right\| +\left\| {\mathcal {E}}_S({{\textbf {V}}})\right\| )\left\| {{\textbf {V}}}\right\| \\  &\le C(\left\| {{\textbf {V}}}_2\right\| _{{\textbf {H}}^2(\Gamma (t))}^\frac{1}{2}+\left\| {{\textbf {V}}}_2\right\| _{{\textbf {H}}^2(\Gamma (t))})\left\| {{\textbf {V}}}\right\| ^2+\frac{\nu _*}{16}\left\| {\mathcal {E}}_S({{\textbf {V}}})\right\| ^2. \end{aligned}$$

Now, recalling the definition of ${\textbf {J}}_{\rho _i}$, we have

5.35 $$\begin{aligned} {\textbf {J}}_{\rho _1}-{\textbf {J}}_{\rho _2}&=-\frac{\rho _1-\rho _2}{2}\nabla _\Gamma (\mu _1-\mu _2)\nonumber \\  &=\frac{\rho _1-\rho _2}{2}\nabla _\Gamma \Delta _\Gamma \varphi -\frac{\rho _1-\rho _2}{2}(F''(\varphi _1)-\theta _0)\nabla _\Gamma \varphi -\frac{\rho _1-\rho _2}{2}(F''(\varphi _1)\nonumber \\  &\quad -F''(\varphi _2))\nabla _\Gamma \varphi _2, \end{aligned}$$

so that, since $F''(\varphi _1)-F''(\varphi _2)=\int _0^1 F'''(s\varphi _1+(1-s)\varphi _2)\varphi ds$, recalling (2.7), (2.8), Agmon’s inequality (2.9), (5.28) and the strict separation property,

$$\begin{aligned} \left| I_{12}\right|&\le C\left\| \nabla _\Gamma \mu _1\right\| _{{\textbf {L}}^\infty (\Gamma (t))}\left\| \nabla _\Gamma {{\textbf {V}}}\right\| \left\| {{\textbf {V}}}\right\| \\  &\qquad +C\left\| \nabla _\Gamma \Delta _\Gamma \varphi \right\| \left\| \nabla _\Gamma {{\textbf {V}}}_2\right\| _{{\textbf {L}}^4(\Gamma (t))}\left\| {{\textbf {V}}}\right\| _{{\textbf {L}}^4(\Gamma (t))}\\  &\qquad +C\left\| F''(\varphi _1)-\theta _0\right\| _{L^\infty (\Gamma (t))}\left\| \nabla _\Gamma \varphi \right\| \left\| \nabla _\Gamma {{\textbf {V}}}_2\right\| _{{\textbf {L}}^4(\Gamma (t))}\left\| {{\textbf {V}}}\right\| _{{\textbf {L}}^4(\Gamma (t))}\\  &\qquad +C\left\| \nabla _\Gamma \varphi _2\right\| _{{\textbf {L}}^\infty (\Gamma (t))}\left\| \varphi \right\| \left\| \nabla _\Gamma {{\textbf {V}}}_2\right\| _{{\textbf {L}}^4(\Gamma (t))}\left\| {{\textbf {V}}}\right\| _{{\textbf {L}}^4(\Gamma (t))}\\  &\qquad +C\left\| \nabla _\Gamma \mu _1\right\| ^\frac{1}{2}\left\| \mu _1\right\| _{H^3(\Gamma (t))}^\frac{1}{2}(\left\| {{\textbf {V}}}\right\| +\left\| {\mathcal {E}}_S({{\textbf {V}}})\right\| )\left\| {{\textbf {V}}}\right\| \\  &\qquad +C\left\| \nabla _\Gamma \Delta _\Gamma \varphi \right\| \left\| \nabla _\Gamma {{\textbf {V}}}_2\right\| ^\frac{1}{2}\left\| {{\textbf {V}}}_2\right\| _{{\textbf {H}}^2(\Gamma (t))}^\frac{1}{2}\left\| {{\textbf {V}}}\right\| ^\frac{1}{2}(\left\| {{\textbf {V}}}\right\| ^\frac{1}{2}+\left\| {\mathcal {E}}_S({{\textbf {V}}})\right\| ^\frac{1}{2})\\  &\qquad +C\left\| \nabla _\Gamma \varphi \right\| \left\| \nabla _\Gamma {{\textbf {V}}}_2\right\| ^\frac{1}{2}\left\| {{\textbf {V}}}_2\right\| _{{\textbf {H}}^2(\Gamma (t))}^\frac{1}{2}\left\| {{\textbf {V}}}\right\| ^\frac{1}{2}(\left\| {{\textbf {V}}}\right\| ^\frac{1}{2}+\left\| {\mathcal {E}}_S({{\textbf {V}}})\right\| ^\frac{1}{2})\\  &\qquad +C\left\| \varphi \right\| \left\| \nabla _\Gamma {{\textbf {V}}}_2\right\| ^\frac{1}{2}\left\| {{\textbf {V}}}_2\right\| _{{\textbf {H}}^2(\Gamma (t))}^\frac{1}{2}\left\| {{\textbf {V}}}\right\| ^\frac{1}{2}(\left\| {{\textbf {V}}}\right\| ^\frac{1}{2}+\left\| {\mathcal {E}}_S({{\textbf {V}}})\right\| ^\frac{1}{2})\\  &\le C(T)(1+\left\| \mu _1\right\| _{H^3(\Gamma (t))}+\left\| {{\textbf {V}}}_2\right\| _{{\textbf {H}}^2(\Gamma (t))}^\frac{1}{2}+\left\| {{\textbf {V}}}_2\right\| _{{\textbf {H}}^2(\Gamma (t))})(\left\| {{\textbf {V}}}\right\| ^2+\left\| \nabla _\Gamma \varphi \right\| ^2)\\  &\qquad +\frac{1}{4}\left\| \nabla _\Gamma \Delta _\Gamma \varphi \right\| ^2+\frac{\nu _*}{16}\left\| {\mathcal {E}}_S({{\textbf {V}}})\right\| ^2. \end{aligned}$$

Furthermore, recalling (5.11),

$$\begin{aligned} \left| I_{13}\right|&\le C(T)(\left\| \widehat{{\textbf {u}}}\right\| _{{\textbf {L}}^\infty (\Gamma (t))}\left\| \nabla _\Gamma {{\textbf {V}}}\right\| \left\| {{\textbf {V}}}\right\| +\left\| \varphi \right\| _{L^4(\Gamma (t))}\left\| \widehat{{\textbf {u}}}\right\| _{{\textbf {L}}^\infty (\Gamma (t))}\left\| \nabla _\Gamma {{\textbf {V}}}_2\right\| \left\| {{\textbf {V}}}\right\| _{{\textbf {L}}^4(\Gamma (t))}\\&\quad +\left\| {{\textbf {V}}}\right\| ^2\left\| \widehat{{\textbf {u}}}\right\| _{{\textbf {L}}^\infty (\Gamma (t))}) \\  &\le C(T)(\left\| {{\textbf {V}}}\right\| ^2+\left\| \nabla _\Gamma \varphi \right\| ^2)+\frac{\nu _*}{16}\left\| {\mathcal {E}}_S({{\textbf {V}}})\right\| ^2. \end{aligned}$$

Then, similarly

$$\begin{aligned} \left| I_{14}\right|&\le C\left\| \varphi \right\| _{L^4 (\Gamma (t))}\left\| {{\textbf {V}}}_2\right\| _{{\textbf {L}}^\infty (\Gamma (t))}\left\| \nabla _\Gamma \widehat{{\textbf {u}}}\right\| _{{\textbf {L}}^\infty (\Gamma (t))}\left\| {{\textbf {V}}}\right\| _{{\textbf {L}}^4(\Gamma (t))}+C(T)\left\| {{\textbf {V}}}\right\| ^2\\  &\quad +C(T)\left\| \varphi \right\| (1+\left\| {{\textbf {V}}}_2\right\| _{{\textbf {L}}^\infty (\Gamma (t))})\left\| {{\textbf {V}}}\right\| \\&\le C\left\| \nabla _\Gamma \varphi \right\| \left\| {{\textbf {V}}}_2\right\| _{{\textbf {H}}^2(\Gamma (t))}^\frac{1}{2}\left\| {{\textbf {V}}}\right\| ^\frac{1}{2}(\left\| {{\textbf {V}}}\right\| ^\frac{1}{2}+\left\| {\mathcal {E}}_S({{\textbf {V}}})\right\| ^\frac{1}{2})+C(T)\left\| {{\textbf {V}}}\right\| ^2\\  &\quad +C(T)\left\| \nabla _\Gamma \varphi \right\| \left\| {{\textbf {V}}}_2\right\| _{{\textbf {H}}^2(\Gamma (t))}^\frac{1}{2}\left\| {{\textbf {V}}}\right\| \\  &\quad \le C(T)(1+\left\| {{\textbf {V}}}_2\right\| _{{\textbf {H}}^2(\Gamma (t))}^\frac{1}{2}+\left\| {{\textbf {V}}}_2\right\| _{{\textbf {H}}^2(\Gamma (t))})(\left\| {{\textbf {V}}}\right\| ^2+\left\| \nabla _\Gamma \varphi \right\| ^2)+\frac{\nu _*}{16}\left\| {\mathcal {E}}_S({{\textbf {V}}})\right\| ^2, \end{aligned}$$

as well as, recalling (5.28) and (5.35),

$$\begin{aligned}&\left| I_{15}\right| +\left| I_{20}\right| \le C(\left\| v_{\textbf {n}}{\textbf {H}}\right\| _{{\textbf {L}}^\infty (\Gamma (t))}+\left\| \nabla _\Gamma \widehat{{\textbf {u}}}\right\| _{{\textbf {L}}^\infty (\Gamma (t))})\left\| {\textbf {J}}_{\rho _1}-{\textbf {J}}_{\rho _2}\right\| \left\| {{\textbf {V}}}\right\| \\  &\quad \le C(T)(\left\| \nabla _\Gamma \Delta _\Gamma \varphi \right\| +\left\| F''(\varphi )-\theta \right\| _{L^\infty (\Gamma (t))}\left\| \nabla _\Gamma \varphi \right\| +\left\| \varphi \right\| \left\| \nabla _\Gamma \varphi _2\right\| _{{\textbf {L}}^\infty (\Gamma (t))})\left\| {{\textbf {V}}}\right\| \\  &\quad \le C(T)(\left\| {{\textbf {V}}}\right\| ^2+\left\| \nabla _\Gamma \varphi \right\| ^2)+\frac{1}{8} \left\| \nabla _\Gamma \Delta _\Gamma \varphi \right\| ^2. \end{aligned}$$

Then, thanks to the regularity of $\nu$ and again to (2.8), (5.11) and (5.28),

$$\begin{aligned} \left| I_{16}\right| +\left| I_{21}\right|&\le C\left\| \varphi \right\| _{L^4(\Gamma (t))}(\left\| {{\textbf {V}}}_2\right\| _{{\textbf {W}}^{1,4}(\Gamma (t))}+\left\| \widehat{{\textbf {u}}}\right\| _{{\textbf {W}}^{1,4}(\Gamma (t))})\left\| {\mathcal {E}}_S({{\textbf {V}}})\right\| \\  &\le C(T)\left\| \nabla _\Gamma \varphi \right\| (1+\left\| {{\textbf {V}}}_2\right\| ^\frac{1}{2}_{{\textbf {H}}^1(\Gamma (t))}\left\| {{\textbf {V}}}_2\right\| ^\frac{1}{2}_{{\textbf {H}}^2(\Gamma (t))})\left\| {\mathcal {E}}_S({{\textbf {V}}})\right\| \\  &\le C(T)(1+\left\| {{\textbf {V}}}_2\right\| _{{\textbf {H}}^2(\Gamma (t))})\left\| \nabla _\Gamma \varphi \right\| ^2+\frac{\nu _*}{16}\left\| {\mathcal {E}}_S({{\textbf {V}}})\right\| ^2. \end{aligned}$$

Furthermore, concerning $I_{17}$, recalling (2.7), (2.8), and (5.28),

$$\begin{aligned} \left| I_{17}\right|&\le \left\| \nabla _\Gamma \varphi \right\| _{{\textbf {L}}^4(\Gamma (t))}(\left\| \nabla _\Gamma \varphi _1\right\| _{{\textbf {L}}^4(\Gamma (t))}+\left\| \nabla _\Gamma \varphi _2\right\| _{{\textbf {L}}^4(\Gamma (t))})(\left\| {{\textbf {V}}}\right\| +\left\| {\mathcal {E}}_S({{\textbf {V}}})\right\| \\  &\le C(T)\left\| \nabla _\Gamma \varphi \right\| ^\frac{1}{2}(\left\| \nabla _\Gamma \varphi \right\| ^\frac{1}{2}+\left\| \Delta _\Gamma \varphi \right\| ^\frac{1}{2})(\left\| {{\textbf {V}}}\right\| +\left\| {\mathcal {E}}_S({{\textbf {V}}})\right\| \\  &\le C(T)(\left\| {{\textbf {V}}}\right\| ^2+\left\| \nabla _\Gamma \varphi \right\| ^2)+\frac{\nu _*}{16}\left\| {\mathcal {E}}_S({{\textbf {V}}})\right\| ^2+\frac{\theta }{8}\left\| \Delta _\Gamma \varphi \right\| ^2. \end{aligned}$$

To conclude, terms $I_{18}$ and $I_{19}$ are straightforwardly estimated thanks to Poincaré’s inequality and (5.11), since

$$\begin{aligned}&\left| I_{18}\right| +\left| I_{19}\right| \\  &\quad \le C\left\| \varphi \right\| \left\| v_{\textbf {n}}{\textbf {H}}\right\| _{{\textbf {L}}^\infty (\Gamma (t))}\left\| \nabla _\Gamma {{\textbf {V}}}\right\| \\&\qquad +C\left\| \varphi \right\| \left\| \partial ^\circ _t\widehat{{\textbf {u}}}\right\| _{{\textbf {L}}^\infty (\Gamma (t))}\left\| {{\textbf {V}}}\right\| +C\left\| \varphi \right\| \left\| \widehat{{\textbf {u}}}\right\| _{{\textbf {L}}^\infty (\Gamma (t))}\left\| \nabla _\Gamma \widehat{{\textbf {u}}}\right\| _{{\textbf {L}}^\infty (\Gamma (t))}\left\| {{\textbf {V}}}\right\| \\  &\quad \le C(T)(\left\| \nabla _\Gamma \varphi \right\| ^2+\left\| {{\textbf {V}}}\right\| ^2)+\frac{\nu _*}{16}\left\| {\mathcal {E}}_S({{\textbf {V}}})\right\| ^2. \end{aligned}$$

Putting all these estimates together, we deduce from (5.34) that

$$\begin{aligned}&\frac{1}{2}\frac{d}{dt}\int _{\Gamma (t)}\rho _1\left| {{\textbf {V}}}\right| ^2d\sigma +\frac{11}{8}\nu _*\int _{\Gamma (t)}\left| {\mathcal {E}}_S({{\textbf {V}}})\right| ^2d\sigma \\  &\quad \le C(T)(1+\left\| \partial ^\circ _t\varphi _1\right\| ^2+\left\| \partial ^\circ _t{{\textbf {V}}}_2\right\| ^2+\left\| {{\textbf {V}}}\right\| _{{\textbf {H}}^2(\Gamma (t))}+\left\| \mu _1\right\| _{H^3(\Gamma (t))})(\left\| {{\textbf {V}}}\right\| ^2+\left\| \nabla _\Gamma \varphi \right\| ^2)\\  &\qquad +\frac{3}{8}\left\| \nabla _\Gamma \Delta _\Gamma \varphi \right\| ^2+\frac{\theta }{8}\left\| \Delta _\Gamma \varphi \right\| ^2, \end{aligned}$$

which, if we sum up with (5.33) and recall that $\rho _1\ge \rho _*$, becomes

$$\begin{aligned}&\frac{1}{2}\frac{d}{dt}\left( \int _{\Gamma (t)}\left( \rho _1\left| {{\textbf {V}}}\right| ^2+\left\| \nabla _\Gamma \varphi \right\| ^2\right) d\sigma \right) +\nu _*\int _{\Gamma (t)}\left| {\mathcal {E}}_S({{\textbf {V}}})\right| ^2d\sigma +\frac{1}{8}\left\| \nabla _\Gamma \Delta _\Gamma \varphi \right\| ^2+\frac{\theta }{4}\left\| \Delta _\Gamma \varphi \right\| ^2\\  &\quad \le C(T)(1+\left\| \partial ^\circ _t\varphi _1\right\| ^2+\left\| \partial ^\circ _t{{\textbf {V}}}_2\right\| ^2+\left\| {{\textbf {V}}}_2\right\| _{{\textbf {H}}^2(\Gamma (t))}+\left\| \mu _1\right\| _{H^3(\Gamma (t))})\\&\quad \left( \int _{\Gamma (t)}\rho _1\left| {{\textbf {V}}}\right| ^2d\sigma +\left\| \nabla _\Gamma \varphi \right\| ^2\right) . \end{aligned}$$

Now, thanks to (5.28), it holds

$$ \mathcal {H}:=1+\left\| \partial ^\circ _t\varphi _1\right\| ^2+\left\| \partial ^\circ _t{{\textbf {V}}}_2\right\| ^2+\left\| {{\textbf {V}}}_2\right\| _{{\textbf {H}}^2(\Gamma (t))}+\left\| \mu _1\right\| _{H^3(\Gamma (t))}\in L^1(0,T), $$

entailing by the Gronwall Lemma the desired continuous dependence estimate, leading to the uniqueness of strong solutions. This concludes the proof of the theorem.

## Data Availability

Data sharing not applicable to this article as no datasets were generated or analysed during the current study.

## References

[1] Abels, H., Depner, D., Garcke, H.: Existence of weak solutions for a diffuse interface model for two-phase flows of incompressible fluids with different densities. J. Math. Fluid Mech. **15**(3), 453–480 (2013)

[2] Abels, H., Garcke, H., Giorgini, A.: Global regularity and asymptotic stabilization for the incompressible Navier–Stokes–Cahn–Hilliard model with unmatched densities. Math. Ann. **389**, 1267–1321 (2024)

[3] Abels, H., Garcke, H., Poiatti, A.: Mathematical analysis of a diffuse interface model for multi-phase flows of incompressible viscous fluids with different densities. J. Math. Fluid Mech. **26**(2), 29–51 (2024)

[4] Abels, H., Garcke, H., Poiatti, A.: Diffuse interface model for two-phase flows on evolving surfaces with different densities: Local well-posedness. arXiv:2407.14941, (2024) 10.1007/s00526-025-03001-w PMC12050238 40330639

[5] Alphonse, A., Caetano, D., Djurdjevac, A., Elliott, C.M.: Function spaces, time derivatives and compactness for evolving families of Banach spaces with applications to PDEs. J. Diff. Equ. **353**, 268–338 (2023)

[6] Alphonse, A., Elliott, C.M., Stinner, B.: An abstract framework for parabolic PDEs on evolving spaces. Port. Math. **72**(1), 1–46 (2015)

[7] Aubin, T.: Nonlinear Analysis on Manifolds, Monge-Ampère Equations. Springer, New York (1982)

[8] Barrett, J. W., Garcke, H., Nürnberg, R.: Parametric finite element approximations of curvature-driven interface evolutions. In Geometric partial differential equations. Part I, volume 21 of Handb. Numer. Anal., pages 275–423. Elsevier/North-Holland, Amsterdam, [2020] (2020)

[9] Brandner, P., Reusken, A., Schwering, P.: On derivations of evolving surface Navier–Stokes equations. Interfaces Free Bound. **24**(4), 533–563 (2022)

[10] Caetano, D., Elliott, C.M.: Cahn–Hilliard equations on an evolving surface. European J. Appl. Math. **32**(5), 937–1000 (2021)

[11] Caetano, D., Elliott, C.M., Grasselli, M., Poiatti, A.: Regularization and separation for evolving surface Cahn–Hilliard equations. SIAM J. Math. Anal. **55**(6), 6625–6675 (2023)

[12] Dziuk, G., Elliott, C.M.: Finite element methods for surface PDEs. Acta Numer **22**, 289–396 (2013)

[13] Dziuk, G., Kröner, D., Müller, T.: Existence and uniqueness for scalar conservation laws on moving hypersurfaces. In Hyperbolic problems: theory, numerics, applications, volume 8 of AIMS Ser. Appl. Math., pages 775–782. Am. Inst. Math. Sci. (AIMS), Springfield, MO, (2014)

[14] Elliott, C.M., Sales, T.: Navier–Stokes–Cahn–Hilliard Equations on Evolving Surfaces. Published online first, Interfaces Free Bound. (2024)

[15] Gal, C.G., Giorgini, A., Grasselli, M., Poiatti, A.: Global well-posedness and convergence to equilibrium for the Abels–Garcke-Grün model with nonlocal free energy. J. Math. Pures Appl. **9**(178), 46–109 (2023)

[16] Gal, C.G., Poiatti, A.: Unified framework for the separation property in binary phase-segregation processes with singular entropy densities. European J. Appl. Math. **36**(1), 40–67 (2025)

[17] Giorgini, A.: Well-posedness of the two-dimensional Abels–Garcke-Grün model for two-phase flows with unmatched densities. Calc. Var. Partial Diff. Equ. **60**(3), 100–40 (2021)

[18] Jankuhn, T., Olshanskii, M.A., Reusken, A.: Incompressible fluid problems on embedded surfaces: modeling and variational formulations. Interfaces Free Bound. **20**(3), 353–377 (2018)

[19] Kenmochi, N., Niezgodka, M., Pawlow, I.: Subdifferential operator approach to the Cahn–Hilliard equation with constraint. J. Differential Equations **117**(2), 320–356 (1995)

[20] Olshanskii, M.A., Reusken, A., Zhiliakov, A.: Tangential Navier–Stokes equations on evolving surfaces: analysis and simulations. Math. Models Methods Appl. Sci. **32**(14), 2817–2852 (2022)

